# What are the effects of Teach For America on Math, English Language Arts, and Science outcomes of K–12 students in the USA?

**DOI:** 10.4073/csr.2018.7

**Published:** 2018-06-25

**Authors:** Herbert Turner, Mackson Ncube, Annette Turner, Robert Boruch, Nneka Ibekwe

## Abstract

This Campbell systematic review examines the impact of Teach For America (TFA) on learning outcomes. Four studies were included in the review.

Studies had to be a quantitative evaluation of the effects of TFA on K–12 student academic outcomes. Studies also had to use a research design which: 1. allowed valid causal inferences about TFA's effects, 2. targeted participants K–12 students taught by TFA corps members or TFA alumni in the USA, 3. compared TFA corps members to novice teachers, or compared TFA alumni with veteran teachers, and 4. reported at least one academic student outcome in math, ELA, or science domains.

A total of 919 citations were retrieved on TFA, of which 24 studies were eligible for review. However, when the research design and study quality along with types of TFA corps members and non‐TFA teachers compared were reviewed, the evidence base for estimating the effects of TFA on student academic outcomes was reduced to just four studies.

There is no significant effect on reading from teaching by TFA corps members in their first or second year of teaching elementary‐grade students (PreK – grade 5) compared to non‐TFA teachers who are also in their first or second year of teaching elementary‐grade students. There is a small positive effect for early elementary‐grade students (PreK to grade 2) in reading but not in math.

However, given the small evidence base, these findings should be treated with caution.

**Plain language summary:**

**There are too few well‐designed studies to know the effects of Teach For America on Math, English Language Arts, and Science outcomes of K–12 students in the USA**

Teach For America (TFA) is an alternate route teacher preparation program that aims to address the decades‐long shortage of effective teachers in many rural and urban public schools for kindergarten through 12th grade (K–12), that serve the highest proportions of high‐poverty students across the USA. This review finds that there are very few studies – just four – which reliably measure the effects of TFA on learning outcomes, so that no firm conclusions may be drawn.

**What is this review about?**

This systematic review evaluated the impact of TFA prepared teachers (corps members) relative to novice teachers and alumni relative to veteran teachers on K‐12 student outcomes in math, English Language Arts (ELA), and science.

**What is the aim of this review?**

This Campbell systematic review examines the impact of Teach For America on learning outcomes. Four studies were included in the review.

**What are the main findings of this review?**

Studies had to be a quantitative evaluation of the effects of TFA on K‐12 student academic outcomes. Studies also had to use a research design which: 1. allowed valid causal inferences about TFA's effects, 2. targeted participants K–12 students taught by TFA corps members or TFA alumni in the USA, 3. compared TFA corps members to novice teachers, or compared TFA alumni with veteran teachers, and 4. reported at least one academic student outcome in math, ELA, or science domains.

A total of 919 citations were retrieved on TFA, of which 24 studies were eligible for review. However, when the research design and study quality along with types of TFA corps members and non‐TFA teachers compared were reviewed, the evidence base for estimating the effects of TFA on student academic outcomes was reduced to just four studies.

There is no significant effect on reading from teaching by TFA corps members in their first or second year of teaching elementary‐grade students (PreK – grade 5) compared to non‐TFA teachers who are also in their first or second year of teaching elementary‐grade students. There is a small positive effect for early elementary‐grade students (PreK to grade 2) in reading but not in math.

However, given the small evidence base, these findings should be treated with caution.

**What do the findings of this review mean?**

TFA is the most evaluated program of its kind. Multiple quasi‐experimental and experimental studies have been conducted on its effectiveness in improving student outcomes. However, this systematic review found that only a small number of these studies (1) met the evidence review standards and (2) compared the same type of TFA corps members and non‐TFA teachers. So it is not possible to draw firm policy conclusions.

Future research can contribute to this evidence base by designing, implementing, and reporting experiments and quasi‐experiments to meet objective extant evidence standards and by comparing the same types of TFA and non‐TFA teachers so that effect sizes can be included in a future systematic review and meta‐analysis.

**How up‐to‐date is this review?**

The review authors searched for studies published up to January 2015. This Campbell systematic review was published in June 2018.

## Background

### Description of the condition

Research shows that there is a shortage of effective teachers in many rural and urban K–12 public schools serving the highest proportions of high‐poverty students across the United States (Clotfelter, Ladd, & Vigdor, 2006; Monk, 2007; Peske & Haycock, 2006)—a shortage that has persisted for decades (Darling‐Hammond, 1984; Ingersoll 2001; Ingersoll & Perda, 2010). In the past 10 years, *alternative route teacher preparation programs* aiming to address this shortage proliferated across the United States (Kane, Rockoff, & Staiger, 2007). These programs seek to increase the supply of teachers more rapidly than traditional teacher preparation programs (Blazer, 2012; Hess, 2002; Raymond & Fletcher, 2002). Although their requirements vary widely, most are shorter, less expensive, and more practically oriented than traditional teacher preparation programs (Blazer, 2102). These programs also vary widely in their selection criteria for teacher candidates, approach to training these candidates, notoriety among education stakeholders, and evidence of effectiveness (Constantine et al., 2009; Hess, 2002; Kaine et al., 2007).

Teach For America (TFA) is a nation‐wide alternate route teacher preparation program designed to address the shortage of effective teachers, specifically in high‐poverty rural and urban schools across the United States (Teach For America [TFA], 2010). We assert that TFA should be systematically reviewed for several reasons:

It is the largest recipient of philanthropic funding for K–12 teacher recruitment (Blazer, 2012; Mead, 2015), with a present budget of $300 million through philanthropic and government support (Baker, 2016).

TFA is a significant source of new teachers for K–12 education: Since 1990, TFA has recruited, selected, trained, placed, and supported approximately 40,000 new public school teachers (or corps members) in the highest‐poverty school districts in rural and urban areas.

As noted by Williams (2014), since its inception in 1990, TFA has been one of the most publicly visible and widely debated alternative route teacher preparation programs.

As there have been multiple quasi‐experimental and experimental studies on the effectiveness of TFA in improving student outcomes, there is now a sufficient amount of evidence to be systematically reviewed and, if appropriate, meta‐analyzed.

### Description of the intervention

TFA is an alternative route teacher preparation program that selectively recruits both college undergraduates and graduates—many from top colleges—and professionals to teach in low‐income schools (Clark, Isenberg, Liu, Makowsky, & Zukiewicz, 2015). The goal that corps members will become effective teachers “who lead their students to significant academic achievement” is explicitly stated in TFA's mission of eliminating educational inequity in U.S. public schools (TFA, 2010, p. 8). To achieve this goal, TFA uses a data‐driven program model comprising (1) recruitment, (2) a rigorous selection process, (3) intensive pre‐service training for selected corps members, (4) two years of ongoing professional development for corps members, and (5) programming that fosters alumni leadership after TFA corps members have completed their two‐year commitment (TFA, 2010). Each component is described in more detail below.

#### Recruitment

**Figure 1:**
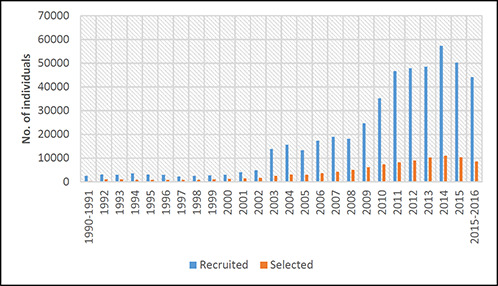
Number of individuals recruited versus number of individuals selected to become corps members during the past 25 years of TFA's history Source: Compiled fromdata reported in *Teach For America (2010)*

TFA annually recruits graduates and undergraduates at college campuses throughout the United States (see Figure 1). In 2012, TFA recruited approximately 5% of the graduating classes of 135 colleges and universities (Clark et al., 2015). When TFA began in 1990, the organization recruited 2,500 applicants; recruitment peaked at 57,000 applications in 2014 (see Figure 1). TFA places an emphasis on recruiting ethnically and economically diverse corps members to teach difficult‐to‐staff subjects such as science, math, and special education (Clark et al., 2015).

#### Selection

TFA's selection process aims to identify candidates who are most likely to succeed in the classroom. Roughly 25% of recruits become corps members (see Figure 1). The selection process includes a writing activity, telephone interview, sample teaching lesson plan, group discussion, and in‐person interview. Potential corps members are evaluated on their competency in such areas as academics, leadership, critical thinking, ability to influence and motivate others, organizational ability, respect for students and families in low‐income communities, and perseverance (TFA, 2010). Selected corps members receive a five‐week intensive summer training and agree to teach in their assigned school for at least two years. Those who complete the two‐year commitment become alumni and are eligible to be part of the TFA community, with continued access to resources and support for alumni (see Programming for Alumni, below).

#### Pre‐service training

The five‐week pre‐service summer training covers (1) instructional and pedagogical philosophies and practices, (2) classroom management skills, (3) attitudes toward teaching, and (4) academic ability. TFA hypothesizes that these skills and attitudes have a positive and meaningful effect on students’ academic achievement. As a corollary, TFA also hypothesizes that this effect is larger for students whose instruction is provided by a TFA corps member or TFA alumni than by a non‐TFA corps member or TFA alumni.

#### Ongoing professional development

TFA corps members continue to receive training and support throughout their two‐year teaching commitment to help them further develop skills and attitudes introduced during the pre‐service training. This ongoing professional development includes observation and coaching from program directors; access to online classroom resources, advice, and community support; and self‐directed online learning on a private, secure website for corps members and alumni.

#### Programming for alumni

At the end of their two‐year assignment, TFA alumni are encouraged to continue to engage in meaningful ways to advance the mission of TFA and become influential education leaders and advocates for children. TFA alumni have access to teaching resources and the support of the TFA community as they continue their professional careers.

### How the intervention might work

The hypothesized TFA Theory of Change, distilled from the literature (including TFA's 2010–15 Business Plan [TFA, 2010]), is as follows:

- TFA recruits1 and selects applicants using a selection model based on the organization's data, which include measures of TFA student achievement and TFA corps member characteristics. Using these measures, TFA analyzes the relationship between TFA student achievement and TFA corps member characteristics and how these characteristics correlate with implementing a Teaching as Leadership approach in the classroom.
- TFA trains selected individuals, starting with a five‐week intensive summer institute followed by fieldwork opportunities, such as classroom observations and delivery of instruction, before the initial teaching year. TFA training is guided by the Teaching as Leadership approach that codifies for corps members a goal‐oriented approach to teaching in the classroom.
- TFA provides ongoing peer mentoring and professional development, including regular feedback on the impact of corps members’ teaching on the classroom and on student achievement.
- After completing their two‐year teaching commitment, alumni who continue to teach are eligible to continue to receive access to TFA professional development and leadership resources.

**Figure 2:**
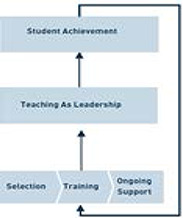
TFA Theory of Change. Reprinted from *Building the Movement to Eliminate Educational Inequity* (Teach For America, 2010). Used with permission.

The TFA Theory of Change is summarized and depicted in Figure 2.

### Why it is important to do the review

Conducting a systematic review of TFA is important for four reasons. First, previous primary research on TFA has yielded inconsistent results with positive and statistically significant effects for some academic outcomes and null effects for other academic outcomes; these effects have also varied by elementary and middle school grades. Second, previous reviews have consisted primarily of narrative reviews. Narrative reviews are not a substitute for systematic reviews because the former does not rely on systematic procedures to limit bias the way the latter does. Third, reliable and valid systematic evidence is needed to address any future scale‐ups of TFA. A systematic review when accompanied by a meta‐analysis of a corpus of studies can provide an empirical projection of what the overall effect of TFA would be if it implemented in multiple sites. Fourth, gaps in research knowledge about TFA can be identified through a systematic review. Each of these reasons is described next.

#### Previous primary research on TFA has not yielded consistent results

Educational researchers and economists alike have investigated the effects of TFA corps members and alumni on student academic outcomes, using correlation, quasi‐experimental, and randomized controlled trial designs. There have been vigorous debates about TFA's effectiveness based on these primary studies, which, being single studies, at best have questionable generalizability. The quasi‐experimental studies on TFA produced mixed signals. In contrast, the experimental studies have consistently produced a positive and statistically significant effect of TFA on student math achievement, though they found no discernable effect on reading achievement (Clark et al., 2013; Decker, Mayer, & Glazerman, 2004; Laczko‐Kerr & Berliner, 2002; Raymond, Fletcher, & Luque, 2001; Seftor & Mayer, 2003).

Until a systematic review of these studies is conducted, we do not know the average effect of TFA across these experiments. Furthermore, the average effect may vary according to academic outcome, grade level, teacher experience, and teacher certification status, and this variation can only be investigated through a meta‐analysis. Also, the methodological quality of the quasi‐experimental studies (e.g., the establishment of baseline equivalence between groups in the analysis sample) and experimental studies (e.g., high attrition disrupting random assignment) has not been systematically and rigorously evaluated using Campbell Collaboration (Campbell) systematic review methods. By systematically reviewing these primary studies on TFA, we can apply methods designed to limit the bias in the retrieval, appraisal, and statistical synthesis of the study findings (Cooper, 2010; Petticrew & Roberts, 2006). This application can lead to more systematic, focused, and conclusive results on the effects of TFA on student academic achievement.

Using Campbell systematic review and meta‐analysis methods, we empirically investigated whether effect sizes reported in primary studies were consistent and could be generalized across populations and settings. Our goal was to use meta‐analysis, after we systematically reviewed studies, to synthesize study results, potentially increase the power and precision of the overall estimated effect of an intervention by pooling primary study effect sizes^2^ (Cohn & Becker, 2003), and possibly enhance the generalization of this overall estimated effect (Chalmers & Altman, 1995; Cooper, 2010; Petticrew & Roberts, 2006).

#### Narrative reviews are not a substitute for systematic reviews

The narrative reviews of quasi‐experimental studies and experimental studies that researchers have conducted on the effects of TFA on K–12 students’ academic outcomes are helpful in gaining an approximate idea of the amount of agreement or disagreement of treatment effects across studies. They also help us understand what treatment effects look like across different samples. However, the primary limitations of a narrative review are the lack of orderly and transparent identification of study characteristics and effect sizes and the lack of statistical synthesis of these effect sizes.

It is difficult, if not impossible, for narrative reviews to cognitively and systematically manage and control for the many sources of variation in primary study characteristics and effect sizes (Chalmers & Altman, 1995; Cooper, 2010; Petticrew & Roberts, 2006). Variationsarise from the different time periods, within‐study sampling, sample characteristics, group comparisons, outcome measures, and designs. Reporting of such studies, if not handled systematically, could produce the appearance of conflicting results, or could produce consistent results without empirical information across studies to understand why.

In contrast, a systematic review transparently and systematically combs through the evidence. It also controls for study quality, and, when appropriate, statistically synthesizes the results to present findings with greater clarity and less potential bias than narrative (or literature) reviews (Chalmers & Altman, 1995; Cooper, 2010; Petticrew & Roberts, 2006).

#### Reliable and valid systematic evidence is needed to address future scale‐up of TFA

In 2010, there were enough randomized control trials and matched comparison studies with substantially positive and statistically significant findings to motivate the U.S. Department of Education's Office of Innovation and Improvement to award TFA a $50 million Investing in Innovation (i3) Scale‐up grant. The Department's goal was to provide TFA with funding to scale up nationally at the elementary school level in Fall 2010.^3^ TFA used the funds to more than double its corps members from 7,300 to 15,000 teachers and increase its presence from 46 to 60 urban and rural regions across the country. By the end of 2015, TFA teachers were reaching nearly 1 million students in some of our country's highest‐need communities

The randomized controlled trials and matched comparison studies were presented in a narrative review, by the i3 grant proposal authors, to make the case for the i3 Scale‐up funding. However, this i3 award and narrative revieware not a substitute for using Campbell systematic review methods to objectively assess the quality of randomized controlled trials and quasi‐experimental studies and to synthesize the effect sizes in order to estimate the average effect of TFA on student academic outcomes. In 2015, TFA also received philanthropic support from the Walton Foundation, who pledged an additional $50 million to fund TFA's operations.

These substantial governmental and philanthropic financial investments in TFA underscore the need for dependable and generalizable evidence regarding its effectiveness—as, given the continued shortage of effective teachers in high‐poverty rural and urban schools, it is reasonable to predict that the demand for alternative route teacher preparation programs such as TFA will increase, not decrease. This systematic review is an important first step toward that goal.

#### A TFA systematic review and meta‐analysis is an important benchmark

Mathematica researchers recently reported the results of the i3 Scale‐up impact evaluation of TFA (Clark et al., 2015). They evaluated the effects of TFA on student academic outcomes with a randomized controlled trial (RCT) in the elementary school grades. Appropriately synthesized effect sizes of individual RCTs in this Campbell systematic review could serve as the “maintenance” benchmark for the i3 Scale‐up RCT on TFA. Specifically, the effects reported in RCT studies conducted before the TFA scale‐up can be compared to effects in the i3 Scale‐up RCT to determine whether the pre‐scale‐up effects were larger, smaller, or maintained at the end of the TFA scale‐up. The comparison of pre‐scale‐up average effect sizes (from this Campbell systematic review) to scale‐up effect sizes (from the i3 Scale‐up) can make a significant contribution to the TFA knowledge base.

One of the primary challenges associated with scaling‐up interventions is maintaining the effectiveness of the intervention while going to scale (Klingner, Boardman, & McMaster, 2013). This systematic review also creates an empirical database of TFA effect sizes—and study characteristics—that can be used to summarize the empirical landscape of the highest‐quality research on TFA, based on Campbell systematic review standards, that readers could compare to the scale‐up findings.

Lastly, the systematic review can be used by readers to compare effect sizes from the scale‐up study to average effect sizes of other studies in the systematic review.

#### Gaps in research knowledge can be identified

During the past 20 years, researchers have conducted a substantial number of quantitative studies using a wide range of methodologies. The empirical database that results from the systematic review process will allow us to identify gaps in knowledge about the effects of TFA. An important purpose of a systematic review is to assess what is known and not known about the effects of an intervention in a particular area of inquiry (Hunt, 1997)—in this case, TFA.

## Objectives

The purpose of this research is to systematically review the TFA literature on the effects of TFA corps members and alumni on student academic outcomes. The review used systematic procedures that limit bias in the retrieval, critical appraisal, synthesis, and reporting of quasi‐experimental and experimental studies that examine the effects of TFA on K–12 student academic outcomes in math, English language arts (ELA), and science as reported in the peer‐reviewedliterature and grey literature during the past 20 years.^4^

To aid education policymakers and stakeholders (including researchers) in using the review results, we organized the research questions according to the policy relevance and methodological issues raised in our review of the literature on the effectiveness of TFA:

- What are the study characteristics of RCTs and quasi‐experimental designs (QEDs) conducted on TFA that met our inclusion criteria and were reported in this systematic review?
- What are the sample characteristics of the schools, teachers, and students on RCTs and QEDs that met our inclusion criteria and were reported in this systematic review?
- What are the main effects of TFA corps members on elementary school students in math, ELA, or science outcomes by research design?
- What are the main effects of TFA corps members on middle school students in math, ELA, or science outcomes by research design?
- What are the main effects of TFA corps members on high school students in math, ELA, or science outcomes by research design?
- Are the main effects estimated by research design similar enough to be combined? If so, what is the combined main effect of TFA at each grade level and corresponding outcome?
- How do the magnitude and statistical significance of the main effect of TFA change when controlling for the following teacher characteristics separately, in a moderator analysis?
  ∘ TFA candidate status (e.g., corps member or alumnus)
  ∘ Teacher certification status (e.g., traditionally certified, alternatively certified, or not certified)
  ∘ Teacher average years of teaching experience
- Is there sufficient fidelity of implementation information reported in TFA studies? If so, to what extent does the main effect of TFA differ by fidelity of TFA implementation?
- Is there sufficient information on teacher turnover in TFA studies to evaluate TFA's main effect on teacher retention? If so, what is the main effect on teacher retention?
- Is there sufficient information on teacher leadership, content knowledge, years of teaching experience, or overall academic ability to evaluate TFA's main effect on teacher quality? If so, what is the main effect on teacher quality?
- Is there sufficient cost information in TFA studies to evaluate whether the literature reports TFA as cost‐effective? If so, is TFA reported to be cost‐effective?
- This is a sentence with normal text style. This is a sentence with normal text style.

## Methods

Broadly speaking, to be included in this review, a study was required to use an RCT or QED design to produce the average treatment effects (ATE) of the TFA corps members or TFA alumni on at least one reliable and valid K–12 student academic outcome in public schools in the United States. RCTs that met these requirements had to meet the attrition standards, and QEDs that met these requirements had to meet the baseline equivalence standards at the unit of assignment and unit of analysis if these units were different (i.e., unit of random assignment was schools but unit of analysis was students).

### Criteria for considering studies for this review

The specific inclusion criteria applied to studies retrieved from the systematic search are described in what follows.

#### Types of studies

To be included in this review, a study had to be a quantitative study that examined the effects of TFA on student academic outcomes. Qualitative research, commentary, editorials, surveys, and written opinions about the effects of TFA were excluded from the review.

This review included studies with research designs that, when implemented well, are capable of generating data that can be used to make causal inferences about the ATE of TFA on student academic outcomes (Bloom, 2005; Boruch, 1997). Designs that met these criteria were RCTs and QEDs. We further limited the eligible designs for this review to RCTs where random assignment was used to form intervention and comparison groups, and to QEDs where non‐random methods (such as matching or other statistical methods) were used to form a counterfactual group that is comparable to the intervention group on measured characteristics.

Although Regression Discontinuity Designs (RDDs) and Single‐Case Designs (SCDs) generate data that can be used to make causal inferences, they were excluded from this review because statistical methods for incorporating RDD and SCD data into meta‐analyses are, to the best of our knowledge, not well‐established. For example, the Campbell Collaboration's Methods Policy Briefs do not address the statistical synthesis of RDDs and SCDs. Furthermore, the results of our literature search, for protocol development, found that RDDs were rare (only one identified) and SCDs were non‐existent.

Studies that used research designs that lacked a comparison group, such as single‐group “pretest/posttest” designs, were also excluded from the review. Designs without a comparison group cannot rule out a competing explanation for observed differences between intervention and comparison groups on an outcome (Furberg & Furberg, 2007; Mosteller & Boruch, 2002; Shadish, Cook, & Campbell, 2002).

Studies that use value‐added approaches to estimate the effect of TFA on student outcomes were eligible for inclusion in this study, provided that the study author (1) reported the data needed to estimate ATE or (2) responded to our request for this information. One reason for these additional inclusion criteria for value‐added estimates is that the “validity” of value‐added research in producing reliable and unbiased estimates of the treatment effect is not well‐established and is a matter of considerable debate (Andrabi, Das, Khwaja, & Zajonc, 2011), and methods for statistical synthesizing value‐added estimates are not as well‐established as those for synthesizing ATE estimates.

#### Types of participants

We included studies with participants who were K–12 students taught by TFA corps members (who are in the process of completing their two‐year TFA commitment), TFA alumni (who have completed their two‐year TFA commitment but continue to teach), and non‐TFA teachers in rural and urban public schools in the United States. At the time of the intervention, the teachers in the treatment condition were either TFA corps members or TFA alumni; the control condition included non‐TFA teachers who never participated in TFA. Non‐TFA teachers varied in their years of teaching experience and certification status. During the time frame of the study, all students had a teacher who met the eligibility criteria for TFA corps members or TFA alumni or for non‐TFA teachers.

#### Types of interventions

The TFA intervention condition included TFA corps members and TFA alumni, and the non‐TFA comparison condition included teachers who never participated in TFA. Teachers in the comparison condition did not receive preparation or training in programs associated with TFA, and they varied in their certification status (e.g., traditional, alternative, emergency, uncertified). To be included in the review, the study included a TFA condition and a non‐TFA condition. Studies that created an intervention group by bundling the TFA corps members or TFA alumni with teachers trained in other alternative route teacher preparation programs, such as the New York Teaching Fellows Program, were excluded from the review; when TFA is bundled with other alternative route programs, the effect of TFA teachers cannot be separated from the effect of other alternatively prepared teachers in the intervention group.

#### Types of outcome measures

##### Types of outcome measures for students

The review included studies with at least one academic student outcome in math, ELA, or science domains. Student outcomes in other non‐academic (or non‐cognitive) domains were documented using the study coding guide but were not reported in the review. Multiple types of outcome measures were documented using the study coding guide, although the primary types that we encountered were state assessments, end‐of‐course assessments, and other standardized assessments.

Eligible assessments were included in the review, provided that they were administered as intended and were consistently administered across treatment and control groups. Non‐standardized assessments, such as researcher‐developed assessments, were also eligible for inclusion in the review. However, a study was required to report evidence that the measure met three criteria:

- The assessment exhibits face validity and sufficient reliability. For example, a description of the assessment showing that the measure was clearly defined and measures the construct it is supposed to measure was accepted as evidence of face validity for this review. Reliability evidence could come in the form of internal consistency, test‐retest reliability, or inter‐rater reliability.
- The study reported evidence that the assessment of the outcome measure does not closely resemble aspects of the intervention. For example, the measure could not have items or materials that intervention teachers had access to through their TFA training materials but that comparison teachers did not.
- The outcome measures were administered the same way in the treatment and comparison conditions.

##### Types of outcome measures for teachers

- **Teacher retention.** To be an eligible outcome used in the assessment of TFA's effect on teacher retention, a study was required to report whether treatment and control group teachers were in the school, district, or state at the beginning and end of the study. The purpose of this requirement was to differentiate *teacher retention* (whether a teacher remains in a school, district, or state) from *teacher attrition* (whether a teacher remains in a study). The data on teacher retention were required to be collected consistently in both treatment and control groups.^5^
- **Teacher leadership.** There is no single definition of “teacher leadership,” but one that comes closest to aligning with the TFA framework is teachers who take on leadership roles and decision‐making responsibilities that extend beyond the school or district administrative team (Abbott, 2014). Reliable and valid measures designed to tap into this construct were eligible for the review. Studies were reviewed for having at least one teacher outcome on teacher leadership, which is a key mediator between TFA teacher training and student achievement in the TFA Theory of Change; if so, this study and the corresponding teacher outcome were included in the review. Additional teacher outcomes included in the review were content knowledge, years of teaching experience, and overall academic ability.
- **Content knowledge.** To be eligible for the review, a content knowledge outcome had to measure whether a teacher had a solid background in the subject or content area in which they teach (e.g., math, reading, science), as exhibited by a college minor or major in this subject or content area.
- **Teaching experience.** To be eligible for the review, a teaching experience outcome had to measure the teacher's total number of years of classroom teaching experience in the field.
- **Academic ability.** To be eligible for the review, an academic ability outcome was required to tap into the construct of teacher academic skills as measured by SAT scores, ACT scores, high school grade point average, selectivity of the college attended, college grade point average, or Praxis scores.

##### Validity criteria for student and teacher outcomes

When reviewing eligible outcome measures for reliability and validity, we applied the definitions and thresholds reported in the What Works Clearinghouse^TM^ (WWC) *Procedures and Standards Handbook, Version 3.0* (WWC, 2014, p. 16, section 4). For example, thresholds for the psychometric properties that determine the reliability of an outcome measure were based on the *WWC Evidence Standards for Group Design, Version 3.0*, which require (a) internal consistency of 0.50 or higher, (b) temporal stability/test‐retest reliability of 0.40 or higher, or (c) inter‐rater reliability of 0.50 or higher. Many of the teacher outcomes just discussed would qualify for this review under the standard educational measure criteria— that is, they are widely recognized heuristically (rather than psychometrically) as reliable and valid. Other teacher outcomes (such as SAT, ACT, and Praxis) qualified for this review because as standardized assessments they are assumed to be reliable and valid.

#### Duration of follow‐up

During the meta‐analysis, if a study reported multiple follow‐ups, we controlled for study‐to‐study differences in the follow‐up period by meta‐analyzing studies with the same follow‐up periods. Studies with one‐year follow‐up outcome were meta‐analyzed on that outcome together, studies with two‐year follow‐up outcome were meta‐analyzed on that outcome together, and so on.

Studies were excluded from the review if the minimum student exposure to a TFA corps member or TFA alumni was less than one school year. Studies were also excluded if the students in the treatment groups and comparison groups did not have comparable times of exposure to teachers.^6^

#### Types of settings

The review included studies that took place in K–12 public schools (including charter schools) in the United States. This focus is consistent with the K–12 implementation of TFA in public, rather than private, schools. However, we did encounter a study that included pre‐K along with grades K–5 when estimating the effects of TFA on student outcomes, though the researchers also estimated the effects for grades 3–5 separate from the effects for pre‐K. Thus, we included this study in the review for the estimated effect of TFA on student outcomes in grades 3–5.

### Search methods for identification of studies

The goal of the literature search, consistent with the Campbell Information Retrieval Policy Brief, was to identify all eligible studies on the effectiveness of TFA that are formally published (peer‐reviewed literature) and informally published (grey literature). This involved developing search strategies that were efficient—that is, retrieving relevant studies while screening out irrelevant studies to minimize bias. With this goal in mind, the final search strategy was developed in consultation with an academic librarian at the University of Pennsylvania.

The literature search was implemented by (1) searching electronic databases, (2) searching the grey literature, where studies are published informally, (3) soliciting a random sample of previous authors of TFA studies, and (4) manually scanning the Table of Contents of the 2014 and 2015 issues of journals where TFA effectiveness studies were published previously. For study relevance, electronic searches were limited to retrieving articles published (formally or informally) between 1994 and 2015.

#### Electronic searches

For the main electronic database search, we used the ProQuest search engine, which provides the capability to search 52 databases that index studies published formally and informally in a range of disciplines, including education, economics, psychology, and sociology. Using ProQuest, we searched each of the following databases separately:

- ERIC
- PsycINFO
- EconLit
- Sociological Abstracts
- PAIS International
- ProQuest Dissertations and Theses: UK and Ireland
- ProQuest Dissertations and Theses Global
- Worldwide Political Science Abstracts

Based on the search terms we developed, we organized the search according to the following four domains

1. **Search 1: Program name.** Used the search term “Teach For America” to search the Title and Abstract only.
2. **Search 2: Academic outcomes.** Developed search terms for the primary student academic outcomes that TFA is theorized to affect: math, ELA, and science.
3. **Search 3: Target population.** Developed search terms for the student populations served by TFA.
4. **Search 4: Research design.** Developed “research design” search terms to identify RCTs, QEDs, systematic reviews, and meta‐analyses.

Search terms were combined across domains with the Boolean “AND” operator to form the complete search strategy. Within domains, the Boolean “OR” operator was used to connect multiple keywords. An asterisk “*” was added to the end of the term to search for all variants that included the root term (e.g., “academic success” or “academic successes” and so on). The search terms were combined to form a search strategy as follows:

■ *Search 1:* (TI [“Teach For America”] OR AB [“Teach For America” OR “TFA Corps”])
  AND
■ *Search 2:* (“academic achievement” OR “academic success*” OR “grade level” OR “grading” OR “academic ability” OR “attainment” OR “failure” OR “educational indicator*”)
  AND
■ *Search 3:* (“kindergarten” OR “elementary school*” OR “primary school*” OR “high school*” OR “public school*”)
  AND
■ *Search 4:* (“random assignment” OR “randomized experiment” OR “experiment*” OR “experimental design” OR “control group” OR “non‐experiment” OR “non‐experimental” OR “quasi‐experiment” OR “quasi‐experimental” OR “comparison group” OR “matched comparison group” OR “matched comparison” OR “matched groups” OR “statistical matching” OR “propensity score matching” OR “systematic review” OR “review” OR “meta‐analysis” OR “research synthesis” OR “research review”)
■ **Date Delimiter:** Publication Date = 1 Jan 1994 – 2015 (used across Search 1–Search 4)

The above search strategy was implemented with ProQuest for each database (ERIC, PsycINFO, and so on) separately. After each search, the results were reviewed, and the database thesaurus was consulted to determine whether additional or other descriptors could be used to improve the identification of effectiveness studies on TFA. We found that the original search strategy was optimal and did not require modification. For example, we tested the use of thesaurus descriptors, grade‐level filters, and publication‐type filters. However, modifying the search strategy to include these did not identify additional studies beyond what we identified using the original search strategy.

We also searched three databases that are not part of ProQuest: JSTOR, Academic Search Premier, and Education Next/Full Text. When searching these databases, we adapted the search terms because the original search syntax did not work as well as it did for the databases searched through ProQuest. These adaptations and the search results are documented in Appendix A.1.

#### Searching other resources

To identify studies published informally, we conducted a five‐step grey literature search process by searching the following:^7^

- Grey literature databases
- General and targeted websites
- Conference presentation databases
- Existing narrative (or literature) reviews
- Google

We searched three grey literature databases: PolicyFile, PsycEXTRA, and OpenGrey.eu.

The general and targeted websites we searched included those for organizations that conduct policy research across many areas of education (see Table 1).

**Table 1:** General websites

Abt Associates	Hoover Institute
Alliance for Excellent Education	Mathematica Policy Research
American Educational Research Association	MDRC
American Enterprise Institute	National Assoc. of State Boards of Education
American Institutes for Research	National Governors’ Association
Best Evidence Encyclopedia	Policy Archive
Brookings Institution	Policy Study Associates
Carnegie Corporation of New York	RAND
Center for Research and Reform in Education	Regional Educational Laboratories
Congressional Research Service	SRI
Government Accountability Office	Thomas B. Fordham Institute
Grants and contracts awarded by IES	Urban Institute
Heritage Foundation	

We also targeted websites of organizations that have a focus on teacher education research or teacher effectiveness research, or have conducted TFA research in the past (see Table 2).

**Table 2:** Targeted websites

After‐School Alliance	Database of Abstracts of Reviews of Effects
Campbell Collaboration	Florida Center for Reading Research
Carnegie Corp. for the Advancement of Teaching	Harvard Family Research Project
Center for Social Organization of Schools	Institute for Higher Education Policy
Chapin Hall Center for Children	Inst. for Public Policy and Social Research
CINAHL	Natl. Assoc. of State Directors of Career Tech. Ed.
Cochrane Central Register of Controlled Trials	NBER Working Papers
Cochrane Database of Systematic Reviews	

To ensure that relevant conference presentations were reviewed for inclusion in the meta‐analysis, we searched the EditLib and “Index of Conference Proceedings” databases for conference abstracts, using search criteria similar to those for the main database search. We found, however, that the “Index of Conference Proceedings” was not conducive to a structured keyword or systematic search.

We also searched existing narrative (or literature) reviews to refine our search strategy and to check references for studies that should be included. Existing reviews were identified through the searches of electronic databases (listed previously), the grey literature, and the Campbell Library.

Lastly, we conducted an advanced Google search using criteria similar to the main database search and screening all results rather than the first 20 pages of results as originally specified in the protocol. We did not search Listservs of professional organizations (e.g., AERA) because of resource constraints. Results from the grey literature searches are discussed in the Results section and documented in Appendix A.2.

##### Soliciting authors of TFA studies

Based on studies we retrieved from our cursory searches initiated during the protocol development stage, we developed an email list of 25 researchers who authored an effectiveness study on TFA. We then drew a random sample of five researchers. We sent each an email that briefly described the Campbell systematic review on TFA, and provided a bibliography of all effectiveness studies identified from our cursory literature search as to whether these studies were eligible for our review. We requested that study authors refer us to any effectiveness studies not in the bibliography and/or any authors not in the bibliography who may be aware of TFA studies that were not formally published.

The reason for soliciting a sample of authors rather than the population of authors was to test whether the solicitation yielded enough additional new studies to justify solicitinga population of authors. It did not. Therefore, we stopped with the sample, inferring that soliciting a larger number of authors would not enable us to identify additional studies beyond what we found from our searches. The sampling frame (or population) of authors, random sample, and author response to the solicitation are discussed in the Results section and presented in Appendix A.3 (including an example email).

Studies that used the value‐added approach to estimate the effect of TFA on student outcomes and met the review inclusion criteria but did not report the data needed to estimate the ATE of TFA on student outcomes were eligible for an author query. We emailed these authors to request this information. If the information was provided, the study was included in the review. Otherwise, the study was excluded from the review.

##### Hand searches of journals

Limited resources and personnel prevented us from conducting a comprehensive hand search of social science journals where TFA studies were previously published. Consistent with guidance from the Campbell Information Retrieval Policy Brief, the Table of Contents for journal issues from 2014 to the present were manually scanned, online, for the following five journals:

- *Education Policy Analysis Archives*
- *Journal of Policy Analysis and Management*
- *Economics of Education Review*
- *American Educational Research Journal*
- *Journal of Human Resources*

When a title in the Table of Contents indicated a study of TFA, we went to that study and did a page‐by‐page scan to assess its eligibility.

The five journals were selected based on a review of our bibliography of effectiveness studies that identified journals that had *previously published* effectiveness studies on TFA. This hand search was conducted in February 2016 toward the end of the review to compensate for any lag time between when articles are published and when articles are indexed in the bibliographic databases. The results from the hand searches are reported in the Results section and documented in Appendix A.4.

### Data collection and analysis

#### Selection of studies

Studies retrieved from literature searches were screened. The initial screen focused on the study's title and abstract; however, if these did not contain the necessary information to complete screening, then the full article was retrieved. A study was screened out as ineligible due to its topic, time frame, sample, geographic location, design, or outcome relevance according to specifications outlined in the protocol and in the Campbell study coding guide (SCG), adapted from the WWC SRG.^8^

Studies that passed the initial screening were eligible for “Stage 1: Preliminary Screening” and “Stage 2: Quality of Evidence Review.” The difference between the “initial” and “preliminary” screenings is that the first was based on the title and abstract, and the second was based on the full text.

##### Stage 1: Preliminary screening

Studies that passed the initial screen were reviewed on the following criteria:

■ **Topic area.** Does the study focus on content that meets the definition of the topic area?
■ **Focus.** Is the intervention a program, product, policy, or practice as defined by the study's topic area?
■ **General education.** Does article fit the target sample as laid out in the study design?
■ **Time.** Is the publication date in a target publication year?
■ **Age or grade range.** Does the study fit the age or grade range as specified in the review protocol?
■ **Location.** Does the study examine sample members in a location specified in the review protocol?
■ **Outcomes.** Does the study address at least one academic or cognitive outcome?
■ **Screening results.** Does the study meet the screening criteria for the topic?

To be eligible for Stage 2 review, a study had to meet each of these criteria. Eligible studies were then screened in regard to the following three other characteristics:^9^

■ **Design.** What type of design is used to conduct the study?
■ **Effectiveness.** Does the study examine the effect of an intervention?
■ **Comparison group.** Does the study use a comparison group?

##### Stage 2: Quality of evidence review

The Stage 2 screening documented and evaluated research designcharacteristics of the study in order to determine whether it had sufficient internal validity for inclusion in the meta‐analysis. The design criteria we used to evaluate studies in Stage 2 were as follows:^15^

■ **Group assignment.** How are the intervention and comparison groups formed?
■ **Confounds.** Is the study free of factors that are confounded with either group?
■ **Eligible outcomes.** Is there at least one relevant outcome that meets the review requirements?
■ **Low attrition.** Is there at least one outcome, sample, or time point with lowattrition at the cluster and sub‐cluster level?
■ **Baseline equivalence.** Is evidence of baseline equivalence provided for at least one analytic sample?
■ **Other validity issues.** Are there other data or analysis issues that can affect the internal validity of the study?

RCTs were evaluated for low attrition. If there was high attrition, the RCT was evaluated for baseline equivalence in the analysis sample. QEDs were evaluated for baseline equivalence in the analysis sample, but not lowattrition.^10^

Studies that passed the Stage 2 review qualified for the meta‐analysis. Whether a study was ultimately included in the meta‐analysis depended on whether it (a) reported a contrast that was relevant to the research question and (b) whether the contrast reported by that study was also reported by at least one other study that passed the Stage 2 review. This is because at least two studies with the same contrast were needed to conduct the meta‐analysis.

#### Data extraction and management

All coding and screening were conducted by coders who were graduate students that had completed coursework in quantitative research methods, and attended a series of training sessions on research design and evidence standards led by the review team Principal Investigator (PI). During the training, coders independently completed a coding guide using an RCT retrieved from the cursory search used to develop the protocol. The coders then came together to go over their coding guides as a group. The review team gave coders independent feedback on the first drafts of their coding guides. Coders used this feedback to revise their guides and to correct coding errors. Once coders reconciled their coding guides to 100% agreement with the Master SCG, coders were assigned studies to start the review process.

During the review process, each study was screened and coded by two coders. Coding disagreements were reconciled to 100% agreement in a conference with both coders. It was rare that both coders could not come to an agreement. In those rare instances when there was a disagreement, it was resolved by the review team member assigned as the reconciler. The results of the two reconciled coding guides were entered into a final Master SCG that was used as “inputs” for the meta‐analysis conducted with CMA 3.0. All members of the review team who led the coding effort (including reconciliation) were certified in the use of the WWC Evidence Standards for Group Design, Version 3.0.

As previously noted, two reviewers (or coders) extracted data from the articles independently by recording the methods, participant characteristics, intervention characteristics, and outcomes in the SCG. When information needed to compute effect sizes, assess attrition, or establish baseline equivalence was not reported, a query was sent to the first author of the study (see Appendix B for an example of the author query email). When an author query did not retrieve the requested data, the study was still reported but was not included in the final meta‐analysis.

When an individual on the review team was also an author of an included study, that person was recused from coding the study and from being involved in the reconciliation or decision as to whether the study was included or excluded from the review.^11^ Information critical for evaluating the quality of studies eligible for review is displayed in Appendix C, Table C.1. A list of excluded studies and the reasons for exclusion are reported in Appendix C, Table C.2.

#### Assessment of risk of bias in included studies

##### High attrition in RCTs

High attrition is a major threat to the validity of the causal inference for an RCT (WWC, 2014). When the combination of overall and differential attrition is high, it can disrupt the initial equating of groups achieved through randomization. This disruption can introduce pre‐intervention differences between groups that are, in turn, confounded with post‐intervention differences between groups. To objectively evaluate whether an RCT has high attrition, we applied the *liberal* attrition thresholds as described in the WWC *Procedures and Standards Handbook, Version 3.0* (WWC, 2014), summarized in a *WWC Standards Brief* on attrition (WWC, n.d.), and applied as study quality evaluation criteria in the study coding guide used for this review.^12^

For example, if an RCT reported overall attrition of 5%, it had to report differential attrition of less than 10.5% to qualify as having “low” attrition. When an RCT had attrition that exceeded the a priori established thresholds for the combination of overall and differential attrition, the study team reviewed the study for information to evaluate baseline equivalence, on a pre‐intervention measure of the outcome, between treatment and control groups in the analysis sample. Thus, for a high attrition RCT to be internally valid, it was required to meet the same baseline equivalence standard as a QED.

##### Baseline equivalence in QEDs or RCTs with high attrition

A major threat to the internal validity of a QED or an RCT with high attrition is a lack of baseline equivalence between the intervention and comparison groups on a pre‐intervention measure of the post‐intervention outcome. This is because the pre‐intervention differences between groups could be confounded with post‐intervention differences. Reviewers calculated baseline equivalence, using a modified version of the WWC Study Review Guide (SRG).^13^ This calculation was done with complete case data, for pretest and posttest, in the analysis sample.^14^ We used the WWC standardized effect size thresholds for determining whether a QED or an RCT with high attrition has groups that are baseline equivalent on a pre‐intervention measure of the outcome. Group differences less than or equal to |.05| standard deviations were considered equivalent. Differences greater than |.05| standard deviations but less than or equal to |.25| standard deviations were deemed non‐equivalent, but could satisfy the baseline equivalence requirement by using a pretest covariate adjustment in the analysis model. Differences greater than |.25| standard deviations were considered non‐equivalent, but could satisfy the requirement through a covariate adjustment in the analysis model. These thresholds were applied to analysis samples based on the level of analysis (i.e., student, teacher, or school).

#### Measures of treatment effect

Data for calculating the standardized mean difference between groups were required to be un‐imputed for pre‐intervention (or pretest) values in the analysis sample, analysis sample sizes, and within‐group standard deviations in the analysis sample. The *t*‐statistic (or *p*‐value) and analysis sample sizes for the groups compared could also be used. Comprehensive Meta‐Analysis (CMA) 3.0 software can handle a wide variety of effect size inputs for calculating the standardized mean difference for baseline equivalence in the analysis sample. Studies that used imputed pre‐intervention values failed the baseline equivalence criteria.

#### Unit of analysis issues

The unit of assignment for RCTs and unit of matching for QEDs was considered when evaluating (1) whether a RCT had high attrition and (2) whether an RCT with high attrition or a QED established baseline equivalence between the TFA group and comparison group on pre‐intervention characteristics in the analysis sample. Consistent with the WWC Evidence Standards for Group Design, Version 3.0, and Campbell Methods Policy Briefs, studies that estimated the effect of TFA on students were evaluated for high attrition, baseline equivalence, or both at the student level, even if matching and random assignment occurred at a high level, such as classroom or school. For example, if an RCT that randomly assigned schools was evaluated but the effects of the intervention were estimated at the student level, the RCT was evaluated for high attrition at both the school and student levels without double‐counting.^15^ As another example, if a QED study matched at the school level but made inferences about the impact of the intervention on students, baseline equivalence had to be established at both the school and student levels.

We used the methodology outlined in the Campbell Statistical Analysis Policy Brief and the WWC *Procedures and Standards Handbook, Version 3.0*, to deal with dependency in the data. When there were *multiple* comparisons on a “single” outcome or *multiple* outcomes on a “single” comparison, we included the average effect size in the meta‐analysis. The effect size was a weighted average calculated using CMA 3.0 software.

#### Dealing with missing data

How researchers handle missing data can affect impact estimates and corresponding causal inferences. Acceptable methods for handling missing data are described in the WWC *Procedures and Standards Handbook, Version 3.0*, in the section on appropriate missing data methods. These standards were used to evaluate whether researchers used appropriate methods.

For some studies, authors reported the information needed to assess high attrition in RCTs, baseline equivalence for QEDs, baseline equivalence for RCTs with high attrition, and impact estimates. For other studies, including those that used value‐added methods, authors did not report this information. When the latter was the case, we sent an author query to request this information. When authors could not provide requested data or did not respond, we excluded the study from reporting in the meta‐analysis.

Author queries typically requested information to (1) calculate attrition for RCTs, (2) calculate baseline equivalence for RCTs with high attrition or QEDs, or (3) calculate effect sizes for study‐eligible outcomes. Each author query was tailored to the type of data requested. A list of studies that required an author query and the results of these queries is presented in Appendix B, Table B.1. An example of an author query is presented in Appendix B, Figure B.1.

For value‐added studies that used a comparison group, we emailed the authors to request the data needed to calculate baseline equivalence and effect sizes in the analysis sample. For other value‐added studies, the purpose of the author query was to provide authors with the opportunity to report data so the study could be evaluated as a QED. When authors could not provide requested data or did not respond, we excluded the study from reporting in the meta‐analysis. A list of value‐added studies that required an author query and the results of these queriesis presented in Appendix B, Table B.1.

#### Assessment of heterogeneity

CMA 3.0 provided us with the capability to quantify the amount of homogeneity in the individual effect sizes that comprise the average effect size. In theory, this involves empirically distinguishing between variation in the individual effect sizes due to sampling error, and variation in the individual effects due to true differences among studies using two homogeneity statistics—I^2^ and Q. The former is preferred because it is not dependent on sample size and does not lead to inferential errors due to low statistical power. However, because a small number of studies were eligible for the meta‐analysis, these statistics were not reported because they are not valid and may even be misleading with so few studies.

#### Assessment of reporting biases

Primary studies were assessed for quality and risk of bias and were assigned a rating of “meets design quality standards with reservations” or “without reservations,” based on the criteria reported in Appendix F.

#### Data synthesis

The primary goal of the meta‐analysis was to address research questions 3 through 10^16^ by estimating the ATE of TFA on student academic outcomes and teacher outcomes, and by examining the extent to which these outcomes are moderated by study characteristics, including fidelity of implementation. A related methodological goal was to quantify the precision of these effects (95% confidence intervals), and evaluate whether the effect is real or due to chance, using the actual *p*‐value compared to the alpha level set at .05. Because the study is the unit of analysis, statistical conclusion validity depends on having a large enough sample of studies in the meta‐analysis. Statistically, at least two studies with the same contrast (T vs. C group) on the same outcome were needed for meta‐analysis (Cooper, 2010). More studies are needed for a moderator analysis (Borenstein, Hedges, Higgins, & Rothstein, 2009).

Individual study effects were synthesized statistically using CMA 3.0. The software allows for over 100 different data entry formats for effect‐size calculations. The choice of effect‐size computation depended on three key factors: (1) the measures of the outcome variable(s), (2) the designs of studies reviewed, and (3) the statistical analyses that have been reported.

For studies that met our inclusion criteria and evidence quality standard, we computed the standardized mean difference.

TFA effectiveness studies that reported continuous outcomes were summarized using standardized mean differences. Because different studies used different outcome measures (in the same outcome domain), the standardized mean difference was the most appropriate effect size to be used in the meta‐analysis because it transforms mean differences expressed as raw or scale score units into mean differences expressed in standard deviation units.

Using CMA 3.0, we converted all effect‐size indices to Hedges’ g, which is a standardized mean difference with a small sample size bias correction factor. Hedges’ g is unbiased for both small and large samples. When substantively feasible, effect sizes were averaged across studies by using an inverse variance weighting of the individual effect sizes to account for differences in sample sizes for individual studies. This weighting resulted in the individual effect sizes of larger *n* studies being given more weight in the combined effect size. We calculated this effect size using a fixed effects and random effects model, with the effect size from the former being the main statistical model providing a basis for comparison. For both random effects and fixed effects models, individual study effect sizes and average effect sizes across studies were reported with confidence intervals and corresponding *p*‐values using Forest Plots. However, the small number of studies in the meta‐analysis suggest that estimation of between‐study variance is unreliable, the fixed effects models is most appropriate, and a Knapp‐Hartung correction as originally proposed in the systematic review protocol was *not* needed.

Results from the main effect meta‐analysis are reported in the Results section, according to the research questions these analyses were designed to address. Results are reported using forest plots with study sample sizes, effect sizes, 95% confidence intervals, *p*‐values, tests of homogeneity, and model choice of fixed or random effects.

#### Subgroup analysis and investigation of heterogeneity

##### Moderator analysis for categorical study‐level variables

We planned a moderator analysis, as described in the Campbell systematic review protocol for TFA studies, to address research questions 7a (the main effect of TFA moderated by TFA candidate status), 7b (the main effect of TFA moderated by teacher certification status), and 8 (does the main effect of TFA differ by fidelity of implementation?). However, there were too few studies in the meta‐analysis sample to conduct this analysis.

##### Moderator analysis for continuous study‐level variables

We planned a moderator analysis, as described in the Campbell systematic review protocol for TFA studies, to address research question 7c (the main effect of TFA moderated by average years of teaching experience); however, there were too few studies eligible for the meta‐analysis to conduct this analysis.

#### Sensitivity analysis

##### Sensitivity analysis

We originally planned to test the robustness of the conclusions drawn from the statistical synthesis through a sensitivity analysis of publication source and influential studies (or outliers), using funnel plots to assess the relationships between effect size and study precision. However, for a given research question, there were at most two and sometimes no studies, so the sample was too small to evaluate the possibility of publication bias. The small number of studies also precluded us from conducting a “one‐study removed” meta‐analysis to examine whether the results are sensitive to the inclusion or exclusion of particular studies. In short, there were not enough studies to conduct the aforementioned sensitivity analysis for any of the research questions.

##### Publication bias

There were not enough studies in the analysis used to address each research question and compare mean effect sizes of studies retrieved from peer‐reviewed sources with mean effect sizes of studies retrieved from unpublished sources (e.g., dissertations, government reports, conference presentations). To evaluate whether the overall estimate of TFA's average effect is affected by publication bias, we planned to address three questions using methods recommended by Borenstein and colleagues (2009, pp. 277–291):

■ Is there evidence of bias?
■ Is it possible that the entire effect is an artifact of bias?
■ How much of an impact might the bias have?

However, after consulting with our Technical Advisor (Michael Borenstein, personal communication, February 12, 2016), we concluded that it was neither possible nor sensible to test for publication bias when an analysis sample includes only two or three studies. Thus, the methods proposed in the protocol to address the three research questions—funnel plot, Rosenthal's fail‐safe N and Orwin's fail‐safe N, and Duval and Tweedle's Trim and Fill—were not used because the methods are not valid with a small number of studies.^17^

##### Treatment of qualitative research

To address research question 11, on the cost‐effectiveness of TFA, we reviewed studies included in the meta‐analysis for descriptive information on the cost or cost‐effectiveness of TFA. The purpose of this review was to identify what researchers found regarding TFA's cost‐effectiveness in order to provide context for study effect sizes (when such information was reported).

##### Group contrasts and study eligibility for meta‐analysis

Studies with at least one contrast that passed the Stage 2: Quality of Evidence Review were eligible for inclusion in the meta‐analysis. However, whether a study was actually included in the meta‐analysis depended on whether the contrast that passed Stage 2 was comparable to at least one other contrast that passed Stage 2 in another study. A *contrast* was defined as the comparison of a TFA group to a non‐TFA group on an eligible outcome for an eligible grade sample. For example, a single study may report a meta‐analysis‐eligible contrast that compared elementary‐grades students taught by TFA corps members (in their first or second year of teaching) to elementary‐grades students taught by non‐TFA novice teachers (in their first or second year of teaching) on math achievement. Whether this study was actually included in the meta‐analysis depended on whether another study reported the same contrast.

Defining contrasts is especially important in TFA research because of potential differences between teachers in the TFA group and teachers in the control (or comparison) group on such teacher characteristics as years of teaching experience and certification status. Controlling for these differences required that we meta‐analyze group contrasts from multiple studies that compared similar teachers in the TFA treatment group and non‐TFA group control (or comparison) group.

At least two studies that reported the same group contrasts were required to conduct the meta‐analysis. For example, if one RCT compared student outcomes of TFA corps members (in their first or second year of teaching) to student outcomes of non‐TFA novice teachers (also in their first or second year of teaching), but another study compared student outcomes of TFA alumni (who have completed their TFA commitment and have more than two years of teaching experience) to student outcomes of non‐TFA novice teachers (who have more than two years of teaching experience), then the two contrasts could not be meta‐analyzed. This is because both studies used control groups with the same type of non‐TFA teachers, but used treatment groups with different types of TFA teachers.

##### Use of evidence standards

This review used the WWC Evidence Standards for Group Design, Version 3.0. These standards are consistent with and not necessarily more rigorous than those used by Campbell. The WWC standards evolved from the Campbell standards that begin with Design, Implementation, and Assessment Device (DIAD) first developed in 2001 (Valentine & Cooper, 2008). The DIAD were the initial basis for the WWC standards, whose development was led by Campbell and the American Institutes for Research (AIR) under the first WWC contract (2002–2007). The relationship between the WWC standards and the Campbell and Cochrane criteria for bias are presented in Appendix F, Table F.1.

## Results

### Description of studies

#### Results of the search

The goal was to identify all studies on TFA's effectiveness that were published between 1994 and 2015 and were eligible for this review. What follows are the results from searching electronic databases, searching the grey literature, reaching out to a random sample of previous authors of TFA studies, and manually scanning the Table of Contents of the 2014 and 2015 issues of journals where TFA effectiveness studies were published previously.

#### Included studies

##### Electronic database searches

Table 3 reports results for the search terms that were cumulatively applied to each of the 11 databases we searched separately. For example, in the search of ERIC database, when Search 1 (program name terms) was used, 159 studies were retrieved. For the same database, when Search 1 was combined with Search 2 (academic outcomes terms), 99 studies were retrieved. Across the 11 databases, 173 citations were retrieved with the complete search strategy (i.e., Search Strategy = Search 1 + Search 2 + Search 3 + Search 4).

**Table 3:** Search and Screening Results from Electronic Databases

Electronic Database Searched	Search Strategy^1^				Initial Screening^2^		
	Search 1	Search 2	Search 3	Search 4	Passed	New	Duplicate^3^
Worldwide Political Science Abstracts	2	1	1	0	0	0	0
JSTOR	18	8	–^4^	4	1	0	1
Academic Search Premier	432	138	98	1	1	0	1
Education Full Text	408	172	139	4	4	0	4
Total	1,150	523	411	173	28	15	13

1 Search strategies were implemented cumulatively and with each database separately as described in the Methodology section and presented in Appendix A.1. That is, S2 = S1 + S2, S3 = S1 +S2 + S3, and so on.

2 We conducted relevance screening primarily based on the citation and abstract, and we screened the full text when necessary, using the criteria described previously in the Methodology section.

3 If the search retrieved the same citation retrieved in an earlier search of another database, the citation was labeled “duplicate.”

4 A dash (“–”) means that the search term could not be implemented with this database.

Table 3 shows that 69 citations were retrieved from ERIC using Search 4. Of these, eight citations referenced studies that passed the “initial” screening based on the title and abstract. Because the citations retrieved from ERIC were reviewed first, all eight referenced studies were new. As Table 3 also shows, 26 citations were retrieved from PsycINFO using Search 4. Of these, two citations referenced studies that passed the initial screening, but both were duplicates—they were retrieved previously using Search 4 with ERIC. Across the 11 databases, in the order that they were searched, 173 citations were retrieved with Search 4. Of these, 28 citations referenced studies that passed the initial screening: 15 citations referenced *new* studies, and 13 referenced studies that were *duplicates*.

The full text for the 15 citations (*n* = 15) that referenced eligible studies were retrieved. These studies were eligible for the Stage 1: Preliminary Screening and Stage 2: Quality of Evidence reviews conducted by two coders using the SCG categories and criteria therein. The complete search strategies and list of studies that passed initial screening, before Stage 1 and Stage 2 coding, are presented in Appendix A.1.

##### Grey literature search

The most citations were retrieved from the search of grey literature websites and databases. Of the 396 citations retrieved, 26 passed the initial screening and were eligible for Stage 1 and Stage 2 review. However, 18 of these citations were retrieved previously from the search of electronic databases, leaving 8 that were new citations eligible for review. The specific results of the grey literature search are documented in Appendix A.2.

##### Referrals, hand searches, and literature reviews

Referrals from a random sample of researchers did not produce any new studies. See Appendix A.3 for sampling details. Our hand search of five journals available online identified only one new study (see Appendix A.4). Finally, we identified one new study from 110 citations reviewed in the References sections of the following three literature reviews:^18^

■ Heilig, J. V., & Jez, S. J. (2010). *Teach For America: A Review of the Evidence*. (*n* = 56)
■ Seftor, N., & Mayer, D. P. (2003, March 31). *The Effect of Alternative Certification on Student Achievement: A Literature Review*. (*n* = 12)
■ Teach For America. (n.d.). *What the Research Says*. (*n* = 42)

Table 4 presents a summary of the results from our searches of citations beyond electronic databases.

**Table 4:** Citations Retrieved from Referrals, Hand Searches, and Literature Reviews

Source	Citations	Eligible	Duplicate	New
Referrals	4	4	4	0
Hand Searches	17	1	0	1
Literature Reviews	110	–	–	0
Total	131	5	4	1

##### Summary of search results

Table 5 summarizes the results of our searches across all sources. In all, we retrieved 919 citations on TFA using our search strategies. Of these, 61 passed the initial screening (based on study title and abstract), 34 were duplicates, and 27 studies were new and therefore eligible for Stage 1 and Stage 2 reviews by two coders, using the SCG, coding categories, and coding criteria described previously.

**Table 5:** Results from All Sources Searched

Source	Citations	Screening Result		
		Initial	Duplicate	New
Bibliographic Databases	173	28	13	15
Grey Lit Websites and Databases	396	26	18	8
Referrals from Researchers	4	4	4	0
Hand Searches	17	1	0	1
Literature Reviews	110	1	1	0
Total	700	60	36	24

##### Stage 1 and Stage 2 coding results

The twenty‐four studies were coded in two stages. In stage 1, studies were documented and evaluated for their relevance according to the criteria described previously in the methods sections. In stage 2, studies were evaluated and documented for the quality of the evidence according to the criteria described previously in the methods section.

###### Design characteristics of reviewed studies

According to Table 6, there were five design types used in the 24 studies that were initially reviewed for inclusion in this systematic review. Of these designs, QEDs^19^ (41%) were most frequently used, followed by RCTs (21%) and observational studies that used value‐added approaches (25%). Correlation studies (8%) were the next most prevalent design. There was one RDD used in the study sample. In all, 62% (*n* = 15) of studies used designs that were eligible for this review (QEDs and RCTs).

**Table 6:** Designs of Studies Reviewed for Inclusion in the Meta‐Analysis

Study Design	Design Acronym	*n*	Percent	Failed Review		Meta‐Analysis
				Stage 1	Stage 2	
Quasi‐Experiment	QED^1^	11	46%	2	8	1
Experiment	RCT^1^	4	17%	0	1	3
Observational: Value‐Added	OBSVA^2^	6	25%	0	6	0
Correlational	COR	2	8%	2	0	0
Regression Discontinuity Design	RDD	1	4%	1	0	0
Total	‐	24	100%	5	15	4

1 RCTs and QEDs were eligible for the meta‐analysis provided that they passed the other design criteria (summarized in Table 7). Primary fail reason for QEDs was insufficient data to establish baseline equivalence of TFA and non‐TFA groups in the analysis sample.

2 OBSVA studies use value‐added estimates to determine TFA's effect. Six authors (one for each “valued added” study) were sent an author query to ask for data to calculate attrition, baseline equivalence, and effect sizes (for ATE). Two responded that the data were not available, and four did not respond.

Table 6 also shows that only 17% (*n* = 4) of the 24 eligible studies qualified for meta‐analysis. To qualify for the meta‐analysis, a study needed to have at least one contrast that “Met Design Quality Standards” *with or without reservations* (as described in the Methodology section). Of the four studies that passed Stage 1 and Stage 2 review and qualified for the meta‐analysis, three used an RCT design and one used a QED. (See Table C.1 in Appendix C for specific studies, Stage 1 and Stage 2 review status, and their eligibility for meta‐analysis.)

##### Design and methodological characteristics of included studies

Table 7 reports information that answers the first research question posed in this systematic review:

1. What are the study characteristics of RCTs and QEDs conducted on TFA that met our inclusion criteria and were reported in this systematic review?

**Table 7:** Methodological Characteristics of Studies that Qualified for the Meta‐Analysis

Study	Research Design Quality Standards					Main Effect Contrast
	Design	Confound	High Attrition	Baseline Equiv.	R&V^1^ Outcome	
Decker et al., 2004^2^	RCT	No	No	NA	Yes	TFA vs. Non‐TFA
Turner, Goodman, Adachi, Brite, and Decker, 2012, ^3^	QED	No	NA	Yes	Yes	TFA_C vs. Non‐TFA_N; TFA_A vs. Non‐TFA_V^4^
Clark et al., 2013^5^	RCT	No	No	NA	Yes	TFA vs. Non‐TFA
Clark et al., 2015^6^	RCT‐Scale Up	No	No	NA	Yes	TFA_C vs. Non‐TFA; TFA_C vs. Non‐TFA_N

1 R&V = reliable and valid.

2 The Decker, Mayer, and Glazerman (2004) report was used because it was more detailed than the Glazerman, Mayer, and Decker (2006) journal article; however, both reports were used for coding the study. Decker, Mayer, and Glazerman (2004) used the Iowa Test of Basic Skills.

3 Turner et al. (2012) used the Texas Assessment of Knowledge and Skills.

4 TFA_C = corps members, Non‐TFA_N = novice teachers, TFA_A = alumni, and Non‐TFA_V = veteran teachers.

5 Clark (2013) used end‐of‐course standardized math assessments developed by the Northwest Evaluation Association

6 Clark et al. (2015) used the Woodcock‐Johnson III Achievement test for pre‐K through grad 2 and state assessments for grades 3–5. All of these standardized assessments were “assumed” to be reliable and valid.

To qualify, *methodologically*, for the meta‐analysis, studies had to pass the Stage 2: Quality of Evidence review (summarized in Table 7). To be included in the meta‐analysis, a study had to report at least one contrast that was the same contrast used by least one other study, so that the contrast could be synthesized (or averaged).

As Table 7 shows, three RCTs that used randomization to form TFA and non‐TFA student groups. These studies also passed the four applicable quality of evidence criteria: (1) The design was a valid RCT based on the description of the randomization, (2) there were no discernable confounds, (3) attrition was low, and (4) the math and reading outcomes were measured with standardized assessments that were reliable and valid. The baseline equivalence criteria did not apply because attrition was low (based on our calculations using the SCG). (See Appendix C for a list of excluded studies and the reasons for their exclusion.) As Table 7 also shows, only one QED (out of the original seven) passed the four applicable quality of evidence criteria: (1) A counterfactual group was shown to be baseline equivalent with the TFA group in the analysis sample, (2) there were no discernable confounds, (3) baseline equivalence was established at the student level, and (4) the outcomes were measured with the Texas Assessment of Knowledge and Skills, which is a reliable and valid standardized assessment.

Decker and colleagues (2004) examined the main effect of TFA on student reading and math outcomes, while Clark and colleagues (2013) examined the main effect of TFA on student math outcomes by contrasting the TFA (corps members and alumni) group and the Non‐TFA (novice and veteran teachers) group. The scale‐up RCT conducted by Clark and colleagues (2015) examined the main effect of TFA on student reading and math outcomes by contrasting four groups:

■ TFA_C (corps members) vs. Non‐TFA (novice and veteran teachers)
■ TFA_C (corps members) vs. Non‐TFA_N (novice teachers)

Turner and colleagues (2012) examined the effects of TFA on student reading and math outcomes by contrasting four other groups:

■ TFA_C (corps members) vs. Non‐TFA_N (novice teachers)
■ TFA_A (alumni) vs. Non‐TFA_V (veteran teachers)

In these studies, TFA corps members were in their first or second year of teaching, whereas TFA alumni had completed their contract and had three or more years of teaching experience. These two TFA groups corresponded to the two non‐TFA teacher groups: (1) non‐TFA novice teachers in their first or second year of teaching, and (2) non‐TFA veteran teachers with three or more years of teaching experience.

Although both Clark and colleagues (2015) and Decker and colleagues (2004) examined the effects of TFA on student math and reading outcomes in the elementary school grades, the results (for main effects) could not be synthesized in a meta‐analysis because the respective studies examined different contrasts.^20^ To combine these (or other non‐similar) contrasts would expose the meta‐analysis to the criticism of combining “apples” and “oranges” (Glass, 1976; Kizilirmak, Ozdemir, & Ongen, 2015). For this reason, the Clark and colleagues (2015) study *qualified* for the meta‐analysis methodologically, but was *excluded* from it due to lack of contrasts in common with other studies.

There were two methodological caveats for the QED study conducted by Turner and colleagues (2012) that the reader should consider when interpreting the effect sizes from this study in the meta‐analysis. First, because of confidentiality concerns by the Texas Education Agency (TEA) that provided the data, the researchers relied on TEA to create the teacher‐to‐student link, and the accuracy could not be verified. Second, the researchers also reliedon TEA to create the teacher “years of experience” variable, and the accuracy of this variable could not be verified.^21^

##### Sample characteristics of studies that qualified for the meta‐analysis

Table 8 reports information that answers the second question posed in this systematic review:

2. What are the sample characteristics of the schools, teachers, and students on RCTs and QEDs that met our inclusion criteria and were reported in this systematic review?

**Table 8:** Sample Characteristics of Studies that Qualified for the Meta‐Analysis

Study	Grade	Teachers’ Experience		Students FRL^1^ Eligible	TFA Implementation
		T^3^	C	Overall	Sites
Decker et al., 2004^2^	Elem.	2.0	6.0	95.3	Calif., Ill., La., Md., Miss., Texas
Turner et al., 2012	Elem., Middle	NR	NR	87.6^4^	Texas
Clark et al., 2013	Middle, High	1.9	10.1	90.2^4^	8 states
Clark et al., 2015	Elem.	1.7	13.7	83.7	10 states

FRL = free or reduced‐price lunch.

Three studies included elementary schools; one study included elementary and middle schools, and one study included middle and high schools (see Table 8). On average, across studies that reported average years of teaching experience, the TFA group had just under 2 years of experience, and the control or comparison group had approximately 10 years of experience. Consistent with TFA's mission, on average, 90% of students were eligible for free or reduced‐price lunch. Finally, except for the Turner and colleague's 2012 study, TFA implementation sites spanned multiple states.

#### Excluded studies

Study citations and reasons for exclusion are detailed in Appendix D.

### Risk of bias in included studies

As described in the previous section on “Use of evidence standards,” primary studies were assessed for quality and risk of bias using the WWC Evidence Standards for Group Designs, Version 3.0. These standards align with the Campbell risks of bias because both the “standards” and “risk” of bias evolved from the Design, Implementation, and Assessment Device (DIAD) first developed in 2001, used as the foundation for the WWC Evidence Standards for Group Design 1.0, and published by Valentine and Cooper in 2008. In sum, studies (or specific contrasts within studies) that were assigned a rating of “meets design quality standards with reservations” or “meets design quality standards with reservations by were assessed for risk of biasusing WWC Evidence Standards for Group Designs Version 3.0 that aligned with the risk of bias criteria presented in presented in Appendix F, Table F.1., had minimum risk, and as a consequent were included in the systematic review.

### Synthesis of results

#### Meta‐analysis results

The quality of a meta‐analysis can be only as good as the quality of the studies that are included (Hunt, 1997; Light & Pillemer, 1984). This includes comparing similar groups in the treatment versus control contrast. For TFA studies, this means comparing teachers in the TFA group with similar teachers in the comparison group. This review revealed that such comparisons were the exception rather than the rule.

##### Effects of TFA on student academic outcomes

Although four studies qualified for the meta‐analysis, research questions 3–5 could not be answered because no two studies estimated the main effect of TFA using TFA corps members *only* in the treatment group:

3. What are the main effects of TFA corps members on elementary school students in math, ELA, or science outcomes by research design?
4. What are the main effects of TFA corps members on middle school students in math, ELA, or science outcomes by research design?
5. What are the main effects of TFA corps members on high school students in math, ELA, or science outcomes by research design?

Further, even though the RCT conducted by Clark and colleagues (2013) estimated the main effect of TFA using corps members only in the treatment group, this was done at a different grade level—middle and high school—than the grade levels of the other two RCTs (elementary school).

**Figure 3:**
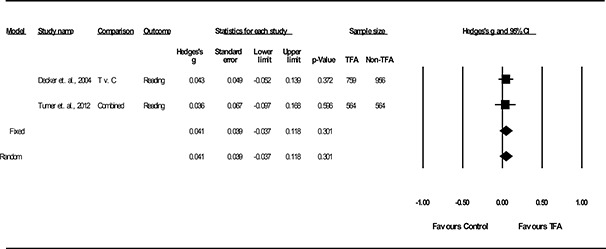
Main effect of TFA corps members and alumni on elementary‐grades students’ reading achievement. Note: The treatment group (T) is TFA corps members and alumni combined. The term “Combined” in the “Comparison Column” refers to the averaging of the two contrasts (TFA corps vs. non‐TFA novices, and TFA alumni vs. non‐TFA veterans) in the Turner et al. (2012) study.

**Figure 4:**
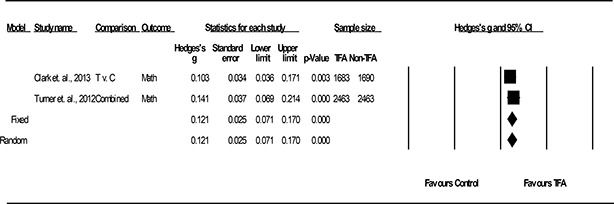
Main effect of TFA corps members and alumni on middle‐grades students’ math achievement. Note: Effect‐size differences between the two studies (RCT and QED) were less than .05 std. “Combined” refers to the averaging of the two contrasts (TFA corps vs. non‐TFA novices and TFA alumni vs. non‐TFA veterans) in the Turner and colleagues (2012) study.

Figures 3 and 4 report information pertaining to research question 6:

6. Are the main effects estimated by research design similar enough to be combined? If so, what is the combined main effect of TFA at each grade level and corresponding outcome?

**Elementary‐grades students’ reading achievement.** As Figure 3 shows, the standardized mean difference between the main effect of TFA on elementary school students’ reading achievement in the Decker and colleagues (2004) RCT and the Turner and colleagues (2012) QED was less than .05 standard deviations (std). This standarized difference met the empirical criteria for combining an RCT and a QED, as established in the protocol and based on WWC baseline equivalence standards. Thus, it was appropriate to estimate the combined effect of 0.041 std using the two different designs. In addition, each study used TFA corps members and TFA alumni (i.e., “TFA All”) in the treatment group and compared them to all “new” and “veteran” teachers in the control or comparison group (i.e., “non‐TFA All”).^22^ In other words, each study contrasted “TFA All” vs. “non‐TFA All.”

The Forest plot in Figure 3 summarizes the meta‐analysis results for the fixed effects model^23^ as follows:

■ Across the two studies, there were 1,323 students in the TFA group and 1,520 students in the non‐TFA group, for a total sample size of 2,843. For the combined samples, there was a small, positive, but not statistically significant main effect of TFA on reading achievement (ES = .041, *p* = .301).
■ There is a 95% confidence level that the true “main” effect of TFA on reading achievement ranges from ES = –0.037 to 0.118 std. Because our confidence interval includes zero, we concluded that there is no discernible effect of TFA corps members and alumni versus “new” and “veteran” non‐TFA teachers on reading achievement, given the outcome variable and samples used in these studies.

**Elementary‐grades students’ math achievement.** The difference between the RCT study conducted by Decker and colleagues (2004) and the QED study conducted by Turner and colleagues (2012) on the main effect of the math outcome was 0.09 std, which exceeded the .05 std threshold, and therefore the studies were too different to meta‐analyze to estimate an overall effect size. Specifically, the Decker and colleagues (2004) study effect size (ES) was 0.15 std, and the Turner and colleagues (2012) study ES was 0.06 std.

**Middle‐grades students’ math achievement.** The differences between the main effect of TFA on middle‐grades students’ math achievement in the Clark and colleagues (2013) RCT and the Turner and colleagues (2012) QED was less than .05 std. This difference met the empirical criteria for combining an RCT and a QED, as established in the protocol and based on WWC baseline equivalence standards. Thus, it was appropriate to estimate the combined effect of 0.121 std using the two different designs. In addition, each study used TFA corps members and TFA alumni (i.e., “TFA All”) in the treatment group and compared them to all “new” and “veteran” teachers in the control or comparison group (i.e., “non‐TFA All”). In other words, each study contrasted “TFA All” vs. “non‐TFA All.”

The forest plot in Figure 4 summarizes the meta‐analysis results for the fixed effects model as follows:

■ Across the two studies, there were 4,146 students in the TFA group and 4,153 students in the non‐TFA group, for a total sample size of 8,299. For the combined samples, there was a moderate, positive, and statistically significant main effect of TFA on math achievement (ES = 0.121, *p*<.001).
■ We are 95% confident that the true “main” effect of TFA on math achievement ranges from ES = 0.071 to 0.170 std. Because our confidence interval excludes zero, we conclude that the detected effect of TFA on math achievement is beyond what one would expect by chance, and of a magnitude that is positive.

##### Effects of TFA, moderators, mediators, and costs

There were too few studies to address research questions 7 and 8:

7. How do the magnitude and statistical significance of the main effect of TFA change when controlling for the following teacher characteristics separately, in a moderator analysis?
  - TFA candidate status (e.g., corps member or alumnus)
  - Teacher certification status (e.g., traditionally certified, alternatively certified, or not certified)
  - Teacher average years of teaching experience
8. Is there sufficient fidelity of implementation information reported in TFA studies? If so, to what extent does the main effect of TFA differ by fidelity of TFA implementation?

It was not possible to conduct a valid moderator analysis to evaluate whether the main effect of TFA was moderated by TFA candidate status, teacher certification status, or such teacher characteristics as average years of teaching experience. Nor was it possible to conduct a valid moderator analysis to evaluate whether the main effects of TFA differed by study characteristics, such as fidelity of TFA implementation.

Insufficient information in the studies reviewed also precluded us from answering research questions 9 and 10:

9. Is there sufficient information on teacher turnover in TFA studies to evaluate TFA's main effect on teacher retention? If so, what is the main effect on teacher retention?
10. Is there sufficient information on teacher leadership, content knowledge, years of teaching experience, or overall academic ability to evaluate TFA's main effect on teacher quality? If so, what is the main effect on teacher quality?

For example, regarding teacher turnover, none of the four studies that methodologically qualified for the meta‐analysis reported teacher turnover in both the treatment and control groups. The other 11 studies that did not methodologically qualify for the meta‐analysis of the effect of TFA on student academic outcomes were reviewed to determine if they qualified for the meta‐analysis of the effect of TFA on teacher turnover. Only one of these studies (Ready, 2014) focused on teacher turnover. This study, however, did not report information to establish baseline equivalence in the analysis sample and could not be used to address the teacher turnover question.

Similarly, regarding teacher quality, neither studies that methodologically qualified for the meta‐analysis nor studies that did not methodologically qualify for the meta‐analysis—but were reviewed for the meta‐analysis on teacher quality—reported the relevant teacher outcomes in both treatment and control groups to address research question 10.

Insufficient information from the review of the eligible studies also precluded us from answering research question 11:

11. Is there sufficient cost information in TFA studies to evaluate whether the literature reports TFA as cost‐effective? If so, is TFA reported to be cost‐effective?

None of the studies included in the meta‐analysis provided sufficient cost information to evaluate whether TFA is reported as cost‐effective.

## Discussion

As TFA celebrates its 25th anniversary, this systematic review is relevant for four reasons: (1) It takes stock of the quality of experimental and quasi‐experimental evidence used to estimate the average effects of TFA on student academic outcomes, (2) it identifies gaps in research knowledge about these effects, (3) it draws conclusions, with implications for policy, and (4) it makes recommendations for future research.

### Summary of main results

This review applied systematic review methods to limit bias in the retrieval, appraisal, and statistical synthesis of findings from primary studies on the effects of TFA on academic outcomes (Cooper, 2010; Petticrew & Roberts, 2006). As a central tenet of a Campbell systematic review is transparency, throughout this review the methods used were carefully reported and documented. Readers may agree or disagree with our methods and corresponding decisions, but transparency demands that they know exactly what methods were used and what decisions were made, including why particular studies were included in or excluded from the meta‐analysis.

We identified 700 citations from bibliographic databases, grey literature websites, grey literature databases, hand searches, and references in previously conducted literature reviews (see Table 5). We also requested referrals from a small, random sample of researchers (see Table 5). Together, these information‐retrieval activities comprised the most comprehensive search for studies on TFA to date.

Successive application of search term filters resulted in 24 studies eligible for review after excluding duplicates (see Table 5). Of these, only four met the evidence criteria for inclusion in the meta‐analysis, with three using an RCT design and one using a QED. The three RCTs reported proper implementation of random assignment, exhibited low attrition, used reliable and valid outcomes, and had no confounds. The QED used matching at the school and student levels, established baseline equivalence in the analysis sample at both levels, used reliable and valid outcomes, and had no additional confounds beyond unmeasured characteristics endemic to any QED.

There is no significant effect on reading from teaching by TFA corps members in their first or second year of teaching elementary‐grade students (PreK – grade 5) compared to non‐TFA

teachers who are also in their first or second year of teaching elementary‐grade students. There is a small positive for early elementary‐grade students (PreK to grade 2) in reading but not in math. However, given the small evidence base these findings should be treated with caution.

### Overall completeness and applicability of evidence

Approximately 33% of the studies reviewed were not eligible for the meta‐analysis because they used designs that were not RCT or QED. Ineligible designs included teacher value‐added studies that did report, or could not provide through an author query, data to estimate average treatment effects. We conclude that when research design and study quality are considered, the evidence base for estimating the ATE of TFA on student academic outcomes is small (*n* = 4). This small evidence base limited the meta‐analysis and the questions on TFA that could be addressed in this systematic review, and the extent to which the findings generalize to all studies that were eligible for the meta‐analysis.

### Quality of the evidence

Only 17% of the eligible studies (4 out of 24) met the evidence criteria for inclusion in the meta‐analysis. The three RCTs reported proper implementation of random assignment, exhibited low attrition, used reliable and valid outcomes, and had no confounds. As a result, the study effect sizes and synthesis of them is unbiased and there were no reservations about this lack of bias. The QED used matching at the school and student levels, established baseline equivalence in the analysis sample at both levels, used reliable and valid outcomes, and had no additional confounds beyond unmeasured characteristics endemic to any QED. However, these unmeasured characteristics are the reason for the reservations about whether the reported effect size can be attributed to TFA solely.

### Limitations and potential biases in the review process

Future systematic reviews and meta‐analyses can improve on this systematic review and meta‐analysis by addressing the following limitations:

■ The meta‐analysis results for middle school math and for elementary school reading were each based on two studies. To put this number in perspective, the median number of studies in a Cochrane review is three.
■ The information‐retrieval phase of this review was comprehensive. However, there were too few studies to test for publication bias. At least 10 studies are needed for a reliable and valid test (Borenstein, Hedges, Higgins, & Rothstein, 2009). This limitation also applied to other publication bias tests, such as the funnel plot, Trim and Fill method, and fail‐safe N method.
■ The small number of studies in each meta‐analysis means that the results could be highly sensitive to studies that were excluded because of lack of information to evaluate their eligibility, or the results could be highly sensitive to new studies that will be identified in a future systematic review.

#### Gaps in knowledge on the ATE of TFA

The 11 research questions posed in this review were crafted carefully during the protocol development process. They are considered the most important to answer to contribute to knowledge about the ATE of TFA on student academic outcomes. The eight questions that could not be answered using meta‐analysis are evidence that after 25 years of TFA's existence, there remain substantial gaps in knowledge on the effects of TFA. These gaps have important implications for future policy and research related to TFA. There is an urgent need to craft a TFA research agenda that lays the foundation for the next 25 years that is more coherent and conducive to a systematic review and meta‐analysis.

### Agreements and disagreements with other studies or reviews

In previous research, individual QED studies on TFA identified mixed effects of TFA (positive or null) on student math achievement. RCT studies, on the other hand, identified positive and statistically significant effects of TFA on student math achievement, although these findings varied by grade level (Clark et al., 2013; Clark et al., 2015; Glazerman et al., 2006; Heilig & Jez, 2010). Both QEDs and RCTs pointed to no discernable effects of TFA on reading achievement at every grade level.

A key qualitative finding from this review is that studies eligible for the meta‐analysis varied widely in how they defined the TFA group and how they defined the corresponding control or comparison group (see Table E.1 in Appendix E). This variation limited what research questions could be addressed using meta‐analysis. For example, the first RCT on TFA reported by Decker and colleagues (2004) and the scale‐up RCT reported 11 years later by Clark and colleagues (2015) were both eligible for the meta‐analysis; however, they could not be meta‐analyzed because the two studies did not share comparable TFA treatment groups.

Based on the two studies included in the two meta‐analyses, when TFA corps members and alumni in the treatment group were compared to all non‐TFA teachers using a fixed effects model to estimate the combined effect size across the two studies, we found the following:

■ There was a 95% confidence level that the effects of TFA on elementary school reading is between –0.037 and 0.118 std (ES = 0.041), and this effect is not statistically significant (*p* = 0.301). This estimate is based on a total sample of 2,843 students in five states, including Texas (which was common to both studies).
■ There was a 95% confidence level that the effects of TFA on middle school math is between 0.071 and 0.170 std (ES = 0.121) and is statistically significant (*p*< .0001). This estimate is based on a total sample of 8,299 students in nine states.

Thus, from a meta‐analytic perspective, we conclude that TFA corps members and alumni together have a positive and statistically significant effect on math achievement for middle school students, but no discernable effect on reading achievement for elementary school students. Although it is tempting to compare this finding with those from previous research, doing so would undermine the most important qualitative finding from this review:

When describing the effects of TFA and comparing these effects to other TFA research, it is critically important to define exactly who is in the TFA group (corps member, alumni, or both), and who the TFA group is being compared to (new teachers, veteran teachers, certified teachers, non‐certified teachers, or some combination of all four).

This review did not identify enough evidence to draw meta‐analytic conclusions about the effects of TFA corps members only, or the effects of TFA alumni members only, relative to non‐TFA teachers at any grade level or for any academic outcome that was the focus of this review. The small number of eligible studies and limited number of contrasts that were common across studies resulted in our addressing only 3 of the 11 research questions posed in this review. From a meta‐analytic point of the few, the small evidence base severely constrained what we learned and what we can report about the ATE of TFA on student academic outcomes at the elementary, middle, and high school levels.

## Authors’ conclusions

### Implications for practice and policy

When trying to discern the effects of TFA on student academic achievement to make policy decisions, state, district, and school leaders should consider the following evidentiary facts, based on the results of this systematic review and meta‐analysis:

■ Although this review identified 5 RCTs and 10 QEDs that were eligible for review, only 4 studies (3 RCTs and 1 QED) exhibited the methodological qualities to be eligible for this meta‐analysis.
■ When a study, literature review, or systematic review presents results on the average effects of TFA on student academic outcomes, it is critical to discern the composition of the TFA group and the composition of the comparison (or control) group that are contrasted before any conclusion can be drawn. The average effect can differ by contrast, and some contrasts may be included in one study but not the other.
■ There is evidence of a moderate, positive, and statistically significant effect of TFA corps members and alumni, combined, on middle‐grades students’ math achievement. This effect occurs when students of TFA corps members and alumni are compared to students of all types of non‐TFA teachers combined.
■ There is evidence of a small, positive, but *not* statistically significant effect of TFA corps members and alumni, combined, on elementary‐grades students’ reading achievement. This effect occurs when students of TFA corps members and alumni are compared to students of all types of non‐TFA teachers combined.

Nevertheless, given the small number of studies eligible for meta‐analysis, many unanswered questions remain about the average effects of TFA on student academic outcomes in the United States. Due to the lack of evidence, these questions cannot be answered with a level of confidence.

### Implications for research

Twice as many QEDs (*n* = 10) as RCTs (*n* = 5) were eligible for review. This ratio was not surprising. Random assignment studies to evaluate the effects of TFA on student academic outcomes can be extremely challenging to implement within schools. This is because students must be randomly assigned to teachers (to control for potential teacher confounds), and this can, among other things, be disruptive to school preferences for assigning students to teachers. This and other challenges with random assignment may explain, at least in part, why the Clark and colleagues (2015) scale‐up RCT was limited to elementary schools. Moreover, during the past 25 years, researchers have conducted only one RCT at the middle and high school levels (Clark et al., 2013). Although few, these RCTs have been well‐designed, well‐implemented, and well‐reported. However, we have two recommendations for improving the design of future RCTs:

■ Future RCT research should pay closer attention to, and strike a balance between, developing comparisons on outcomes that address the research questions for the study and aligning those comparisons with previous research so that future studies can be included in future systematic reviews and meta‐analyses related to TFA.
■ Specifically, the Clark and colleagues (2015) scale‐up RCT and the Turner and colleagues (2012) QED can serve as reference points. Appendix E presents all contrasts for all included studies to allow the reader to decide what contrasts are relevant when designing future studies.

In contrast to the RCTs reviewed, there is substantial room for improvement in the design, implementation, and reporting of QEDs, which is an important qualitative finding from this review. The challenges of conducting RCTs on TFA with random groups of students may drive researchers to continue to use the QED more frequently than the RCT design. If this proves true, the reader should be aware that the primary reason QED studies were excluded from the meta‐analysis was that they lacked information to evaluate whether TFA and non‐TFA student groups were equivalent, in the analysis sample, before the start of the TFA study year. This is an important methodological requirement, because it ensures that average differences between groups on the posttest are not confounded with pre‐existing differences between groups on a pretest (or its proxy). Further, because most QEDs failed the baseline equivalence requirement due to insufficient information, this was as much a reporting issue as a methodological one. It is important for researchers to understand that when establishing baseline equivalence for QED studies on TFA, this equivalence mustbe established without imputing data on the posttest and pretest in the analysis sample.

When designing QED studies, we recommend that researchers pay close attention to the following:

■ Establishing baseline equivalence in the analysis sample between treatment and comparison groups, and reporting this information as part of the results
■ Establishing this equivalence using non‐imputed data

Based on the results of the meta‐analysis, when deciding between estimating teacher value added or estimating the ATE (using RCTs or QEDs) on student academic outcomes, we recommend that researchers choose the latter—at least until methodological concerns about teacher value‐added reliability and validity (Andrabi et al., 2009) are addressed, and reliable and valid meta‐analytic methods are developed. Until then, we doubt that the use of teacher value added will “add value” to the meta‐analysis of the ATE of TFA on student academic outcomes.

The Clark and colleagues (2015) scale‐up study qualified methodologically for the meta‐analysis but was excluded because it did not share any contrasts in common with other studies that qualified for the meta‐analysis. However, readers should be aware of two things:

■ When TFA corps members in their first or second year of teaching were compared to non‐TFA teachers in their first or second year of teaching students in preK to grade 5, there was a positive but *not* statistically effect in reading (ES = 0.12, *n* = 313, *p*> .05) and math (ES = 0.03, *n* = 313, *p*> .05).
■ When TFA corps members in their first or second year of teaching were compared to *all* non‐TFA teachers of students in preK to grade 2, there was a positive and statistically significant effect in reading (ES = 0.10, *n* = 1,655, *p*< .05), but not in math (ES = 0.08, *n* = 1,655, *p*> .05).

## Recommendations for future research

Although TFA is the most evaluated program of its kind, and multiple quasi‐experimental and experimental studies have been conducted on the effectiveness of TFA in improving student outcomes, this systematic review and meta‐analysis of the effects of TFA clearly indicates that only a small number studies met the evidence review standards. We recommend that future intervention research on TFA focus on the following:

1. Using RCTs and QEDs with the potential to meet objective extant evidence standards
2. Studying TFA treatment groups and non‐TFA control or comparison groups that align with previous research reported in this first systematic review (including the Clark and colleagues [2015] scale‐up study)
3. Reporting study results and information so that the quality of evidence can be evaluated and effect sizes can be included in a systematic review and meta‐analysis
4. Following these recommendations can be a first step in ensuring that a future systematic review and meta‐analysis will be able to address the unanswered questions about TFA, based on the evidence.

## Information about this review

### Review authors

**Lead review author**

The lead author is the person who develops and co‐ordinates the review team, discusses and assigns roles for individual members of the review team, liaises with the editorial base and takes responsibility for the on‐going updates of the review.

**Table:** 

Name:	Herbert M. Turner, III
Title:	President and Principal Scientist / Adjunct Associate Professor
Affiliation:	ANALYTICA, Inc. / University of Pennsylvania
Address:	14 Pine Tree Drive
City, State, Province or County:	Methuen, MA
Post code:	01844
Country:	United States
Phone:	215.808.8880
Email:	herb@analytica-inc.com
**Co‐author(s)**	
**Name:**	
Title:	**Mackson Ncube**
Affiliation:	Scientific Researcher
Address:	14 Pine Tree Drive
City, State, Province or County:	Methuen, MA
Post code:	01844
Country:	United States
Phone:	215‐801‐3584
Mobile:	215‐801‐3584
Email:	mackson@analytica-inc.com
	
**Name:**	
Title:	**Annette Turner**
Affiliation:	Chief Executive Officer
Address:	ANALYTICA, Inc.
City, State, Province or County:	14 Pine Tree Drive
Post code:	Methuen, MA
Country:	01844
Phone:	United States
Mobile:	267‐885‐4570
Email:	annette@analytica-inc.com
	
**Name:**	**Robert F. Boruch**
Title:	University Trustee Chair, Professor of Education and Statistics
Affiliation:	University of Pennsylvania
Address:	3700 Walnut Street
City, State, Province or County:	Philadelphia, PA
Post code:	19104
Country:	United States
Phone:	215‐898‐0409
Email:	robertb@gse.upenn.edu
	
**Name:**	**Nneka Ibekwe**
Title:	Doctoral Student
Affiliation:	University of Pennsylvania
Address:	3700 Walnut Street
City, State, Province or County:	Philadelphia, PA
Post code:	19104
Country:	United States
Phone:	215‐898‐0409
Email:	nibekwe@gse.upenn.edu

### Roles and responsibilities

- Content: Dr. Herb Turner, Mr. Mackson Ncube, and Mrs. Annette Turner
- Systematic review methods: Dr. Herb Turner, Mr. Mackson Ncube, and Dr. Robert Boruch (Advisor: Dr. Michael Borenstein)
- Statistical analysis: Dr. Herb Turner and Mr. Mackson Ncube (Advisor: Dr. Michael Borenstein)
- Information retrieval: Dr. Herb Turner, Ms. Nneka Ibekwe

### Sources of support

Campbell Collaboration ECG Mini‐Grant from the Smith Richardson Foundation.

### Declarations of interest

Dr. Herb Turner was the PI of the Turner and colleagues 2012 study, and as such he was not included in the coding of this study or the decision to include the study in the meta‐analysis.

### Plans for updating the review

Dr. Turner will be responsible for updating the review.

## Online supplements

***List of online supplements***

1. APPENDIX A.1: Results from Electronic Database Searches
2. APPENDIX A.2: Results from Grey Literature Search
3. APPENDIX A.3: Study Referrals
4. APPENDIX A.4: Results from Hand Searches of Journals
5. APPENDIX B: Results of Author Queries
6. APPENDIX C: Coding Results for Review‐Eligible Studies (*n* = 24)
7. APPENDIX D: Excluded Studies and Reasons
8. APPENDIX E: Contrasts Reported in Included Studies
9. APPENDIX F: Risk of Bias

## Supporting information

Supplementary material

Supplementary material

## Footnotes

A meta‐analysis will not affect the statistical precision of individual primary study effect sizes (Becker and Cohn, 2003).

In the C2 protocol for this review, we reported that the scale‐up included middle and high school, but this was only true for the KIPP network's i3 scale‐up and not for the TFA i3 scale‐up.

Some studies reported results that included preK students. When this occurred, we alerted the reader when reporting these findings.

These definitions and criteria are needed to (a) calculate “attrition” for RCTs, (b) establish baseline equivalence for RCTs with high attrition and QEDs, and (c) estimate the ATE of TFA on teacher retention.

In the treatment group, this would be TFA corps members, TFA alumni, or both. In the comparison group, this would be non‐TFA teachers.

The order of sources searched is different from the orders of sources searched in the protocol because the former was, on reflection, a more logical progression of the search process than the latter.

See TFA protocol on the Campbell Collaboration website (http://www.campbellcollaboration.org/lib/project/344/).

We list the most salient additional characteristics here. See the SCG for the complete list of criteria.

This approach is consistent with WWC 3.0. The logic is that if attrition is non‐random or differential between groups in the QED, it will be reflected in a lack of baseline equivalence in the analysis sample.

The PI for this systematic review, Herb Turner, was an author of a reviewed study and was not involved in the coding or the decision to include or exclude the study.

The liberal standard is appropriate when the attrition is assumed to be exogenous (not related to the intervention) rather than endogenous (related to the intervention). However, for the contrasts included in the meta‐analysis, the results were not sensitive to selecting one standard or the other.

The modified coding guide did not change the WWC formulas for attrition, baseline equivalence, or effect‐size calculations.

For units of analysis with complete case data on pretest and posttest, we calculated the standardized mean difference between the treatment and control groups on the pretest (or pre‐intervention measure of the outcome).

This mean that student‐level attrition was calculated for only those clusters that remained in the study.

Question 11, regarding the cost‐effectiveness of TFA, is descriptive; for the studies included in the meta‐analysis, we simply noted what authors reported on TFA's cost effectiveness.

The curious reader should refer to the TFA Protocol for a Systematic Review in the Campbell Library (https://www.campbellcollaboration.org/library/download/504_9177bd28e56680accecb97706eb7bc42.html) for more information about these methods.

These reviews are cited in the References section.

Observation studies that used a comparison group were included in the QED category because if the group was baseline equivalent to the treatment group in the analysis sample (with or without covariate adjustment), the study could qualify for the meta‐analysis, provided that the other evidence criteria were met.

Specifically, Clark and colleagues (2015) did not report contrasts that combined TFA corps members with TFA alumni to be compared to a non‐TFA group (comprising novice and veteran teachers).

See Fuller and Dadey (2013) for their narrative review.

Turner and colleagues (2012) compared TFA corps members vs. non‐TFA novice teachers on reading achievement, and TFA alumni versus Non‐TFA veteran teachers on reading achievement. The contrasts were averaged in CMA3.0 to form the combined contrast of TFA all vs. non‐TFA all, which is comparable to the contrast in Decker and colleagues' (2004) study.

Results for the random effects model are presented for comparative purposes.

## References

[cl2014001035-bib-0001] Clark, M. A., Chiang, H. S., Silva, T., McConnell, S., Sonnenfeld, K., Erbe, A., & Puma, M. (2013). The effectiveness of secondary math teachers from Teach For America and the teaching fellows programs (NCEE 2013‐4016). Jessup, MD: National Center for Education Evaluation and Regional Assistance.

[cl2014001035-bib-0002] Clark, M. A., Isenberg, E., Liu, A. Y., Makowsky, L., & Zukiewicz, M. (2015). Impacts of the Teach For America Investing in Innovation scale‐up (Rep.). Princeton, NJ: Mathematica Policy Research.

[cl2014001035-bib-0003] Decker, P. T., Mayer, D. P., & Glazerman, S. (2004). The effects of Teach For America on students: Findings from a national evaluation (MPR Reference No.: 8792‐750). Princeton, NJ: Mathematica Policy Research.

[cl2014001035-bib-0004] Turner, H. M., Goodman, D., Adachi, E., Brite, J., & Decker, L. E. (2012, December). Evaluation of Teach For America in Texas schools. Retrieved from http://edvanceresearch.com/wp‐content/uploads/2015/06/Evaluation‐of‐Teach‐For‐America‐in‐Texas‐Schools.pdf

[cl2014001035-bib-0005] Antecol, H., Eren, O., & Ozbeklik, S. (2013). The effect of Teach for America on the distribution of student achievement in primary school: Evidence from a randomized experiment. Economics of Education Review 37, 113–125.

[cl2014001035-bib-0006] Bastian, K. C. (2014). Selecting and preparing teachers and school leaders to improve educational outcomes. Dissertation Abstracts International: The Humanities and Social Sciences.

[cl2014001035-bib-0007] Boyd, D., Grossman, P., Lankford, H., Loeb, S., & Wyckoff, J. (2006). How changes in entry requirements alter the teacher workforce and affect student achievement. Columbia, MO: American Education Finance Association. Retrieved from https://cepa.stanford.edu/sites/default/files/ReducingEntryRequirementsEPF2006.pdf

[cl2014001035-bib-0008] Boyd, D., Grossman, P., Hammerness, K., Lankford, H., Loed, S., Ronfeldt, M., & Wyckoff, J. (2012). Recruiting effective math teachers: Evidence from New York City. American Educational Research Journal, 49(6), 1008–1047.

[cl2014001035-bib-0009] Carroll, C. A. (2013). The influence of Teach for America on Algebra I student achievement. Unpublished doctoral dissertation, University of North Carolina, Charlotte.

[cl2014001035-bib-00010] Darling‐Hammond, L., Holtzman, D. J., & Gatlin, S. J. (2005). Does teacher preparation matter? Evidence about teacher certification, Teach for America, and teacher effectiveness. Education Policy Analysis Archives, 13(42), 1–47.

[cl2014001035-bib-00011] Dee, T. S., & Wyckoff, J. (2015). Incentives, selection, and teacher performance: Evidence from IMPACT. Journal of Policy Analysis and Management, 34(2), 267–297.

[cl2014001035-bib-00012] Evans, B. R. (2009). First year middle and high school teachers' mathematical content proficiency and attitudes: Alternative certification in the Teach for America (TFA) program. Journal of the National Association for Alternative Certification, 4(1), 3–17.

[cl2014001035-bib-00013] Evans, B. R. (2010). Determining quality teachers: Mathematical content knowledge, perceptions of teaching self‐efficacy, and attitudes toward mathematics among a Teach for America cohort. Journal of the National Association for Alternative Certification, 5(2), 23–35.

[cl2014001035-bib-00014] Henry, G. T., Thompson, C. L., Bastian, K. C., Fortner, K. C., Kershaw, D. C., Purrell, K. M., & Zulli, R. A. (2010, June). Portal report: Teacher preparation and student test scores in North Carolina. Retrieved from http://www.worldcat.org/title/portal‐report‐teacher‐preparation‐and‐student‐test‐scores‐in‐north‐carolina/oclc/789248583

[cl2014001035-bib-00015] Laczko‐Kerr, I. I. (2002). Teacher certification does matter: The effects of certification status on student achievement. Unpublished doctoral dissertation, Arizona State University, Tempe.

[cl2014001035-bib-00016] Noell, G. H., & Gansle, K. A. (2009). Teach For America teachers' contribution to student achievement in Louisiana in grades 4‐9: 2004‐2005 to 2006‐2007. Baton Rouge, LA: Louisiana Board of Regents.

[cl2014001035-bib-00017] Pearson, J. L. (2014). Effective instructional methods utilized in successful and high performing secondary schools in the Southern Region of Mississippi. Unpublished doctoral dissertation, The University of Southern Mississippi, Hattiesburg.

[cl2014001035-bib-00018] Penner, E. K. (2014). Teaching for all? Variation in the effects of Teach for America. Unpublished doctoral dissertation, University of California, Irvine.

[cl2014001035-bib-00019] Prescott, S. H. (2010). The effects of affirmative quality feedback on low socio‐economic students' zone of proximal development reading gains (ZPDRL): A causal‐comparative study. Unpublished doctoral dissertation, The University of Mississippi, Oxford.

[cl2014001035-bib-00020] Raymond, M., & Fletcher, S. (2002, Spring). The Teach for America Evaluation: Herewith, the first evidence on its recruits' actual performance in the classroom. Education Next, 62‐

[cl2014001035-bib-00021] Raymond, M., Fletcher, S., & Luque, J. (2001). Teach for America: An evaluation of teacher differences and student outcomes in Houston, Texas. Stanford, CA: The Center for Research on Education Outcomes.

[cl2014001035-bib-00022] Ready, D. D. (2014). Teach for America Teachers in Duval County Public Schools: An Analysis of Retention and Performance. Retrieved from https://www.tc.columbia.edu/faculty/ddr2111/facultyprofile/files/FINAL_TFA_DUVAL.pdf

[cl2014001035-bib-00023] Tennessee Higher Education Commission . (2010). Report Card on the Effectiveness of Teacher Training Programs. Nashville, TN: State Board of Education and Tennessee Higher Education Commission.

[cl2014001035-bib-00024] Ware, A., LaTurner, J. R., Parsons, J., Okulicz‐Kozaryn, A., Garland, M., & Klopfenstein, K. (2011). Teacher Preparation Programs and Teach for America Research Study (Rep.). Retrieved from https://www.researchgate.net/publication/236333015_Evaluation_of_Teach_For_America_in_Texas_Schools

[cl2014001035-bib-00025] Xu, Z., Hannaway, J., Taylor, C., & Urban Institute, National Center for Analysis of Longitudinal Data in Education Research . (2009). Making a difference? The effects of Teach for America in high school. Working paper 17. Revised. Washington, DC: National Center for Analysis of Longitudinal Data in Education Research.

[cl2014001035-bib-00026] Xu, Z., Hannaway, J., & Taylor, C. (2011). Making a difference? The effects of Teach for America in high school. Journal of Policy Analysis and Management, 30(3), 447–469.

[cl2014001035-bib-00027] Abbott, S. (Ed.). (2014, December 1). Teacher‐leader. The glossary of education reform. Retrieved from http://edglossary.org/teacher‐leader/

[cl2014001035-bib-00028] Andrabi, T., Das, J., Khwaja, A. I., & Zajonc, T. (2011). Do value‐added estimates add value? Accounting for learning dynamics. American Economic Journal: Applied Economics, 3(3), 29–54. doi:10.1257/app.3.3.29

[cl2014001035-bib-00029] Baker, L. (2016, January 15). Teach For America by the Numbers. Education Week. Retrieved from http://www.edweek.org/ew/section/multimedia/teach‐for‐america‐by‐the‐numbers.html

[cl2014001035-bib-00030] Blazer, C. (2012). What the research says about alternative teacher certification programs. Information Capsule, 1104. Miami, FL: Research Services, Miami‐Dade County Public Schools.

[cl2014001035-bib-00031] Bloom, H. S. (2005). Learning more from social experiments: Evolving analytic approaches. New York, NY: Russell Sage Foundation.

[cl2014001035-bib-00032] Borenstein, M., Hedges, L. V., Higgins, J. P. T., & Rothstein, H. R. (2009). Introduction to meta‐analysis. West Sussex, UK: Wiley & Sons.

[cl2014001035-bib-00033] Boruch, R. F. (1997). Randomized experiments for planning and evaluation: A practical guide. Thousand Oaks, CA: SAGE Publications.

[cl2014001035-bib-00034] Chalmers, I., & Altman, D. G. (Eds.). (1995). Systematic reviews. London, England: BMJ.

[cl2014001035-bib-00035] Clark, M. A., Chiang, H. S., Silva, T., McConnell, S., Sonnenfeld, K., Erbe, A., & Puma, M. (2013). The effectiveness of secondary math teachers from Teach For America and the teaching fellows programs (NCEE 2013‐4016). Jessup, MD: National Center for Education Evaluation and Regional Assistance.

[cl2014001035-bib-00036] Clark, M. A., Isenberg, E., Liu, A. Y., Makowsky, L., & Zukiewicz, M. (2015). Impacts of the Teach For America Investing in Innovation scale‐up (Rep.). Princeton, NJ: Mathematica Policy Research.

[cl2014001035-bib-00037] Clotfelter, C. T., Ladd, H. F., & Vigdor, J. L. (2006). The academic achievement gap in grades 3 to 8 (NBER Working Papers 12207). Cambridge, MA: National Bureau of Economic Research.

[cl2014001035-bib-00038] Cohn, L. D., & Becker, B. J. (2003). How meta‐analysis increases statistical power. Psychological Methods, 8(3), 243–253. Retrieved from 10.1037/1082-989X.8.3.243 14596489

[cl2014001035-bib-00039] Constantine, J., Player, D., Silva, T., Hallgren, K., Grider, M., & Deke, J. (2009). An evaluation of teachers trained through different routes to certification (Final report. NCEE 2009‐4043). Jessup, MD: National Center for Education Evaluation and Regional Assistance.

[cl2014001035-bib-00040] Cooper, H. (2010). Research synthesis and meta‐analysis (4th ed.). Thousand Oaks, CA: SAGE Publications.

[cl2014001035-bib-00041] Darling‐Hammond, L. (1984). Beyond the commission reports. The coming crisis in teaching (RAND No. R3117‐RC). Retrieved from http://www.eric.ed.gov/contentdelivery/servlet/ERICServlet?accno=ED248245

[cl2014001035-bib-00042] Decker, P. T., Mayer, D. P., & Glazerman, S. (2004). The effects of Teach For America on students: Findings from a national evaluation (MPR Reference No.: 8792‐750). Princeton, NJ: Mathematica Policy Research.

[cl2014001035-bib-00043] Fuller, E. J., & Dadey, N. (2013, April 9). Review of evaluation of Teach For America in Texas schools. Retrieved from http://nepc.colorado.edu/thinktank/review‐evaluation‐tfa‐texas

[cl2014001035-bib-00044] Furberg, B., & Furberg, C. (2007). Evaluating clinical research: All that glitters is not gold. New York, NY: Springer Verlag.

[cl2014001035-bib-00045] Glass, G. V. (1976). Primary, Secondary, and Meta‐Analysis of Research. Educational Researcher, 5(10), 3–8. doi:10.3102/0013189x005010003

[cl2014001035-bib-00046] Glazerman, S., Mayer, D., & Decker, P. (2006, Winter). Alternative Routes to Teaching: The Impacts of Teach For America on Student Achievement and Other Outcomes. Journal of Policy Analysis and Management, 25(1), 75–96.

[cl2014001035-bib-00047] Heilig, J. V., & Jez, S. J. (2010). Teach For America: A review of the evidence (Rep.). Retrieved from http://epicpolicy.org/publication/teach‐for‐america

[cl2014001035-bib-00048] Hess, F. M. (2002). Tear down this wall: The case for a radical overhaul of teacher certification. Educational Horizons, 80(4), 169–183.

[cl2014001035-bib-00049] Hunt, M. M. (1997). How science takes stock: The story of meta‐analysis. New York, NY: Russell Sage Foundation.

[cl2014001035-bib-00050] Ingersoll, R. M. (2001). Teacher turnover and teacher shortages: An organizational analysis. American Educational Research Journal, 38(3), 499–534. doi:10.3102/00028312038003499

[cl2014001035-bib-00051] Ingersoll, R. M., & Perda, D. (2010). Is the supply of mathematics and science teachers sufficient? American Educational Research Journal, 47(3), 563–594. doi:10.3102/0002831210370711

[cl2014001035-bib-00052] Kane, T. J., Rockoff, J. E., & Staiger, D. O. (2007). Photo finish: Certification doesn't guarantee a winner. Education Next, 7(1), 60–67.

[cl2014001035-bib-00053] Kizilirmak, P., Ozdemir, O., & Ongen, Z. (2015). The most critical question when reading a meta‐analysis report: Is it comparing apples with apples or apples with oranges? The Anatolian Journal of Cardiology, 15(9), 701–708. doi:10.5152/akd.2014.5665 25334090 10.5152/akd.2014.5665 PMC5368477

[cl2014001035-bib-00054] Klingner, J. K., Boardman, A. G., & McMaster, K. L. (2013). What does it take to scale up and sustain evidence‐based practices? Exceptional Children, 79(2), 195–211.

[cl2014001035-bib-00055] Laczko‐Kerr, I., & Berliner, D. C. (2002). The effectiveness of “Teach For America” and other under‐certified teachers. Education Policy Analysis Archives, 10, 37. doi:10.14507/epaa.v10n37.2002

[cl2014001035-bib-00056] Light, R.J., & Pillemer, D.B., (1984). Summing up: The science of reviewing research. Cambridge, MA: Harvard University Press.

[cl2014001035-bib-00057] Mead, S. (2015). Love ‘em or hate ‘em, here's what you should learn from Teach For America's success. Retrieved from http://www.realcleareducation.com/articles/2015/02/03/teach_for_america_growth_1152.html

[cl2014001035-bib-00058] Monk, D. H. (2007). Recruiting and retaining high‐quality teachers in rural areas. The Future of Children, 17(1), 155–174. doi:10.1353/foc.2007.0009 17407927 10.1353/foc.2007.0009

[cl2014001035-bib-00059] Mosteller, F., & Boruch, R. F. (2002). Evidence matters: Randomized trials in education research. Washington, DC: Brookings Institutions Press.

[cl2014001035-bib-00060] Peske, H. G., & Haycock, K. (2006). Teaching inequality: How poor and minority students are shortchanged on teacher quality: A report and recommendations by the Education Trust. Washington, DC: The Education Trust.

[cl2014001035-bib-00061] Petticrew, M., & Roberts, H. (2006). Systematic reviews in the social sciences: A practical guide. Oxford, UK: Blackwell.

[cl2014001035-bib-00062] Raymond, M., & Fletcher, S. (2002). The Teach For America evaluation. Education Next, 2(1), 62–68.

[cl2014001035-bib-00063] Raymond, M., Fletcher, S. H., & Luque, J. (2001). Teach For America: An evaluation of teacher differences and student outcomes in Houston, Texas. Retrieved from http://credo.stanford.edu/downloads/tfa.pdf

[cl2014001035-bib-00064] Ready, D. D. (2014). Teach For America teachers in Duval County Public Schools: An analysis of retention and performance. Retrieved from https://www.tc.columbia.edu/faculty/ddr2111/faculty‐profile/files/FINAL_TFA_DUVAL.pdf

[cl2014001035-bib-00065] Rothstein, H. R., Sutton, A. J., & Borenstein, M. (Eds.). (2005). Publication Bias in Meta‐Analysis: Prevention, Assessment and Adjustments. Hoboken, NJ: John Wiley & Sons.

[cl2014001035-bib-00066] Seftor, N., & Mayer, D. P. (2003, March 31). The effect of alternative certification on student achievement: A literature review. Unpublished manuscript. Retrieved from http://www.mathematica‐mpr.com/~/media/publications/PDFs/effectalt.pdf

[cl2014001035-bib-00067] Shadish, W. R., Cook, T. D., & Campbell, D. T. (2002). Experimental and quasi‐experimental designs for generalized causal inference. Boston, MA: Houghton Mifflin.

[cl2014001035-bib-00068] Teach For America . (n.d.). What the Research Says. Retrieved from https://www.teachforamerica.org/sites/default/files/what‐the‐research‐says.pdf

[cl2014001035-bib-00069] Teach For America . (2010). Building the movement to eliminate educational inequity Retrieved from http://www.socialimpactexchange.org/files/TFABusinessPlan.pdf

[cl2014001035-bib-00070] Turner, H. M., Goodman, D., Adachi, E., Brite, J., & Decker, L. E. (2012, December). Evaluation of Teach For America in Texas schools. Retrieved from http://edvanceresearch.com/wp‐content/uploads/2015/06/Evaluation‐of‐Teach‐For‐America‐in‐Texas‐Schools.pdf

[cl2014001035-bib-00071] Valentine, J. C., & Cooper, H. (2008). A systematic and transparent approach for assessing the methodological quality of intervention effectiveness research: The Study Design and Implementation Assessment Device (Study DIAD). Psychological Methods, 13(2), 130–149. 18557682 10.1037/1082-989X.13.2.130

[cl2014001035-bib-00072] What Works Clearinghouse . (n.d.). Attrition Standard. WWC Standards Brief. Retrieved from https://ies.ed.gov/ncee/wwc/Docs/referenceresources/wwc_brief_attrition_080715.pdf

[cl2014001035-bib-00073] What Works Clearinghouse . (2014, March). Procedures and Standards Handbook, Version 3.0. Washington, DC: Institute of Education Sciences. Retrieved from https://ies.ed.gov/ncee/wwc/Docs/referenceresources/wwc_procedures_v3_0_standards_handbook.pdf

[cl2014001035-bib-00074] Williams, C. P. (2014, September 24). Stop scapegoating Teach For America. Retrieved from http://www.thedailybeast.com/stop‐scapegoating‐teach‐for‐america

