# Supplementary material for: What are the effects of Teach For America on Math, English Language Arts, and Science outcomes of K–12 students in the USA?
Source: Campbell Syst Rev. 2018 Jun 25;14(1):1–60. doi: 10.4073/csr.2018.7 (PMC8428006; doi:10.4073/csr.2018.7)
Supplement: Supplementary file 1 — Supplementary material [file CL2-14--s002.docx]

**Online supplements**

Appendix A.1: Results from electronic database searches

ERIC (EBSCO)

- *Search 1:* (TI [“Teach for America”] OR AB [“Teach for America” OR “TFA Corps”])
  - Number of Articles: 159
  - Source Type: Academic Journals (64), ERIC Documents (59), Magazines (36), Educational Reports (7), Books (1)
- *Search 2:* (TI [“Teach for America”] OR AB [“Teach for America” OR “TFA Corps”]) **AND (“academic achievement” OR “success*” OR “grade level” OR “grading” OR “academic ability” OR “attainment” OR “failure” OR “educational indicator*”)**
  - Number of Articles: 99
  - Source Type: Academic Journals (41), ERIC Documents (42), Magazines (16), Educational Reports (4)
- *Search 3:* (TI [“Teach for America”] OR AB [“Teach for America” OR “TFA Corps”]) AND (“academic achievement” OR “success*” OR “grade level” OR “grading” OR “academic ability” OR “attainment” OR “failure” OR “educational indicator*”) **AND (“kindergarten” OR “elementary school*” OR “primary school*” OR “high school*” OR “public school”)**
  - Number of Articles: 74
  - Source Type: Academic Journals (36), ERIC Documents (29), Magazines (9), Educational Reports (1)
- *Search 4:* (TI [“Teach for America”] OR AB [“Teach for America” OR “TFA Corps”]) AND (“academic achievement” OR “success*” OR “grade level” OR “grading” OR “academic ability” OR “attainment” OR “failure” OR “educational indicator*”) AND (“kindergarten” OR “elementary school*” OR “primary school*” OR “high school*” OR “public school”) **AND (“random assignment” OR “randomized experiment” OR “experiment*” OR “experimental design” OR “control group” OR “non-experiment” OR “non-experimental” OR “quasi-experiment” OR “quasi-experimental” OR “comparison group” OR “matched comparison group” OR “matched comparison” OR “matched groups” OR “statistical matching” OR “propensity score matching” OR “systematic review” OR “review” OR “meta-analysis” OR “research synthesis” OR “research review”)**
  - Number of Articles: 69
  - Source Type: Academic Journals (32), ERIC Documents (27), Magazines (10), Educational Reports (1)
- *Search 5:* Changing AND (“kindergarten” OR “elementary school*” OR “primary school*” OR “high school*” OR “public school”) to **NOT** changes the count to **67**.

***Seven ERIC references passed initial screening***

1. Clark, M. A., Chiang, H. S., Silva, T., McConnell, S., Sonnenfeld, K., Erbe, A., & Puma, M. (2013). *The effectiveness of secondary math teachers from Teach for America and the teaching fellows programs. NCEE 2013-4015.* Jessup, MD: National Center for Education Evaluation and Regional Assistance.
2. Evans, B. R. (2009). First year middle and high school teachers’ mathematical content proficiency and attitudes: Alternative certification in the Teach for America (TFA) program. *Journal of the National Association for Alternative Certification*, *4*(1), 3–17.
3. Evans, B. R. (2010). Determining quality teachers: Mathematical content knowledge, perceptions of teaching self-efficacy, and attitudes toward mathematics among a Teach for America cohort. *Journal of the National Association for Alternative Certification*, *5*(2), 23–35.
4. Glazerman, S., Mayer, D., & Decker, P. (2006). Alternative routes to teaching: The impacts of Teach for America on student achievement and other outcomes. *Journal of Policy Analysis and Management*, *25*(1), 75–96.
5. Heilig, J. V., & Jez, S. J. (2010). *Teach for America: A review of the evidence.* Boulder and Tempe, AZ: Education and the Public Interest Center & Education Policy Research Unit.
6. Xu, Z., Hannaway, J., & Taylor, C. (2011). Making a difference? The effects of Teach for America in high school. *Journal of Policy Analysis and Management*, *30*(3), 447–469.
7. Xu, Z., Hannaway, J., Taylor, C., & Urban Institute, National Center for Analysis of Longitudinal Data in Education Research. (2009). *Making a difference? The effects of Teach for America in high school. Working paper 17. Revised.* Washington, DC: National Center for Analysis of Longitudinal Data in Education Research.

PsycINFO (ProQuest)

- *Search 1:* (TI [“Teach for America”] OR AB [“Teach for America” OR “TFA Corps”])
  - Number of Articles: 42
  - Source Type: Scholarly Journals (21), Dissertations and Theses (19), Book (2)
- *Search 2:* (TI [“Teach for America”] OR AB [“Teach for America” OR “TFA Corps”]) **AND (“academic achievement” OR “success*” OR “grade level” OR “grading” OR “academic ability” OR “attainment” OR “failure” OR “educational indicator*”)**
  - Number of Articles: 29
  - Source Type: Scholarly Journals (15), Dissertations and Theses (13), Book (1)
- *Search 3:* (TI [“Teach for America”] OR AB [“Teach for America” OR “TFA Corps”]) AND (“academic achievement” OR “success*” OR “grade level” OR “grading” OR “academic ability” OR “attainment” OR “failure” OR “educational indicator*”) **AND (“kindergarten” OR “elementary school*” OR “primary school*” OR “high school*” OR “public school”)**
  - Number of Articles: 25
  - Source Type: Scholarly Journals (14), Dissertations and Theses (10), Book (1)
- *Search 4:* (TI [“Teach for America”] OR AB [“Teach for America” OR “TFA Corps”]) AND (“academic achievement” OR “success*” OR “grade level” OR “grading” OR “academic ability” OR “attainment” OR “failure” OR “educational indicator*”) AND (“kindergarten” OR “elementary school*” OR “primary school*” OR “high school*” OR “public school”) **AND (“random assignment” OR “randomized experiment” OR “experiment*” OR “experimental design” OR “control group” OR “non-experiment” OR “non-experimental” OR “quasi-experiment” OR “quasi-experimental” OR “comparison group” OR “matched comparison group” OR “matched comparison” OR “matched groups” OR “statistical matching” OR “propensity score matching” OR “systematic review” OR “review” OR “meta-analysis” OR “research synthesis” OR “research review”)**
  - Number of Articles: 26
  - Source Type: Scholarly Journals (15), Dissertations and Theses (10), Book (1)
- *Search 5:* Changing AND (“kindergarten” OR “elementary school*” OR “primary school*” OR “high school*” OR “public school”) to **NOT** changes the count to **27**.

***Two PsycINFO references passed initial screening***

1. Glazerman, S., Mayer, D., & Decker, P. (2006). Alternative routes to teaching: The impacts of Teach for America on student achievement and other outcomes.*Journal of Policy Analysis and Management*,*25*(1), 75–96.
2. Xu, Z., Hannaway, J., & Taylor, C. (2011). Making a difference? The effects of Teach for America in high school.*Journal of Policy Analysis and Management*,*30*(3), 44–469.

EconLit (ProQuest)

- *Search 1:* (TI [“Teach for America”] OR AB [“Teach for America” OR “TFA Corps”])
  - Number of Articles: 4
  - Source Type: Scholarly Journals (2), Working Paper (1)
- *Search 2:* (TI [“Teach for America”] OR AB [“Teach for America” OR “TFA Corps”]) **AND (“academic achievement” OR “success*” OR “grade level” OR “grading” OR “academic ability” OR “attainment” OR “failure” OR “educational indicator*”)**
  - Number of Articles: 0
- Search 3: (TI [“Teach for America”] OR AB [“Teach for America” OR “TFA Corps”]) AND (“academic achievement” OR “success*” OR “grade level” OR “grading” OR “academic ability” OR “attainment” OR “failure” OR “educational indicator*”) **AND (“kindergarten” OR “elementary school*” OR “primary school*” OR “high school*” OR “public school”)**
  - Number of Articles: 1
- *Search 4:* (TI [“Teach for America”] OR AB [“Teach for America” OR “TFA Corps”]) AND (“academic achievement” OR “success*” OR “grade level” OR “grading” OR “academic ability” OR “attainment” OR “failure” OR “educational indicator*”) AND (“kindergarten” OR “elementary school*” OR “primary school*” OR “high school*” OR “public school”) **AND (“random assignment” OR “randomized experiment” OR “experiment*” OR “experimental design” OR “control group” OR “non-experiment” OR “non-experimental” OR “quasi-experiment” OR “quasi-experimental” OR “comparison group” OR “matched comparison group” OR “matched comparison” OR “matched groups” OR “statistical matching” OR “propensity score matching” OR “systematic review” OR “review” OR “meta-analysis” OR “research synthesis” OR “research review”)**
  - Number of Articles: 0
- *Search 5:* (TI [“Teach for America”] OR AB [“Teach for America” OR “TFA Corps”]) **AND (“random assignment” OR “randomized experiment” OR “experiment*”** OR “experimental design” OR “control group” OR “non-experiment” OR “non-experimental” OR “quasi-experiment” OR “quasi-experimental” OR “comparison group” OR “matched comparison group” OR “matched comparison” OR “matched groups” OR “statistical matching” OR “propensity score matching” OR “systematic review” OR “review” OR “meta-analysis” OR “research synthesis” OR “research review”)
  - Number of Articles: 5
  - Source Type: Scholarly Journals (4), Working Paper (1)

***Three EconLit references passed initial screening***

1. Carroll, C. A. (2013). *The influence of Teach for America on Algebra I student achievement.* Unpublished doctoral dissertation, University of North Carolina, Charlotte.

2. Glazerman, S., Mayer, D., & Decker, P. (2006). Alternative routes to teaching: The impacts of Teach for America on student achievement and other outcomes.*Journal of Policy Analysis and Management*,*25*(1), 75–96.

1. Xu, Z., Hannaway, J., & Taylor, C. (2011). Making a difference? The effects of Teach for America in high school.*Journal of Policy Analysis and Management*,*30*(3), 447–469.

Sociological Abstracts (ProQuest)

- *Search 1:* (TI [“Teach for America”] OR AB [“Teach for America” OR “TFA Corps”])
  - Number of Articles: 16
  - Source Type: Dissertations and Theses (9), Scholarly Journals (7)
- *Search 2:* (TI [“Teach for America”] OR AB [“Teach for America” OR “TFA Corps”]) **AND (“academic achievement” OR “success*” OR “grade level” OR “grading” OR “academic ability” OR “attainment” OR “failure” OR “educational indicator*”)**
  - Number of Articles: 12
  - Source Type: Dissertations and Theses (8), Scholarly Journals (4)
- *Search 3:* (TI [“Teach for America”] OR AB [“Teach for America” OR “TFA Corps”]) AND (“academic achievement” OR “success*” OR “grade level” OR “grading” OR “academic ability” OR “attainment” OR “failure” OR “educational indicator*”) **AND (“kindergarten” OR “elementary school*” OR “primary school*” OR “high school*” OR “public school”)**
  - Number of Articles: 10
  - Source Type: Dissertations and Theses (6), Scholarly Journals (4)
- *Search 4:* (TI [“Teach for America”] OR AB [“Teach for America” OR “TFA Corps”]) AND (“academic achievement” OR “success*” OR “grade level” OR “grading” OR “academic ability” OR “attainment” OR “failure” OR “educational indicator*”) AND (“kindergarten” OR “elementary school*” OR “primary school*” OR “high school*” OR “public school”) **AND (“random assignment” OR “randomized experiment” OR “experiment*” OR “experimental design” OR “control group” OR “non-experiment” OR “non-experimental” OR “quasi-experiment” OR “quasi-experimental” OR “comparison group” OR “matched comparison group” OR “matched comparison” OR “matched groups” OR “statistical matching” OR “propensity score matching” OR “systematic review” OR “review” OR “meta-analysis” OR “research synthesis” OR “research review”)**
  - Number of Articles: 9
  - Source Type: Dissertations and Theses (5), Scholarly Journals (4)

***Two Sociological Abstracts references passed initial screening***

1. Bastian, K. C. (2014). *Selecting and preparing teachers and school leaders to improve educational outcomes*. Ann Arbor, MI: ProQuest Information & Learning. AAI3562689.
2. Prescott, S. H. (2011). *The effects of affirmative quality feedback on low socio-economic students’ zone of proximal development reading gains (ZPDRL): A causal-comparative study.* Ann Arbor, MI: ProQuest Information & Learning. AAI3447103.

Database: PAIS International (ProQuest)

- *Search 1:* (TI [“Teach for America”] OR AB [“Teach for America” OR “TFA Corps”])
  - Number of Articles: 14
  - Source Type: Scholarly Journals (8), Books (6)
- *Search 2:* (TI [“Teach for America”] OR AB [“Teach for America” OR “TFA Corps”]) **AND (“academic achievement” OR “success*” OR “grade level” OR “grading” OR “academic ability” OR “attainment” OR “failure” OR “educational indicator*”)**
  - Number of Articles: 11
  - Source Type: Scholarly Journals (6), Books (5)
- *Search 3:* (TI [“Teach for America”] OR AB [“Teach for America” OR “TFA Corps”]) AND (“academic achievement” OR “success*” OR “grade level” OR “grading” OR “academic ability” OR “attainment” OR “failure” OR “educational indicator*”) **AND (“kindergarten” OR “elementary school*” OR “primary school*” OR “high school*” OR “public school”)**
  - Number of Articles: 11
  - Source Type: Scholarly Journals (6), Books (5)
- *Search 4:* (TI [“Teach for America”] OR AB [“Teach for America” OR “TFA Corps”]) AND (“academic achievement” OR “success*” OR “grade level” OR “grading” OR “academic ability” OR “attainment” OR “failure” OR “educational indicator*”) AND (“kindergarten” OR “elementary school*” OR “primary school*” OR “high school*” OR “public school”) **AND (“random assignment” OR “randomized experiment” OR “experiment*” OR “experimental design” OR “control group” OR “non-experiment” OR “non-experimental” OR “quasi-experiment” OR “quasi-experimental” OR “comparison group” OR “matched comparison group” OR “matched comparison” OR “matched groups” OR “statistical matching” OR “propensity score matching” OR “systematic review” OR “review” OR “meta-analysis” OR “research synthesis” OR “research review”)**
  - Number of Articles: 11
  - Source Type: Scholarly Journals (6), Books (5)

***Three PAIS International references passed initial screening***

1. Antecol, H., Eren, O., & Ozbeklik, S. (2013). The effect of Teach for America on the distribution of student achievement in primary school: Evidence from a randomized experiment. *Economics of Education Review*, *37*, 113–125.
2. Glazerman, S., Mayer, D., & Decker, P. (2006). Alternative routes to teaching: The impacts of Teach for America on student achievement and other outcomes.*Journal of Policy Analysis and Management*,*25*(1), 75–96.
3. Xu, Z., Hannaway, J., & Taylor, C. (2011). Making a difference? The effects of Teach for America in high school.*Journal of Policy Analysis and Management*,*30*(3), 447–469.

ProQuest Dissertations and Theses, UK and Ireland

- *Search 1:* (TI [“Teach for America”] OR AB [“Teach for America” OR “TFA Corps”])
  - Number of Articles: 1
  - Source Type: Dissertation and Theses
- *Search 2:* (TI [“Teach for America”] OR AB [“Teach for America” OR “TFA Corps”]) **AND (“academic achievement” OR “success*” OR “grade level” OR “grading” OR “academic ability” OR “attainment” OR “failure” OR “educational indicator*”)**
  - Number of Articles: 1
  - Source Type: Dissertation and Theses
- *Search 3:* (TI [“Teach for America”] OR AB [“Teach for America” OR “TFA Corps”]) AND (“academic achievement” OR “success*” OR “grade level” OR “grading” OR “academic ability” OR “attainment” OR “failure” OR “educational indicator*”) **AND (“kindergarten” OR “elementary school*” OR “primary school*” OR “high school*” OR “public school”)**
  - Number of Articles: 1
  - Source Type: Dissertation and Theses
- *Search 4:* (TI [“Teach for America”] OR AB [“Teach for America” OR “TFA Corps”]) AND (“academic achievement” OR “success*” OR “grade level” OR “grading” OR “academic ability” OR “attainment” OR “failure” OR “educational indicator*”) AND (“kindergarten” OR “elementary school*” OR “primary school*” OR “high school*” OR “public school”) **AND (“random assignment” OR “randomized experiment” OR “experiment*” OR “experimental design” OR “control group” OR “non-experiment” OR “non-experimental” OR “quasi-experiment” OR “quasi-experimental” OR “comparison group” OR “matched comparison group” OR “matched comparison” OR “matched groups” OR “statistical matching” OR “propensity score matching” OR “systematic review” OR “review” OR “meta-analysis” OR “research synthesis” OR “research review”)**
  - Number of Articles: 1
  - Source Type: Dissertation and Theses

No references in ProQuest Dissertations and Theses, UK and Ireland, passed initial screening.

ProQuest Dissertations and Theses Global

- *Search 1:* (TI [“Teach for America”] OR AB [“Teach for America” OR “TFA Corps”])
  - Number of Articles: 54; doctoral dissertation only (48)
  - Source Type: Dissertation and Theses
- *Search 2:* (TI [“Teach for America”] OR AB [“Teach for America” OR “TFA Corps”]) **AND (“academic achievement” OR “success*” OR “grade level” OR “grading” OR “academic ability” OR “attainment” OR “failure” OR “educational indicator*”)**
  - Number of Articles: 52; doctoral dissertation only (46)
  - Source Type: Dissertation and Theses
- *Search 3:* (TI [“Teach for America”] OR AB [“Teach for America” OR “TFA Corps”]) AND (“academic achievement” OR “success*” OR “grade level” OR “grading” OR “academic ability” OR “attainment” OR “failure” OR “educational indicator*”) **AND (“kindergarten” OR “elementary school*” OR “primary school*” OR “high school*” OR “public school”)**
  - Number of Articles: 51; doctoral dissertation only (45)
  - Source Type: Dissertation and Theses
- *Search 4:* (TI [“Teach for America”] OR AB [“Teach for America” OR “TFA Corps”]) AND (“academic achievement” OR “success*” OR “grade level” OR “grading” OR “academic ability” OR “attainment” OR “failure” OR “educational indicator*”) AND (“kindergarten” OR “elementary school*” OR “primary school*” OR “high school*” OR “public school”) **AND (“random assignment” OR “randomized experiment” OR “experiment*” OR “experimental design” OR “control group” OR “non-experiment” OR “non-experimental” OR “quasi-experiment” OR “quasi-experimental” OR “comparison group” OR “matched comparison group” OR “matched comparison” OR “matched groups” OR “statistical matching” OR “propensity score matching” OR “systematic review” OR “review” OR “meta-analysis” OR “research synthesis” OR “research review”)**
  - Number of Articles: 51; doctoral dissertation only (45)
  - Source Type: Dissertation and Theses

***Five ProQuest Dissertations and Theses Global References passed initial screening***:

1. Carroll, C. A. (2013). *The influence of Teach for America on Algebra I student achievement.* Unpublished doctoral dissertation, University of North Carolina, Charlotte.
2. Laczko-Kerr, I. I. (2002). *Teacher certification does matter: The effects of certification status on student achievement.* Unpublished doctoral dissertation, Arizona State University, Tempe.
3. Pearson, J. L. (2014). *Effective instructional methods utilized in successful and high performing secondary schools in the Southern Region of Mississippi.* Unpublished doctoral dissertation, The University of Southern Mississippi, Hattiesburg.
4. Penner, E. K. (2014). *Teaching for all? Variation in the effects of Teach for America.* Unpublished doctoral dissertation, University of California, Irvine.
5. Prescott, S. H. (2010). The effects of affirmative quality feedback on low socio-economic students’ zone of proximal development reading gains (ZPDRL): A causal-comparative study. Unpublished doctoral dissertation, University of Mississippi, Oxford.

Database: Worldwide Political Science Abstracts

- *Search 1:* (TI [“Teach for America”] OR AB [“Teach for America” OR “TFA Corps”])
  - Number of Articles: 2
  - Source Type: Scholarly Journal (1) Dissertation and Theses (1)
- *Search 2:* (TI [“Teach for America”] OR AB [“Teach for America” OR “TFA Corps”]) **AND (“academic achievement” OR “success*” OR “grade level” OR “grading” OR “academic ability” OR “attainment” OR “failure” OR “educational indicator*”)**
  - Number of Articles: 1
  - Source Type: Scholarly Journal (1)
- *Search 3:* (TI [“Teach for America”] OR AB [“Teach for America” OR “TFA Corps”]) AND (“academic achievement” OR “success*” OR “grade level” OR “grading” OR “academic ability” OR “attainment” OR “failure” OR “educational indicator*”) **AND (“kindergarten” OR “elementary school*” OR “primary school*” OR “high school*” OR “public school”)**
  - Number of Articles: 1
  - Source Type: Scholarly Journal (1)
- *Search 4:* (TI [“Teach for America”] OR AB [“Teach for America” OR “TFA Corps”]) AND (“academic achievement” OR “success*” OR “grade level” OR “grading” OR “academic ability” OR “attainment” OR “failure” OR “educational indicator*”) AND (“kindergarten” OR “elementary school*” OR “primary school*” OR “high school*” OR “public school”) **AND (“random assignment” OR “randomized experiment” OR “experiment*” OR “experimental design” OR “control group” OR “non-experiment” OR “non-experimental” OR “quasi-experiment” OR “quasi-experimental” OR “comparison group” OR “matched comparison group” OR “matched comparison” OR “matched groups” OR “statistical matching” OR “propensity score matching” OR “systematic review” OR “review” OR “meta-analysis” OR “research synthesis” OR “research review”)**
  - Number of Articles: 1
  - Source Type: Scholarly Journal (1)

No references in Worldwide Political Science Abstracts passed initial screening.

Database: JSTOR

- *Search 1:* (TI [“Teach for America”] OR AB [“Teach for America” OR “TFA Corps”])
  - Number of Articles: 18
  - Source Type: Scholarly Journals
- *Search 2:* (TI [“Teach for America”] OR AB [“Teach for America” OR “TFA Corps”]) **AND (“academic achievement” OR “success*” OR “grade level” OR “grading” OR “academic ability” OR “attainment” OR “failure” OR “educational indicator*”)**
  - Number of Articles: 8
  - Source Type: Scholarly Journals

Search Note: Adding the search terms in bold made the search strategy too long and ineffective. Suggestion: Use **AND (“academic achievement” OR “failure”)**.

- *Search 3:* (TI [“Teach for America”] OR AB [“Teach for America” OR “TFA Corps”]) AND (“academic achievement” OR “success*” OR “grade level” OR “grading” OR “academic ability” OR “attainment” OR “failure” OR “educational indicator*”) **AND (“kindergarten” OR “elementary school*” OR “primary school*” OR “high school*” OR “public school”)**

Search Note: Adding the search terms in bold made the search strategy too long and ineffective. As a result, these terms were not added, and this search strategy was not used with this database.

- *Search 4:* (TI [“Teach for America”] OR AB [“Teach for America” OR “TFA Corps”]) AND (“academic achievement” OR “success*” OR “grade level” OR “grading” OR “academic ability” OR “attainment” OR “failure” OR “educational indicator*”) AND (“kindergarten” OR “elementary school*” OR “primary school*” OR “high school*” OR “public school”) **AND (“random assignment” OR “randomized experiment” OR “experiment*” OR “experimental design” OR “control group” OR “non-experiment” OR “non-experimental” OR “quasi-experiment” OR “quasi-experimental” OR “comparison group” OR “matched comparison group” OR “matched comparison” OR “matched groups” OR “statistical matching” OR “propensity score matching” OR “systematic review” OR “review” OR “meta-analysis” OR “research synthesis” OR “research review”)**
  - Number of Articles: 4
  - Source Type: Scholarly Journals

Search Note: Adding the search terms in bold made the search strategy too long and ineffective. Therefore, we only used **“AND** **(experiment*)”**.

***One reference in JSTOR passed initial screening:***

1. Glazerman, S., Mayer, D., & Decker, P. (2006). Alternative routes to teaching: The impacts of Teach for America on student achievement and other outcomes.*Journal of Policy Analysis and Management*,*25*(1), 75–96.

Database: Academic Search Premier

- *Search 1:* (TI [“Teach for America”] OR AB [“Teach for America” OR “TFA Corps”])
  - Number of Articles: 432
  - Source Type: Magazines (283), Academic Journals (42), Newspapers (86), Reviews (17), Trade Publications (3)
- *Search 2:* (TI [“Teach for America”] OR AB [“Teach for America” OR “TFA Corps”]) **AND (“academic achievement” OR “success*” OR “grade level” OR “grading” OR “academic ability” OR “attainment” OR “failure” OR “educational indicator*”)**
  - Number of Articles: 138
  - Source Type: Magazines (85), Academic Journals (25), Newspapers (19), Reviews (9)
- *Search 3:* (TI [“Teach for America”] OR AB [“Teach for America” OR “TFA Corps”]) AND (“academic achievement” OR “success*” OR “grade level” OR “grading” OR “academic ability” OR “attainment” OR “failure” OR “educational indicator*”) **AND (“kindergarten” OR “elementary school*” OR “primary school*” OR “high school*” OR “public school”)**
  - Number of Articles: 98
  - Source Type: Magazines (55), Academic Journals (20), Newspapers (14), Reviews (9)
- *Search 4:* (TI [“Teach for America”] OR AB [“Teach for America” OR “TFA Corps”]) AND (“academic achievement” OR “success*” OR “grade level” OR “grading” OR “academic ability” OR “attainment” OR “failure” OR “educational indicator*”) AND (“kindergarten” OR “elementary school*” OR “primary school*” OR “high school*” OR “public school”) **AND (“random assignment” OR “randomized experiment” OR “experiment*” OR “experimental design” OR “control group” OR “non-experiment” OR “non-experimental” OR “quasi-experiment” OR “quasi-experimental” OR “comparison group” OR “matched comparison group” OR “matched comparison” OR “matched groups” OR “statistical matching” OR “propensity score matching” OR “systematic review” OR “review” OR “meta-analysis” OR “research synthesis” OR “research review”)**
  - Number of Articles: 92
  - Source Type: Magazines (52), Academic Journals (19), Newspapers (13), Reviews (8)

***One reference in Academic Search Premier passed initial screening***

1. Antecol, H., Eren, O., & Ozbeklik, S. (2013). The effect of Teach for America on the distribution of student achievement in primary school: Evidence from a randomized experiment. *Economics of Education Review*, *37*, 113–125.

Database: Education Full Text

- *Search 1:* (TI [“Teach for America”] OR AB [“Teach for America” OR “TFA Corps”])
  - Number of Articles: 408
  - Source Type: Magazines (253), Academic Journals (105), Book Reviews (23), Books (15), Newspapers (4), Conference Papers (4), Biographies (1)
- *Search 2:* (TI [“Teach for America”] OR AB [“Teach for America” OR “TFA Corps”]) **AND (“academic achievement” OR “success*” OR “grade level” OR “grading” OR “academic ability” OR “attainment” OR “failure” OR “educational indicator*”)**
  - Number of Articles: 172
  - Source Type: Magazines (89), Academic Journals (65), Book Reviews (12), Conference Papers (3), Newspapers (2), Books (2)
- *Search 3:* (TI [“Teach for America”] OR AB [“Teach for America” OR “TFA Corps”]) AND (“academic achievement” OR “success*” OR “grade level” OR “grading” OR “academic ability” OR “attainment” OR “failure” OR “educational indicator*”) **AND (“kindergarten” OR “elementary school*” OR “primary school*” OR “high school*” OR “public school”)**
  - Number of Articles: 139
  - Source Type: Magazines (70), Academic Journals (50), Book Reviews (12), Conference Papers (2), Newspapers (1), Books (1)
- *Search 4:* (TI [“Teach for America”] OR AB [“Teach for America” OR “TFA Corps”]) AND (“academic achievement” OR “success*” OR “grade level” OR “grading” OR “academic ability” OR “attainment” OR “failure” OR “educational indicator*”) AND (“kindergarten” OR “elementary school*” OR “primary school*” OR “high school*” OR “public school”) **AND (“random assignment” OR “randomized experiment” OR “experiment*” OR “experimental design” OR “control group” OR “non-experiment” OR “non-experimental” OR “quasi-experiment” OR “quasi-experimental” OR “comparison group” OR “matched comparison group” OR “matched comparison” OR “matched groups” OR “statistical matching” OR “propensity score matching” OR “systematic review” OR “review” OR “meta-analysis” OR “research synthesis” OR “research review”)**
  - Number of Articles: 128
  - Source Type: Magazines (63), Academic Journals (50), Book Reviews (11), Conference Papers (2), Newspapers (1), Books (1)

***Four references in Education Full Text passed the initial screening***

1. Antecol, H., Eren, O., & Ozbeklik, S. (2013). The effect of Teach for America on the distribution of student achievement in primary school: Evidence from a randomized experiment. *Economics of Education Review*, *37*, 113–125.

1. Darling-Hammond, L., Holtzman, D. J., & Gatlin, S. J. (2005). Does teacher preparation matter? Evidence about teacher certification, Teach for America, and teacher effectiveness. *Education Policy Analysis Archives*,*13*(42), 1–47.
2. Glazerman, S., Mayer, D., & Decker, P. (2006). Alternative routes to teaching: The impacts of Teach for America on student achievement and other outcomes.*Journal of Policy Analysis and Management*,*25*(1), 75–96.
3. Xu, Z., Hannaway, J., & Taylor, C. (2011). Making a difference? The effects of Teach for America in high school.*Journal of Policy Analysis and Management*,*30*(3), 447–469.

Appendix A.2: Results from grey literature search

Table A.2.1. *Results from Searches of General and Targeted Websites*

|  | **Searching** | | | | | | |  | **Screening** | | | | | | |
| --- | --- | --- | --- | --- | --- | --- | --- | --- | --- | --- | --- | --- | --- | --- | --- |
|  | Website | | Term(s) | | Citations | | |  | Eligible | | Duplicate | | | Result | |
| 1 | Abt Associates | | Teach for America, TFA, or Corps | | 0 | | |  | 0 | | 0 | | | Upcoming project: “Study on Promising Teacher Prep Programs” | |
| 2 | Alliance for Excellent Education | | TFA | | 3 | | |  | 0 | | 0 | | | All reports reference TFA as a source but not as a study | |
| AERA Online Journals: | |  | |  | |  |  | | |  | | |  | |  |
| 3 | AERA Open | | TFA | | 1 | | |  | 0 | | 0 | | | TFA referenced as an example, not a study | |
| 4 | AERJ | | TFA | | 4 | | |  | 0 | | 0 | | | No studies used a comparison group | |
| 5 | EEPA | | TFA | | 6 | | |  | 0 | | 0 | | | TFA referenced as a source but not a study | |
| 6 | ER | | TFA | | 0 | | |  | 0 | | 0 | | | – | |
| 7 | JEBS | | TFA | | 3 | | |  | 0 | | 0 | | | Reviews of TFA; potential source for other studies | |
| 8 | RER | | TFA | | 1 | | |  | 0 | | 0 | | | Reviews of TFA; potential source for other studies | |
| 9 | RRE | | TFA | | 1 | | |  | 0 | | 0 | | | Reviews of TFA; potential source for other studies | |
| 10 | American Enterprise Institute | | TFA | | 27 | | |  | 0 | | 0 | | | Opinion articles about TFA | |
| 11 | American Institutes for Research | | TFA | | 5 | | |  | 0 | | 0 | | | Mostly news reports | |
| 12 | Best Evidence Encyclopedia | | TFA | | 0 | | |  | 0 | | 0 | | | – | |
| 13 | Brookings Institute | | TFA | | 10 | | |  | 0 | | 0 | | | Blogs, memos, etc., but no studies | |
| 14 | Carnegie Corporation of New York | | Teach for America | | 4 | | |  | 0 | | 0 | | | Reports reference TFA but were not studies | |
| 15 | Center for Research and Reform in Education | | – | | 0 | | |  | 0 | | | 0 | | Website platform not conducive to a systematic search | |
| 16 | Congressional Research Service | | – | | 0 | | |  | 0 | | | 0 | | Website platform not conducive to a systematic search | |

Table A.2.1 (continued)

|  | **Searching** | | |  | **Screening** | | | |
| --- | --- | --- | --- | --- | --- | --- | --- | --- |
|  | Website | Term(s) | Citations |  | Eligible | Duplicate | Result | |
| 17 | Government Accountability Office | – | 0 |  | 0 | 0 | Website platform not conducive to a systematic search | |
| 18 | Grants and contracts awarded by IES | TFA | 4 |  | 0 | 0 | None were publications | |
| 19 | Heritage Foundation | – | 0 |  | 0 | 0 | Website platform not conducive to a systematic search | |
| 20 | Hoover Institute | TFA | 7 |  | 0 | 0 | Mostly news reports and articles describing TFA research | |
| 21 | Mathematica Policy Research | TFA | 101 |  | 4 | 3 | Identified a literature review | |
| 22 | MDRC | TFA | 1 |  | 0 | 0 | Conference report | |
| 23 | National Association of State Boards of Education | TFA | 5 |  | 0 | 0 | Narrative reviews and report but no studies | |
| 24 | National Governors’ Association | TFA | 1 |  | 0 | 0 | News reports | |
| 25 | Policy Archive | – | 0 |  | 0 | 0 | Website platform not conducive to a systematic search | |
| 26 | Policy Study Associates | TFA | 10 |  | 0 | 0 | Studies were not impact evaluations with comparison group | |
| 27 | RAND | TFA | 4 |  | 0 | 0 | Studies did not use a comparison group | |
| 28 | SRI | TFA | 4 |  | 0 | 0 | Reports did not use a comparison group |  |
| 29 | Thomas B. Fordham Institute | – | 0 |  | 0 | 0 | Website platform not conducive to a systematic search |  |
| 30 | Urban Institute | TFA | 7 |  | 0 | 0 | Studies did not use a comparison group |  |
| 31 | After-School Alliance | TFA | 15 |  | 0 | 0 | News reports, not studies |  |
| 32 | The Campbell Collaboration | TFA | 3 |  | 0 | 0 | No new research beyond the current systematic review |  |

Table A.2.1 (continued)

|  | **Searching** | | |  | **Screening** | | |
| --- | --- | --- | --- | --- | --- | --- | --- |
|  | Website | Term(s) | Citations |  | Eligible | Duplicate | Result |
| 33 | Carnegie Corporation for the Advancement of Teaching | ­­– | 0 |  | 0 | 0 | Website platform not conducive to a systematic search |
| 34 | Center for Social Organization of Schools—BERC | TFA | 0 |  | 0 | 0 | – |
| 35 | Chapin Hall Center for Children | TFA | 0 |  | 0 | 0 | – |
| 36 | CINAHL | TFA | 2 |  | 0 | 0 | Reports do not use a comparison group |
| 37 | Cochrane Central Register of Controlled Trial | TFA | 0 |  | 0 | 0 | – |
|  |  | Teach for America | 0 |  | 0 | 0 | – |
| 38 | Cochrane Database of Systematic Reviews | TFA | 0 |  | 0 | 0 | – |
|  |  | Teach for America | 0 |  | 0 | 0 | – |
| 39 | Database of Abstracts of Reviews of Effects | TFA | 0 |  | 0 | 0 | – |
| 40 | Florida Center for Reading Research | – | 0 |  | 0 | 0 | Website platform not conducive to a systematic search |
|  |  | Teach for America | 0 |  | 0 | 0 | – |
| 41 | Harvard Family Research Project | TFA | 0 |  | 0 | 0 | Opinion articles |
| 42 | Institute for Higher Education Policy | TFA | 0 |  | 0 | 0 | – |
| 43 | Institute for Public Policy and Social Research | TFA | 0 |  | 0 | 0 | – |
| 44 | Natl. Association of State Directors of Career Tech. Ed. | – | 0 |  | 0 | 0 | Website platform not conducive to a systematic search |
| 44 | NBER Working Papers | TFA | 6 |  | 0 | 0 | Valued-added or RDD studies |
|  | **Total** |  | **235** |  | **4** | **3** |  |

**One website reference passed initial screening**

1. Clark, M. A., Isenberg, E., Liu, A. Y., Makowsky, L., & Zukiewicz, M. (2016, March 4). *Impacts of the Teach for America Investing in Innovation scale-up.* Retrieved from <http://www.mathematica-mpr.com/our-publications-and-findings/publications/impacts-of-the-teach-for-america-investing-in-innovation-scaleup>

Table A.2.2. *Results from Searches of Grey Literature Databases (Including Conference Proceedings and Google)*

|  | **Searching** | | |  |  | | **Screening** | |  | |
| --- | --- | --- | --- | --- | --- | --- | --- | --- | --- | --- |
|  | Website | Term(s) | Citations |  | Relevant | Duplicate | | Result | |  |
| 1 | PolicyFile | Teach for America | 12 |  | 4 | 4 | | Four studies retrieved from previous database searches | | |
|  |  | TFA | 0 |  | 0 | 0 | |  | | |
| 2 | PsycEXTRA | – | – |  | – | – | | Website not conducive to a systematic search | | |
| 3 | OpenGrey.eu | TFA | 1 |  | 0 | 0 | |  | | |
| 4 | EditLib | Teach for America | 14 |  | 0 | 0 | |  | | |
|  |  | TFA | 10 |  | 0 | 0 | |  | | |
| 5 | Index of Conference Proceedings | – | – |  | – | – | | Website not conducive to a systematic search | | |
| 6 | Google | (“Teach for America” or “TFA”) AND (“Math” or “Science” or “Language Arts”) AND (“impact” or “effect”) | 359 |  | 23 | 14 | | Identified six studies that were not identified in previous searches. | | |
|  | **Total** |  | **396** |  | **27** | **18** | |  | | |

***Eight references from grey literature database searches passed the screening and were not duplicates from previous database searches***

1. Boyd, D., Grossman, P., Lankford, H., Loeb, S., & Wyckoff, J. (2006). *How changes in entry requirements alter the teacher workforce and affect student achievement.* Columbia, MO: American Education Finance Association. Retrieved from [https://cepa.stanford.edu/sites/default/files/Reducing Entry Requirements EPF 2006.pdf](https://cepa.stanford.edu/sites/default/files/Reducing%20Entry%20Requirements%20EPF%202006.pdf)

1. Noell, G. H., & Gansle, K. A. (2009). *Teach For America teachers’ contribution to student achievement in Louisiana in grades 4–9: 2004–2005 to 2006–2007.* Baton Rouge, LA: Louisiana Board of Regents.
2. Henry, G. T., Thompson, C. L., Bastian, K. C., Fortner, K. C., Kershaw, D. C., Purrell, K. M., & Zulli, R. A. (2010, June). *Portal report: Teacher preparation and student test scores in North Carolina.* Retrieved from <http://www.worldcat.org/title/portal-report-teacher-preparation-and-student-test-scores-in-north-carolina/oclc/789248583>
3. Ware, A., LaTurner, J. R., Parsons, J., Okulicz-Kozaryn, A., Garland, M., & Klopfenstein, K. (2011). *Teacher preparation programs and Teach for America research study* (Rep.). Retrieved from <https://www.researchgate.net/publication/236333015_Evaluation_of_Teach_For_America_in_Texas_Schools>
4. Turner, H. M., Goodman, D., Adachi, E., Brite, J., & Decker, L. E. (2012, December). *Evaluation of Teach for America in Texas schools.* Retrieved from <http://http://edvanceresearch.com/wp-content/uploads/2015/06/Evaluation-of-Teach-For-America-in-Texas-Schools.pdf>
5. Ready, D. D. (2014). *Teach for America teachers in Duval County public schools: An analysis of retention and performance.* Retrieved from <https://www.tc.columbia.edu/faculty/ddr2111/faculty-profile/files/FINAL_TFA_DUVAL.pdf>
6. Boyd, D., Grossman, P., Hammerness, K., Lankford, H., Loed, S., Ronfeldt, M., & Wyckoff, J. (2012). Recruiting effective math teachers: Evidence from New York City. *American Educational Research Journal*, *49*(6), 1008–1047.
7. Clark, M. A., Isenberg, E., Liu, A. Y., Makowsky, L., & Zukiewicz, M. (2016, March 4). *Impacts of the Teach for America Investing in Innovation scale-up.* Retrieved from <http://www.mathematica-mpr.com/our-publications-and-findings/publications/impacts-of-the-teach-for-america-investing-in-innovation-scaleup>

Appendix A.3: Study referrals

Table A.3.1. *Study Referrals from a Random Sample of Researchers*

|  | **Researcher** | **Referral** | **Duplicate**  **Study** |
| --- | --- | --- | --- |
| 1 | Dan Goldhaber | None. | – |
| 2 | Gene Glass | Laczko-Kerr, I., & Berliner, D. C. (2002, September 6). The effectiveness of “Teach for America” and other under-certified teachers on student academic achievement: A case of harmful public policy. *Education Policy Analysis Archives*, *10*(37). Retrieved from <http://epaa.asu.edu/epaa/v10n37> | Yes |
| 3 | Lawrence Baines | Anecdote on TFA corps members. |  |
| 4 | Linda Darling-Hammond | Assistant responded that they did not have time to provide data. |  |
| 5 | Melissa Clark | Clark, M. A., Chiang, H. S., Silva, T., McConnell, S., Sonnenfeld, K., Erbe, A., & Puma, M. (2013). *The effectiveness of secondary math teachers from Teach for America and the teaching fellows programs* (NCEE 2013-4015). Washington, DC: National Center for Education Evaluation and Regional Assistance, Institute of Education Sciences, U.S. Department of Education. | Yes |
|  |  | Decker, P., Mayer, D., & Glazerman, S. (2004). *The effects of Teach for America on students: Findings from a national evaluation*. Princeton, NJ: Mathematica Policy Research, Inc. | Yes |
|  |  | Clark, M. A., Isenberg, E., Liu, A. Y., Makowsky, L., & Zukiewicz, M. (2016, March 4). *Impacts of the Teach for America Investing in Innovation scale-up.* Retrieved from <http://www.mathematica-mpr.com/our-publications-and-findings/publications/impacts-of-the-teach-for-america-investing-in-innovation-scaleup> | Yes |

Note: These 5 researchers were randomly selected from a list of 25 researchers identified via the reference list from cursory searches used during protocol development.

Table A.3.2. *Random Sampling of Researchers*

| **Author Sampling Frame** | |  | **Author Sample** | | |
| --- | --- | --- | --- | --- | --- |
| **Author**  **Last Name** | **Random Number** |  | **Author**  **Last Name** | **Random Number (Sorted)** | **Selected** |
| Xu | 0.435 |  | Clark | 0.056 | X |
| Fetler | 0.284 |  | Goldhaber | 0.112 | X |
| Clofelter | 0.428 |  | Darling-Hammond | 0.180 | X |
| Constantine | 0.618 |  | Baines | 0.240 | X |
| Noell | 0.776 |  | Glass | 0.251 | X |
| Hess | 0.696 |  | Decker | 0.276 |  |
| Goldhaber | 0.112 |  | Fetler | 0.284 |  |
| Veltri | 0.388 |  | Laczko-Kerr | 0.290 |  |
| Helig | 0.464 |  | Wilson | 0.346 |  |
| Jones | 0.899 |  | Kane | 0.387 |  |
| Darling-Hammond | 0.180 |  | Veltri | 0.388 |  |
| Boyd | 0.513 |  | Baines | 0.393 |  |
| Ware | 0.595 |  | Clofelter | 0.428 |  |
| Parsons | 0.833 |  | Xu | 0.435 |  |
| Decker | 0.276 |  | Helig | 0.464 |  |
| Antecol | 0.951 |  | Boyd | 0.513 |  |
| Wilson | 0.346 |  | Ware | 0.595 |  |
| Baines | 0.393 |  | Constantine | 0.618 |  |
| Clark | 0.056 |  | Hess | 0.696 |  |
| Glass | 0.251 |  | Raymond | 0.697 |  |
| Laczko-Kerr | 0.290 |  | Noell | 0.776 |  |
| Kane | 0.387 |  | Parsons | 0.833 |  |
| Raymond | 0.697 |  | Goe | 0.890 |  |
| Baines | 0.240 |  | Jones | 0.899 |  |
| Goe | 0.890 |  | Antecol | 0.951 |  |

Note: The author sample consisted of the five authors with the lowest random numbers.

| **From:** Michèle Muñoz-Miller [<mailto:michele@analytica-inc.com>]  **Sent:** Tuesday, May 12, 2015 9:34 PM  **To:** Melissa Clark <[MClark@mathematica-mpr.com](mailto:MClark@mathematica-mpr.com)>  **Subject:** Request for TFA citations    Hello Dr. Clark,    A research team at ANALYTICA is conducting a Campbell Collaboration systematic review on the impact of Teach For America on student academic achievement.    We are reaching out to researchers for references to randomized controlled trials or quasi-experiments with a comparison group that may be relevant to the review.    We would appreciate referrals to relevant citations from you.    Thanks in advance for your help.    Michèle    ANALYTICA, Inc.  35 Goldfinch Circle \| Phoenixville \| PA \| 19460  T: 215.808.8880 \| F: 610.933.1005 \| |
| --- |

Appendix A.4: Results from hand searches of journals

Table A.4. *Results of Hand Searches of Selected Journals for 2014–2015*

| **Searching** | | | **Screening** | | | | |
| --- | --- | --- | --- | --- | --- | --- | --- |
| No. | Journal | Terms | Articles | Relevant | Duplicate | | Volume and Issues Reviewed |
| 1 | *American Educational Research* | Teach for America or TFA | 5 | 0 | 0 | | Reviewed 2014 Volume 51 (6 issues)  Reviewed 2015 Volume 52 (6 issues) |
| 2 | *American Economic Association* | – | 0 | 0 | 0 | | Website platform not conducive to a systematic hand search of journals |
| 3 | *Journal of Policy Analysis and Management* | Teach for America or TFA | 1 | 1 | 0 | | Reviewed 2014 Volume 33 (4 issues)  Reviewed 2015 Volume 34 (4 issues)  Reviewed 2016 Volume 35 (1 issue) |
| 4 | *Economics of Education Review* | Teach for America or TFA | 0 | 0 | 0 | | Reviewed 2014-2016 Volumes 41–50 |
| 5 | *Education Policy Analysis Archives* | Teach for America or TFA | 12 | 0 | 0 | | Website platform not conducive to a systematic hand search of journals |
| 6 | *Journal of Human Resources* | Teach for America or TFA | 0 | 0 | 0 | | Reviewed 2014 Volume 49 (4 journals)  Reviewed 2015 Volume 50 (4 journals)  Reviewed 2016 Volume 51 (1 journal) |
|  | **Total** |  | **17** |  | **1** | **0** |  |

***One reference from hand searches passed initial screening***

1. Dee, T. S., & Wyckoff, J. (2015). Incentives, selection, and teacher performance: Evidence from IMPACT. *Journal of Policy Analysis and Management*, *34*(2), 267–297.

Appendix B: Results of author queries

Table B.1. *Status of Author Queries for Studies Eligible for Inclusion in the Systematic Review*

| **Study Reference** | **Primary Contacts** | **Status** | **Explanation** |
| --- | --- | --- | --- |
| Antecol, H., Eren, O., & Ozbeklik, S. (2013). The effect of Teach for America on the distribution of student achievement in primary school: Evidence from a random experiment. *Economics of Education Review*, *37*, 113–125. | Heather Antecol | **No response** | Sent author query twice. |
| Boyd, D., Grossman, P., Lankford, H., Loeb, S., & Wyckoff, J. (2006). *How changes in entry requirements alter the teacher workforce and affect student achievement.* Columbia, MO: American Education Finance Association. Retrieved from [https://cepa.stanford.edu/sites/default/files/Reducing Entry Requirements EPF 2006.pdf](https://cepa.stanford.edu/sites/default/files/Reducing%20Entry%20Requirements%20EPF%202006.pdf) | Don Boyd | **Responded** | Boyd said data are not available due to confidentiality agreements with Texas Education Agency (TEA). Data have been discarded. |
| Boyd, D., Grossman, P., Hammerness, K., Lankford, H., Loed, S., Ronfeldt, M., & Wyckoff, J. (2012). Recruiting effective math teachers: Evidence from New York City. *American Educational Research Journal*, *49*(6), 1008–1047. | Don Boyd | **Responded** | Boyd said data are not available due to confidentiality agreements with TEA. Data have been discarded. |
| Carroll, C. A. (2013). *The influence of Teach for America on Algebra I student achievement.* Unpublished doctoral dissertation, University of North Carolina, Charlotte. | Curtis Carroll | **No response** | Sent author query twice. |
| Clark, M., Chiang, H., Silva, T., McConnell, S., Sonnenfeld, K., & Erbe, A. (2013, September). *The effectiveness of secondary math teachers from Teach for America and the teaching fellow programs.* NCEE 2013-4016. Washington, DC: U.S. Department of Education. | [Melissa](mailto:mclark@mathematica-mpr.com) Clark | **Responded** | Clark sent data. |
| Clark, M. A., Isenberg, E., Liu, A. Y., Makowsky, L., & Zukiewicz, M. (2016, March 4). *Impacts of the Teach for America Investing in Innovation scale-up.* Retrieved from <http://www.mathematica-mpr.com/our-publications-and-findings/publications/impacts-of-the-teach-for-america-investing-in-innovation-scaleup> | Melissa Clark | **Responded** | Clark sent data. |

Table B.1 (continued)

| **Study Reference** | **Primary  Contacts** | **Status** | **Explanation** |
| --- | --- | --- | --- |
| Darling-Hammond, L., Holtzman, D. J., Gatlin, S. J., & Heilig, J. V. (2005). Does teacher preparation matter? Evidence about teacher certification, Teach for America, and teacher effectiveness. *Education Policy Analysis Archives*, *13*(42). Retrieved from <http://epaa.asu.edu/epaa/v13n42/> | Linda Darling-Hammond | **Responded** | Student of Dr. Darling-Hammond said data are not available. |
| Decker, P., Mayer, D., & Glazerman, S. (2004). *The effects of Teach for America on students: Findings from a national evaluation*. Princeton, NJ: Mathematica Policy Research, Inc. | [Steve](mailto:sglazerman@mathematica-mpr.com) Glazerman | **Responded** | Glazerman sent public use file and responded to questions. |
| Noell, G. H., & Gansle, K. A. (2009). *Teach For America teachers’ contribution to student achievement in Louisiana in grades 4–9: 2004–2005 to 2006–2007.* Baton Rouge, LA: Louisiana Board of Regents. | George Noell | **Responded** | Sent data as an Excel spreadsheet |
| Ware, A., LaTurner, R., Parsons, J., Okulicz-Kozaryn, A., Garland, M., & Klopfenstein, K. (2011). *Teacher preparation programs and Teach for America research Study*. Dallas, TX: The University of Texas at Dallas Education Research Center. | Anne Ware | **Responded** | Ware requested a copy of study. A copy was sent to her, and she reviewed it, but she did not send any data in response. |

Figure B.1. *Example of author query email.*

| Dear [INSERT AUTHOR NAME],    I hope all is well with you.    ANALYTICA was awarded a Campbell Collaboration grant to conduct the first-ever high-quality systematic review on the impact of Teach for America on student academic outcomes. Your study, cited below, has been selected for inclusion in this review:  [Insert Study Citation]    We plan to present the information from your study as accurately as possible, especially since this review will likely garner high visibility both domestically and internationally.    The attached document requests data needed to accurately evaluate the quality of the evidence presented in your study. **We would like to receive this information by [INSERT DATE].**    However, we understand that we are in the holiday season and you may need more time. If so, please contact me about a feasible date. **If I do not hear from you by [INSERT DATE], we will proceed based on the information published in the study.**    Thanks,    Herb    Herbert M. Turner, III \| President & Principal Scientist \| ANALYTICA, Inc.  Adjunct Associate Professor \| PENN GSE  35 Goldfinch Circle \| Phoenixville \| PA \| 19460  T: 215.808.8880 \| F: 610.933.1005 \| |
| --- |

Appendix C: Coding results for review-eligible studies (*n* = 24)

Table C.1. *Coding Results for Studies that Were Eligible for Review and Coding*

| **No.** | **Studies Eligible for Coding** | **Coding Result** | | | | |
| --- | --- | --- | --- | --- | --- | --- |
|  |  | Design | Passed  Stage1 | Passed  Stage 2 | Author Query | In Meta- Analysis |
| 1 | Antecol, H., Eren, O., & Ozbeklik, S. (2013) | RCT | Yes | No | Yes | No |
| 2 | Bastian, K. C. (2014) | TVA | Yes | No | Yes | No |
| 3 | Boyd, D., Grossman, P., Lankford, H., Loeb, S., & Wyckoff, J. (2006) | TVA | Yes | No | Yes | No |
| 4 | Boyd, D., Grossman, P., Hammerness, K., Lankford, H., Loed, S., Ronfeldt, M., & Wyckoff, J. (2012) | TVA | Yes | No | Yes | No |
| 5 | Carroll, C. A. (2013) | QED | Yes | No | Yes | No |
| 6 | Clark, M. A., Chiang, H., Silva, T., McConnell, S., Sonnenfeld, K., & Erbe, A. (2013) | RCT | Yes | Yes | Yes | Yes |
| 7 | Clark, M. A., Isenberg, E., Liu, A. Y., Makowsky, L., & Zukiewicz, M. (2016) | RCT | Yes | Yes | Yes | Yes |
| 8 | Darling-Hammond, L., Holtzman, D. J., & Gatlin, S. J. (2005) | QED | Yes | No | Yes | No |
| 9 | Dee, T. S., & Wyckoff, J. (2015) | RDD | No | - | - | No |
| 10 | Evans, B. R. (2009) | COR | N0 | - | - | No |
| 11 | Evans, B. R. (2010) | COR | No | - | - | No |
| 12 | Decker, P., Mayer, D., & Glazerman, S. (2004); Glazerman, S., Mayer, D., & Decker, P. (2006) | RCT | Yes | Yes | Yes | Yes |
| 13 | Henry, G. T., Thompson, C. L., Bastian, K. C., Fortner, K. C., Kershaw, D. C., Purrell, K. M., & Zulli, R. A. (2010) | TVA | Yes | No | Yes | No |
| 14 | Laczko-Kerr, I. I. (2002) | QED | Yes | No | Yes | No |
| 15 | Noell, G., & Gansle, K. A. (2009) | TVA | Yes | No | Yes | No |
| 16 | Pearson, J. L. (2014) | TVA | Yes | No | Yes | No |
| 17 | Penner, E. K. (2014) | QED | Yes | No | No | No |
| 18 | Prescott, S. H. (2010) | QED | No | - | - | No |
| 19 | Raymond, M., Fletcher, S., & Luque, J. (2001); Raymond, M., & Fletcher, S. (2002) | QED | Yes | No | No | No |
| 20 | Ready (2014) | QED | Yes | No | No | No |
| 21 | Tennessee State Board and Higher Education Commission (2010) | QED | Yes | No | No | No |
| 22 | Turner, H. M., Goodman, D., Adachi, E., Brite, J., & Decker, L. E. (2012) | QED | Yes | Yes | No | Yes |
| 23 | Ware, A., LaTurner, R., Parsons, J., Okulicz-Kozaryn, A., Garland, M., & Klopfenstein, K. (2011) | QED | Yes | Yes | Yes | No |
| 24 | Xu, Z., Hannaway, J., & Taylor, C. (2009); Xu, Z., Hannaway, J., & Taylor, C. (2011) | QED | Yes | No | Yes | No |
| **Total** | | **24** | **21** | **4** | **15** | **4** |

Note: TVA = “Teacher Value Added,” RDD = “Regression Discontinuity Design,” and LR = “Literature Review.” Study 1 used the same data as study 10, for different reasons.

Table C.2. *Request for Study Information from Authors of TFA Studies that Passed Stage 1 or Stage 2 Screening*

| **No.** | **Study Reference** | **Primary Contact** | **Response** | **Explanation** |
| --- | --- | --- | --- | --- |
| 1 | Antecol, H., Eren, O. & Ozbeklik, S. (2013). The effect of Teach for America on the distribution of student achievement in primary school: Evidence from a random experiment. *Economics of Education Review*, *37*, 113–125. | Heather Antecol | **No** | Sent author query twice. |
| 3 | Boyd, D., Grossman, P., Lankford, H., Loeb, S., & Wyckoff, J. (2006). *How changes in entry requirements alter the teacher workforce and affect student achievement. Columbia, MO: American Education Finance Association*. Retrieved from https://cepa.stanford.edu/sites/default/files/Reducing Entry Requirements EPF 2006.pdf | Dan Boyd | **Yes** | Data were destroyed. |
| 4 | Boyd, D., Grossman, P., Hammerness, K., Lankford, H., Loed, S., Ronfeldt, M., & Wyckoff, J. (2012). Recruiting effective math teachers: Evidence from New York City. American Educational Research Journal, 49(6), 1008–1047. | Dan Boyd | **Yes** | Data were destroyed. |
| 5 | Carroll, C. A. (2013). *The influence of Teach for America on Algebra I student achievement.* Unpublished doctoral dissertation, University of North Carolina, Charlotte. | Curtis Carroll | **No** | Sent author query twice. |
| 6 | Clark, M., Chiang, H., Silva, T., McConnell, S., Sonnenfeld, K., & Erbe, A. (2013, September). *The effectiveness of secondary math teachers from Teach for America and the Teaching Fellow Programs.* NCEE 2013-4016. Washington, DC: U.S. Department of Education. | [Melissa](mailto:mclark@mathematica-mpr.com) Clark | **Yes** | Clark sent data. |
| 7 | Clark, M. A., Isenberg, E., Liu, A. Y., Makowsky, L., & Zukiewicz, M. (2016, March 4). *Impacts of the Teach For America Investing in Innovation Scale-Up.* Retrieved from http://mathematica-mpr.com | Melissa Clark | **Yes** | Clark sent data. |
| 8 | Darling-Hammond, L., Holtzman, D. J., Gatlin, S. J., & Heilig, J. V. (2005). Does teacher preparation matter? Evidence about teacher certification, Teach for America, and teacher effectiveness. *Education Policy Analysis Archives*, *13*(42). Retrieved from <http://epaa.asu.edu/epaa/v13n42/> | Linda Darling-Hammond | **No** | The Office of Linda-Darling Hammond sent a message saying that she is out of town. |

Table C.2 (continued)

| **No.** | **Study Reference** | **Primary Contact** | **Response** | **Explanation** |
| --- | --- | --- | --- | --- |
| 12 | Decker, P., Mayer, D., & Glazerman, S. (2004). *The Effects of Teach For America on Students: Findings from a National Evaluation.* Princeton, NJ: Mathematica Policy Research, Inc. | Steve Glazerman | Yes | - |
| 13 | Henry, G. T., Thompson, C. L., Bastian, K. C., Fortner, K. C., Kershaw, D. C., Purrell, K. M., & Zulli, R. A. (2010, June). *Portal report: Teacher preparation and student test scores in North Carolina.* Retrieved from <http://www.worldcat.org/title/portal-report-teacher-preparation-and-student-test-scores-in-north-carolina/oclc/789248583> | Gary Henry | **No** | – |
| 14 | Laczko-Kerr, I. I. (2002). *Teacher certification does matter: The effects of certification status on student achievement.* Unpublished doctoral dissertation, Arizona State University, Tempe. | Ildiko Laczko-Kerr | **Yes** | Confirmed that analysis was done using classroom-level data. |
| 15 | Noell, G., & Gansle K. (2009). *Teach for America teacher’s contribution to student achievement in Louisiana in grades 4–9: 2004–2005 to 2006–2007.* Baton Rouge, LA: Louisiana Board of Regents. | George Noell | **Yes** | Sent data as an Excel spreadsheet. |
| 16 | Pearson, J. L. (2014). *Effective instructional methods utilized in successful and high performing secondary schools in the Southern Region of Mississippi.* Unpublished doctoral dissertation, The University of Southern Mississippi, Hattiesburg. | Jane Pearson | **No** | – |
| 23 | Ware, A., LaTurner, R., Parsons, J., Okulicz-Kozaryn, A., Garland, M., & Klopfenstein, K. (2011). *Teacher Preparation Programs and Teach for America Research Study*. Dallas, TX: The University of Texas at Dallas Education Research Center. | Anne Ware | **Yes** | Ware requested copy of the report. Copy was sent. Ware reviewed but did not submit data. |
| 24 | Xu, Z., Hannaway, J., & Taylor, C. (2011). Making a difference? The effects of Teach for America in high school. *Journal of Policy Analysis and Management*, *30*(3), 447–469. | Zeyu Xu | **No** | Author query sent twice. |

Appendix D: Excluded studies and reasons

1. Antecol, H., Eren, O., & Ozbeklik, S. (2013). The effect of Teach for America on the distribution of student achievement in primary school: Evidence from a randomized experiment. *Economics of Education Review37*, 113­–125.

**Reason: Failed Stage 2 review.** Used same data as Decker, Mayer, and Glazerman (2004) but estimated effects of TFA at different quartiles of the outcome distribution. For estimating ATE, this study was excluded from the meta-analysis to avoid double-counting ATE.

1. Bastian, K. C. (2014). Selecting and preparing teachers and school leaders to improve educational outcomes. *Dissertation Abstracts International: The Humanities and Social Sciences.*

**Reason: Failed Stage 2 review.** Predictive study that uses value-added design. Author responded to query but could not provide the data needed to evaluate study as a QED, given the time frame.

1. Boyd, D., Grossman, P., Lankford, H., Loeb, S., & Wyckoff, J. (2006). *How changes in entry requirements alter the teacher workforce and affect student achievement*. Columbia, MO: American Education Finance Association. Retrieved from [https://cepa.stanford.edu/sites/default/files/Reducing Entry Requirements EPF 2006.pdf](https://cepa.stanford.edu/sites/default/files/Reducing%20Entry%20Requirements%20EPF%202006.pdf)

**Reason: Failed Stage 2 review**. Used value-added design. Author responded to query but could not provide the data needed to evaluate study as a QED, given the time frame.

1. Boyd, D., Grossman, P., Hammerness, K., Lankford, H., Loed, S., Ronfeldt, M., & Wyckoff, J. (2012). Recruiting effective math teachers: Evidence from New York City. *American Educational Research Journal*, *49*(6), 1008–1047.

**Reason: Failed Stage 2 review.** Used value-added design. Author responded to query but could not provide the data needed to evaluate study as a QED, given the time frame. Insufficient data to evaluate study as a QED.

1. Carroll, C. A. (2013). *The influence of Teach for America on Algebra I student achievement.* Unpublished doctoral dissertation, University of North Carolina, Charlotte.

**Reason: Failed Stage 2 review.** Insufficient data to establish baseline equivalence between groups in analysis sample.

1. Darling-Hammond, L., Holtzman, D. J., & Gatlin, S. J. (2005). Does teacher preparation matter? Evidence about teacher certification, Teach for America, and teacher effectiveness.*Education Policy Analysis Archives*,*13*(42), 1–47.

**Reason: Failed Stage 2 review.** Insufficient data to establish baseline equivalence between groups in the analysis sample.

1. Dee, T. S., & Wyckoff, J. (2015). Incentives, selection, and teacher performance: Evidence from IMPACT. *Journal of Policy Analysis and Management*, *34*(2), 267–297.

**Reason: Failed Stage 1 review.** Study did not examine the effects of TFA corps members or alumni.

1. Evans, B. R. (2009). First year middle and high school teachers’ mathematical content proficiency and attitudes: Alternative certification in the Teach for America (TFA) program. *Journal of the National Association for Alternative Certification*, *4*(1), 3–17.

**Reason: Failed Stage 1 review.** Correlational study design that is not an eligible design.

1. Evans, B. R. (2010). Determining quality teachers: Mathematical content knowledge, perceptions of teaching self-efficacy, and attitudes toward mathematics among a Teach for America cohort. *Journal of the National Association for Alternative Certification*, *5*(2), 23–35.

**Reason: Failed Stage 1 review.** Correlational study design that is not an eligible design.

1. Henry, G. T., Thompson, C. L., Bastian, K. C., Fortner, K. C., Kershaw, D. C., Purrell, K. M., & Zulli, R. A. (2010, June). *Portal report: Teacher preparation and student test scores in North Carolina.* Retrieved from <http://www.worldcat.org/title/portal-report-teacher-preparation-and-student-test-scores-in-north-carolina/oclc/789248583>

**Reason: Failed Stage 2 review.** Used teacher value-added design. Author did not respond to query. Data were not available to evaluate study as a QED. Insufficient data to evaluate study as a QED.

1. Laczko-Kerr, I. I. (2002). *Teacher certification does matter: The effects of certification status on student achievement.* Unpublished doctoral dissertation, Arizona State University, Tempe.

**Reason:** **Failed Stage 2 review.** Study makes causal inferences for student outcomes using classroom-level data. Student data were not available to establish baseline equivalence between groups, at the student level, in the analysis sample.

1. Noell, G. H., & Gansle, K. A. (2009). *Teach For America teachers’ contribution to student achievement in Louisiana in grades 4–9: 2004–2005 to 2006–2007.* Baton Rouge, LA: Louisiana Board of Regents.

**Reason: Failed Stage 2 review.** Pretest data in the analysis sample were imputed. Therefore, we could not establish baseline equivalence between groups in analysis sample.

1. Pearson, J. L. (2014). *Effective instructional methods utilized in successful and high performing secondary schools in the Southern Region of Mississippi.* Unpublished doctoral dissertation, The University of Southern Mississippi, Hattiesburg.

**Reason: Failed Stage 2 review.** Used value-added design. Author responded to query but could not provide data needed to evaluate study as a QED, given the time frame.

1. Penner, E. K. (2014). *Teaching for all? Variation in the effects of Teach for America.* Unpublished doctoral dissertation, University of California, Irvine.

**Reason: Failed Stage 2 review.** Could not establish equivalence between groups because researchers imputed data in the analysis sample. For this review, baseline equivalence can be established using only non-imputed data.

1. Prescott, S. H. (2010). *The effects of affirmative quality feedback on low socio-economic students’ zone of proximal development reading gains (ZPDRL): A causal-comparative study.* Unpublished doctoral dissertation, The University of Mississippi, Oxford.

**Reason: Failed Stage 1 review.** TFA was delivered for only one semester and not the full school year (as required in the protocol).

1. Raymond, M., & Fletcher, S. (2002, Spring). The Teach for America Evaluation: Herewith, the first evidence on its recruits’ actual performance in the classroom. *Education Next*, 62–68.

Raymond, M., Fletcher, S., & Luque, J. (2001). *Teach for America: An evaluation of teacher differences and student outcomes in Houston, Texas*. Stanford, CA: The Center for Research on Education Outcomes.

**Reason: Failed Stage 2 review.** Insufficient data to establish baseline equivalence between groups in analysis sample.

1. Ready, D. D. (2014). *Teach for America Teachers in Duval County Public Schools: An Analysis of Retention and Performance.* Retrieved from <https://www.tc.columbia.edu/faculty/ddr2111/facultyprofile/files/FINAL_TFA_DUVAL.pdf>

**Reason: Failed Stage 2 review.** Insufficient data to establish baseline equivalence between groups in analysis sample.

1. Tennessee Higher Education Commission. (2010). *Report Card on the Effectiveness of Teacher Training Programs.* Nashville, TN: State Board of Education and Tennessee Higher Education Commission.

**Reason: Failed Stage 2 review.** Used teacher value-added design. Did not respond to author query. Insufficient data to evaluate study as a QED.

1. Ware, A., LaTurner, J. R., Parsons, J., Okulicz-Kozaryn, A., Garland, M., & Klopfenstein, K. (2011). *Teacher Preparation Programs and Teach for America Research Study* (Rep.). Retrieved from <https://www.researchgate.net/publication/236333015_Evaluation_of_Teach_For_America_in_Texas_Schools>

**Reason: Lacked contrasts comparable to other studies.** Study reported contrasts that “Meet Evidence Criteria” *with reservations*, but none of the other included studies reported similar contrasts that pooled either elementary or middle school grade levels. Moreover, none of the other included studies used the Non-TFA novice comparison at the high school level.

1. Xu, Z., Hannaway, J., Taylor, C., & Urban Institute, National Center for Analysis of Longitudinal Data in Education Research. (2009). *Making a difference? The effects of Teach for America in high school. Working paper 17. Revised.* Washington, DC: National Center for Analysis of Longitudinal Data in Education Research.

Xu, Z., Hannaway, J., & Taylor, C. (2011). Making a difference? The effects of Teach for America in high school. *Journal of Policy Analysis and Management*, *30*(3), 447–469.

**Reason: Failed Stage 1 review.** Insufficient data to establish baseline equivalence between groups in the analysis sample.

Appendix E: Contrasts reported in included studies

Table E.1. *Contrasts Reported for TFA Studies that Passed Stage 1 or Stage 2 Screening*

| **Study** | **Contrasts** | **Grade** | **Outcome** | **Met Evidence Criteria** |
| --- | --- | --- | --- | --- |
|  |  |  |  |  |
| Decker, Mayer, | First Year TFA vs. Non-TFA | Elementary | Math | CNBE^1^ |
|  | First Year TFA vs. Non-TFA | Elementary | Reading | CNBE |
| & Glazerman, | Second Year and Veteran TFA vs. Non-TFA | Elementary | Math | CNBE |
| 2004 | Second Year and Veteran TFA vs. Non-TFA | Elementary | Reading | CNBE |
|  | TFA vs. Non-TFA | Elementary | Math | Yes |
|  | TFA vs. Non-TFA | Elementary | Reading | Yes |
|  | TFA vs. Non-TFA Certified | Elementary | Math | CNBE |
|  | TFA vs. Non-TFA Certified | Elementary | Reading | CNBE |
|  | TFA vs. Non-TFA Uncertified | Elementary | Math | CNBE |
|  | TFA vs. Non-TFA Uncertified | Elementary | Reading | CNBE |
|  | TFA vs. Non-TFA Veteran | Elementary | Math | CNBE |
|  | TFA vs. Non-TFA Veteran | Elementary | Reading | CNBE |
| Clark et al., | TFA Corps vs. Non-TFA: Grades 3 to 5 | Elementary | Math | Yes |
| 2015 | TFA Corps vs. Non-TFA: Grades 3 to 5 | Elementary | Reading | Yes |
|  | TFA Corps vs. Non-TFA: Grades PreK to 2 | Elementary | Math | Yes |
|  | TFA Corps vs. Non-TFA: Grades PreK to 2 | Elementary | Reading | Yes |
|  | TFA Corps vs. Non-TFA: Grades PreK to 5 | Elementary | Math | Yes |
|  | TFA Corps vs. Non-TFA: Grades PreK to 5 | Elementary | Reading | Yes |
|  | TFA Corps vs. Non-TFA: Grades PreK to K | Elementary | Math | Yes |
|  | TFA Corps vs. Non-TFA: Grades PreK to K | Elementary | Reading | Yes |
|  | TFA Corps vs. Non-TFA, Trad.^2^ Certified | Elementary | Math | Yes |
|  | TFA Corps vs. Non-TFA, Trad. Certified | Elementary | Reading | Yes |
|  | TFA Corps Novice vs. Non-TFA: Grades PreK to 5 | Elementary | Math | Yes |

Table E.1 (continued)

| **Study** |  | **Comparison** | **Grade** | **Outcome** | **Meet Evidence Criteria** |
| --- | --- | --- | --- | --- | --- |
|  |  | TFA Corps Novice vs. Non-TFA: Grades PreK to 5) | Elementary | Reading | Yes |
| Clark et al., 2013 |  | First Year TFA vs. Non-TFA, Experienced | Middle and High | Math | Yes |
|  |  | Second Year TFA vs. Non-TFA, Experienced | Middle and High | Math | Yes |
|  |  | TFA Corps vs. Non-TFA Experienced | Middle and High | Math | Yes |
|  |  | TFA Corps vs. Non-TFA Novice Teachers | Middle and High | Math | Yes |
|  |  | TFA vs. Non-TFA | Middle and High | Math | Yes |
|  |  | TFA vs. Non-TFA: High School | High | Math | Yes |
|  |  | TFA vs. Non-TFA: Middle School | Middle | Math | Yes |
|  |  | TFA vs. Non-TFA Alternative Route | Middle and High | Math | Yes |
|  |  | TFA vs. Non-TFA Trad. Route | Middle and High | Math | Yes |
| Turner et al., 2012 |  | TFA Corps vs. Non-TFA, Novice | Middle | Math | Yes, WRS^3^ |
|  |  | TFA Corps vs. Non-TFA, Novice | Middle | Reading | Yes, WRS |
|  |  | TFA Alumni vs. Non-TFA, Veteran | Middle | Math | Yes, WRS |
|  |  | TFA Alumni vs. Non-TFA, Veteran | Middle | Reading | Yes, WRS |

^1^ CNBE = Could Not Be Evaluated (because the information for the comparison was not reported).

^2^ Trad. = traditional.

^3^ WRS = With Reservations.

Appendix F: Risk of bias

Table F.1. *Assessing Risk of Bias in Primary Studies for TFA Systematic Review*

| **Criteria** | **Description** |
| --- | --- |
| ***Screening*** | *To be included in the design quality review, a study must have all the characteristics listed below.* |
| Focus | The study must focus on the effectiveness of the TFA intervention. |
| Time | The study must be published or reported between 1995 and the present. |
| Age | The study must focus on students in grades K–12. |
| Location | TFA must be implemented in the United States. |
| Outcome | The study must report at least one student academic outcome in math, English language arts, or science. |
| Exposure | Students in the TFA group must have at least one school year of exposure to the TFA corps member or alumni before outcome measurement. |
| Setting | The study of TFA must take place in a U.S. public or charter school. |
| Design | The TFA and counterfactual groups must be formed with random assignment or quasi-experimental methods. |
| ***Design Quality*** | *To be included in the statistical synthesis, a contrast must satisfy all the criteria listed below.* |
| Bundled Treatment Group Confound | To qualify as a treatment group, all treatment teachers must be TFA corps members or TFA alumni. |
| Bundled Intervention Confound | The TFA intervention must *not* be completely aligned with another intervention, such as another alternative route teacher preparation program. |
| *N* = 1 Confound | Each condition required more than one unit at each level (student, teacher, school, district, state, and so on). If there was only one unit at a level, the unit was controlled for across groups. |
| Outcome Face Validity | A description of the outcome must provide evidence that the measure is well-defined, is interpretable, and measures what it is purported to measure. |
| Outcome Reliability | Measures must demonstrate an internal consistency reliability of 0.50, inter-rater reliability of 0.50, or temporal stability reliability of 0.40. |
| Outcome Alignment | Outcomes must not be over-aligned with the intervention. |
| Outcome Measure Confound | Outcomes must be measured in the same way for both conditions. |
| Standardized Outcomes | Standardized outcomes are assumed to satisfy the face validity, reliability, alignment, and measurement confound criteria. |
| Attrition | RCTs must demonstrate low attrition; otherwise, a baseline equivalence test is required. Attrition is considered high if the combination between overall and differential attrition exceeds the thresholds defined by WWC’s liberal attrition standard. |
| Cluster Attrition | Cluster RCTs must test for high attrition at the cluster level. In addition, they must test for high attrition at the sub-cluster level using the clusters with outcome data. |

Table F.1 (continued)

| **Criteria** | **Description** |
| --- | --- |
| ***Design Quality* (cont.)** | *To be included in the statistical synthesis, a contrast must satisfy all the criteria listed below.* |
| Baseline Equivalence | All QEDs and RCTs with high attrition must use a pre-intervention measure of the outcome to show evidence of baseline equivalence on the analysis sample.  • If the absolute value of the effect size is less than or equal to 0.05, the groups are considered equivalent.  • If the absolute value of the effect size is greater than 0.05 and less than or equal to 0.25, the analysis model must statistically adjust for the pre-intervention measure.  • If the absolute value of the effect size is greater than 0.25, the outcome is not eligible for the statistical synthesis. |
| All studies that satisfied the above criteria and had sufficient outcome date were eligible for inclusion in the statistical synthesis and assigned a study rating as follows:   - RCTs with no confounds, reliable outcomes, and low attrition received a rating of meets design quality standards without reservations. - RCTs with high attrition and QEDs with no confounds, reliable outcomes, and baseline group equivalence in analysis samples received a rating of meets design quality standards with reservations. | |
| ***Risk of Bias Conduct*** | *The following describes how this review complied with the* Adaptations on MECIR [Methodological Expectations of Campbell Collaboration Intervention Reviews] Version 2.2 Reporting Standards *on issues related to risk of bias.* |
| Assessing Risk of Bias/Study Quality | The risk of bias was assessed for all RCTs and QEDs that passed screening, using the design criteria outlined in the *WWC Procedures and Standards Handbook, Version 3.0*. The specific criteria are outlined above, under Design Quality. |
| Assessing Risk of Bias/Study Quality in Duplicate | All studies that passed the initial title and abstract screen were double-coded by trained coders. |
| Supporting Judgments of Risk of Bias/Study Quality | Coders used information directly from the study to justify all decisions. |
| Providing Sources of Information for Risk of Bias/Study Quality Assessments | Coders collected and documented the source of information for each study quality assessment. They clearly documented what information comes directly from the report, what information was obtained from the author query, and what assumptions were made. |
| Differentiating Between Performance Bias and Detection Bias | Selection bias and attrition bias were assessed through our use of WWC Evidence Standards for Group Design, Version 3.0, with the former assessed through how groups are formed and the latter assessed for RCTs based on whether there is high attrition. Detection bias and reporting bias were assessed based on whether the authors reported all outcomes for which data were collected or only reported a subset (without justification). |

Table F.1 (continued)

| **Criteria** | **Description** |
| --- | --- |
| ***Risk of Bias Conduct* (cont.)** | *The following describes how this review complied with the* Adaptations on MECIR Version 2.2 Reporting Standards *on issues related to risk of bias.* |
| If Applicable, Assessing Risk of Bias Due to Lack of Blinding for Different Outcomes | This assessment is rare in TFA studies in particular and in education research studies in general because “concealment” of the intervention condition from the investigator or study participants or both, is rarely feasible or practical. |
| If Applicable, Assessing Completeness of Data for Different Outcomes | Within a study, completeness of data may be handled differently for different contrasts. RCTs with low attrition may use the missing data techniques outlined in the WWC *Procedures and Standards Handbook, Version 3.0*. |
| If Applicable, Summarizing Risk of Bias Assessments When Using the Cochrane Risk of Bias Tool | Not applicable because the Cochrane Risk of Bias Tool was not used. |
| Addressing Risk of Bias/Study Quality in the Synthesis | This review originally planned to use sensitivity analysis to check if the results were sensitive to the inclusion or exclusion of particular studies using a “one study removed” analysis. There were not enough studies to carry out this analysis. |
| Incorporating Assessments of Risk of Bias | Within each study, each contrast (intervention vs. comparison group on each outcome) was assigned a quality rating (see design quality row above) that incorporates the risk of bias. |
| ***Risk of Bias Reporting*** | *The following describes how this review complied with the* Adaptations on MECIR Version 2.2 Reporting Standards *on issues related to risk of bias.* |
| Risk of Bias and/or Study Quality Table | After assessing all design quality issues, the SCG required coders to select a study rating and a study disposition code (with an explanation) as follows:   - RCTs with no confounds, reliable outcomes, and low attrition received a rating of *meets design quality standards without reservations*. - RCTs with high attrition and QEDs with no confounds, reliable outcomes, and baseline groups equivalence in analysis samples “received a rating of *meets design quality standards with reservations*. |
| Summary Assessments of Risk of Bias/Study Quality |  |
| Study Quality/Risk of Bias in Included Studies |  |
