# Supplementary material for: What are the effects of Teach For America on Math, English Language Arts, and Science outcomes of K–12 students in the USA?
Source: Campbell Syst Rev. 2018 Jun 25;14(1):1–60. doi: 10.4073/csr.2018.7 (PMC8428006; doi:10.4073/csr.2018.7)
Supplement: Supplementary file 2 — Supplementary material [file CL2-14--s001.pdf]

Online supplements

Appendix A.1: Results from electronic database searches

ERIC (EBSCO)

- *Search 1:* (TI [“Teach for America”] OR AB [“Teach for America” OR “TFA Corps”])
  - Number of Articles: 159
  - Source Type: Academic Journals (64), ERIC Documents (59), Magazines (36), Educational Reports (7), Books (1)
- *Search 2:* (TI [“Teach for America”] OR AB [“Teach for America” OR “TFA Corps”]) **AND (“academic achievement” OR “success*” OR “grade level” OR “grading” OR “academic ability” OR “attainment” OR “failure” OR “educational indicator*”)**
  - Number of Articles: 99
  - Source Type: Academic Journals (41), ERIC Documents (42), Magazines (16), Educational Reports (4)
- *Search 3:* (TI [“Teach for America”] OR AB [“Teach for America” OR “TFA Corps”]) AND (“academic achievement” OR “success*” OR “grade level” OR “grading” OR “academic ability” OR “attainment” OR “failure” OR “educational indicator*”) **AND (“kindergarten” OR “elementary school*” OR “primary school*” OR “high school*” OR “public school”)**
  - Number of Articles: 74
  - Source Type: Academic Journals (36), ERIC Documents (29), Magazines (9), Educational Reports (1)
- *Search 4:* (TI [“Teach for America”] OR AB [“Teach for America” OR “TFA Corps”]) AND (“academic achievement” OR “success*” OR “grade level” OR “grading” OR “academic ability” OR “attainment” OR “failure” OR “educational indicator*”) AND (“kindergarten” OR “elementary school*” OR “primary school*” OR “high school*” OR “public school”) **AND (“random assignment” OR “randomized experiment” OR “experiment*” OR “experimental design” OR “control group” OR “non-experiment” OR “non-experimental” OR “quasi-experiment” OR “quasi-experimental” OR “comparison group” OR “matched comparison group” OR “matched comparison” OR “matched groups” OR “statistical matching” OR “propensity score matching” OR “systematic review” OR “review” OR “meta-analysis” OR “research synthesis” OR “research review”)**
  - Number of Articles: 69
  - Source Type: Academic Journals (32), ERIC Documents (27), Magazines (10), Educational Reports (1)
- *Search 5:* Changing AND (“kindergarten” OR “elementary school*” OR “primary school*” OR “high school*” OR “public school”) to **NOT** changes the count to **67**.

Seven ERIC references passed initial screening

1. Clark, M. A., Chiang, H. S., Silva, T., McConnell, S., Sonnenfeld, K., Erbe, A., & Puma, M. (2013). *The effectiveness of secondary math teachers from Teach for America and the teaching fellows programs. NCEE 2013-4015.* Jessup, MD: National Center for Education Evaluation and Regional Assistance.

2. Evans, B. R. (2009). First year middle and high school teachers' mathematical content proficiency and attitudes: Alternative certification in the Teach for America (TFA) program. *Journal of the National Association for Alternative Certification*, *4*(1), 3–17.

3. Evans, B. R. (2010). Determining quality teachers: Mathematical content knowledge, perceptions of teaching self-efficacy, and attitudes toward mathematics among a Teach for America cohort. *Journal of the National Association for Alternative Certification*, *5*(2), 23–35.

4. Glazerman, S., Mayer, D., & Decker, P. (2006). Alternative routes to teaching: The impacts of Teach for America on student achievement and other outcomes. *Journal of Policy Analysis and Management*, *25*(1), 75–96.

5. Heilig, J. V., & Jez, S. J. (2010). *Teach for America: A review of the evidence.* Boulder and Tempe, AZ: Education and the Public Interest Center & Education Policy Research Unit.

6. Xu, Z., Hannaway, J., & Taylor, C. (2011). Making a difference? The effects of Teach for America in high school. *Journal of Policy Analysis and Management*, *30*(3), 447–469.

7. Xu, Z., Hannaway, J., Taylor, C., & Urban Institute, National Center for Analysis of Longitudinal Data in Education Research. (2009). *Making a difference? The effects of Teach for America in high school. Working paper 17. Revised.* Washington, DC: National Center for Analysis of Longitudinal Data in Education Research.

PsycINFO (ProQuest)

- *Search 1:* (TI ["Teach for America"] OR AB ["Teach for America" OR "TFA Corps"])
  - Number of Articles: 42
  - Source Type: Scholarly Journals (21), Dissertations and Theses (19), Book (2)
- *Search 2:* (TI ["Teach for America"] OR AB ["Teach for America" OR "TFA Corps"]) **AND ("academic achievement" OR "success*" OR "grade level" OR "grading" OR "academic ability" OR "attainment" OR "failure" OR "educational indicator*")**
  - Number of Articles: 29
  - Source Type: Scholarly Journals (15), Dissertations and Theses (13), Book (1)
- *Search 3:* (TI ["Teach for America"] OR AB ["Teach for America" OR "TFA Corps"]) AND ("academic achievement" OR "success*" OR "grade level" OR "grading" OR "academic ability" OR "attainment" OR "failure" OR "educational indicator*") **AND ("kindergarten" OR "elementary school*" OR "primary school*" OR "high school*" OR "public school")**
  - Number of Articles: 25
  - Source Type: Scholarly Journals (14), Dissertations and Theses (10), Book (1)
- *Search 4:* (TI ["Teach for America"] OR AB ["Teach for America" OR "TFA Corps"]) AND ("academic achievement" OR "success*" OR "grade level" OR "grading" OR "academic ability" OR "attainment" OR "failure" OR "educational indicator*") AND ("kindergarten" OR "elementary school*" OR "primary school*" OR "high school*" OR "public school") **AND ("random assignment" OR "randomized experiment" OR "experiment*" OR "experimental design" OR "control group" OR "non-experiment" OR "non-experimental" OR "quasi-experiment" OR "quasi-experimental" OR "comparison group" OR "matched comparison group" OR "matched comparison" OR "matched**

groups" OR "statistical matching" OR "propensity score matching" OR "systematic review" OR "review" OR "meta-analysis" OR "research synthesis" OR "research review")

  - Number of Articles: 26
  - Source Type: Scholarly Journals (15), Dissertations and Theses (10), Book (1)

- *Search 5:* Changing AND ("kindergarten" OR "elementary school*" OR "primary school*" OR "high school*" OR "public school") to **NOT** changes the count to **27**.

Two PsycINFO references passed initial screening

1. Glazerman, S., Mayer, D., & Decker, P. (2006). Alternative routes to teaching: The impacts of Teach for America on student achievement and other outcomes. *Journal of Policy Analysis and Management, 25*(1), 75–96.
2. Xu, Z., Hannaway, J., & Taylor, C. (2011). Making a difference? The effects of Teach for America in high school. *Journal of Policy Analysis and Management, 30*(3), 44–469.

EconLit (ProQuest)

- *Search 1:* (TI ["Teach for America"] OR AB ["Teach for America" OR "TFA Corps"])
  - Number of Articles: 4
  - Source Type: Scholarly Journals (2), Working Paper (1)
- *Search 2:* (TI ["Teach for America"] OR AB ["Teach for America" OR "TFA Corps"]) **AND ("academic achievement" OR "success*" OR "grade level" OR "grading" OR "academic ability" OR "attainment" OR "failure" OR "educational indicator*")**
  - Number of Articles: 0
- Search 3: (TI ["Teach for America"] OR AB ["Teach for America" OR "TFA Corps"]) AND ("academic achievement" OR "success*" OR "grade level" OR "grading" OR "academic ability" OR "attainment" OR "failure" OR "educational indicator*") **AND ("kindergarten" OR "elementary school*" OR "primary school*" OR "high school*" OR "public school")**
  - Number of Articles: 1
- *Search 4:* (TI ["Teach for America"] OR AB ["Teach for America" OR "TFA Corps"]) AND ("academic achievement" OR "success*" OR "grade level" OR "grading" OR "academic ability" OR "attainment" OR "failure" OR "educational indicator*") AND ("kindergarten" OR "elementary school*" OR "primary school*" OR "high school*" OR "public school") **AND ("random assignment" OR "randomized experiment" OR "experiment*" OR "experimental design" OR "control group" OR "non-experiment" OR "non-experimental" OR "quasi-experiment" OR "quasi-experimental" OR "comparison group" OR "matched comparison group" OR "matched comparison" OR "matched groups" OR "statistical matching" OR "propensity score matching" OR "systematic review" OR "review" OR "meta-analysis" OR "research synthesis" OR "research review")**
  - Number of Articles: 0
- *Search 5:* (TI ["Teach for America"] OR AB ["Teach for America" OR "TFA Corps"]) **AND ("random assignment" OR "randomized experiment" OR "experiment*"** OR "experimental design" OR "control group" OR "non-experiment" OR "non-experimental" OR "quasi-experiment" OR "quasi-experimental" OR "comparison group" OR "matched comparison group" OR "matched comparison" OR "matched groups" OR "statistical matching" OR "propensity score matching" OR "systematic review" OR "review" OR "meta-analysis" OR "research synthesis" OR "research review")
  - Number of Articles: 5
  - Source Type: Scholarly Journals (4), Working Paper (1)

Three EconLit references passed initial screening

1. Carroll, C. A. (2013). *The influence of Teach for America on Algebra I student achievement.* Unpublished doctoral dissertation, University of North Carolina, Charlotte.
2. Glazerman, S., Mayer, D., & Decker, P. (2006). Alternative routes to teaching: The impacts of Teach for America on student achievement and other outcomes. *Journal of Policy Analysis and Management, 25*(1), 75–96.
3. Xu, Z., Hannaway, J., & Taylor, C. (2011). Making a difference? The effects of Teach for America in high school. *Journal of Policy Analysis and Management, 30*(3), 447–469.

Sociological Abstracts (ProQuest)

- *Search 1:* (TI ["Teach for America"] OR AB ["Teach for America" OR "TFA Corps"])
  - Number of Articles: 16
  - Source Type: Dissertations and Theses (9), Scholarly Journals (7)
- *Search 2:* (TI ["Teach for America"] OR AB ["Teach for America" OR "TFA Corps"]) **AND ("academic achievement" OR "success*" OR "grade level" OR "grading" OR "academic ability" OR "attainment" OR "failure" OR "educational indicator*")**
  - Number of Articles: 12
  - Source Type: Dissertations and Theses (8), Scholarly Journals (4)
- *Search 3:* (TI ["Teach for America"] OR AB ["Teach for America" OR "TFA Corps"]) AND ("academic achievement" OR "success*" OR "grade level" OR "grading" OR "academic ability" OR "attainment" OR "failure" OR "educational indicator*") **AND ("kindergarten" OR "elementary school*" OR "primary school*" OR "high school*" OR "public school")**
  - Number of Articles: 10
  - Source Type: Dissertations and Theses (6), Scholarly Journals (4)
- *Search 4:* (TI ["Teach for America"] OR AB ["Teach for America" OR "TFA Corps"]) AND ("academic achievement" OR "success*" OR "grade level" OR "grading" OR "academic ability" OR "attainment" OR "failure" OR "educational indicator*") AND ("kindergarten" OR "elementary school*" OR "primary school*" OR "high school*" OR "public school") **AND ("random assignment" OR "randomized experiment" OR "experiment*" OR "experimental design" OR "control group" OR "non-experiment" OR "non-experimental" OR "quasi-experiment" OR "quasi-experimental" OR "comparison group" OR "matched comparison group" OR "matched comparison" OR "matched groups" OR "statistical matching" OR "propensity score matching" OR "systematic review" OR "review" OR "meta-analysis" OR "research synthesis" OR "research review")**
  - Number of Articles: 9
  - Source Type: Dissertations and Theses (5), Scholarly Journals (4)

Two Sociological Abstracts references passed initial screening

1. Bastian, K. C. (2014). *Selecting and preparing teachers and school leaders to improve educational outcomes*. Ann Arbor, MI: ProQuest Information & Learning. AAI3562689.
2. Prescott, S. H. (2011). *The effects of affirmative quality feedback on low socio-economic students' zone of proximal development reading gains (ZPDRL): A causal-comparative study.* Ann Arbor, MI: ProQuest Information & Learning. AAI3447103.

Database: PAIS International (ProQuest)

- *Search 1:* (TI ["Teach for America"] OR AB ["Teach for America" OR "TFA Corps"])

- Number of Articles: 14
- Source Type: Scholarly Journals (8), Books (6)

- *Search 2:* (TI ["Teach for America"] OR AB ["Teach for America" OR "TFA Corps"]) **AND ("academic achievement" OR "success*" OR "grade level" OR "grading" OR "academic ability" OR "attainment" OR "failure" OR "educational indicator*")**
  - Number of Articles: 11
  - Source Type: Scholarly Journals (6), Books (5)
- *Search 3:* (TI ["Teach for America"] OR AB ["Teach for America" OR "TFA Corps"]) AND ("academic achievement" OR "success*" OR "grade level" OR "grading" OR "academic ability" OR "attainment" OR "failure" OR "educational indicator*") **AND ("kindergarten" OR "elementary school*" OR "primary school*" OR "high school*" OR "public school")**
  - Number of Articles: 11
  - Source Type: Scholarly Journals (6), Books (5)
- *Search 4:* (TI ["Teach for America"] OR AB ["Teach for America" OR "TFA Corps"]) AND ("academic achievement" OR "success*" OR "grade level" OR "grading" OR "academic ability" OR "attainment" OR "failure" OR "educational indicator*") AND ("kindergarten" OR "elementary school*" OR "primary school*" OR "high school*" OR "public school") **AND ("random assignment" OR "randomized experiment" OR "experiment*" OR "experimental design" OR "control group" OR "non-experiment" OR "non-experimental" OR "quasi-experiment" OR "quasi-experimental" OR "comparison group" OR "matched comparison group" OR "matched comparison" OR "matched groups" OR "statistical matching" OR "propensity score matching" OR "systematic review" OR "review" OR "meta-analysis" OR "research synthesis" OR "research review")**
  - Number of Articles: 11
  - Source Type: Scholarly Journals (6), Books (5)

Three PAIS International references passed initial screening

1. Antecol, H., Eren, O., & Ozbeklik, S. (2013). The effect of Teach for America on the distribution of student achievement in primary school: Evidence from a randomized experiment. *Economics of Education Review*, *37*, 113–125.
2. Glazerman, S., Mayer, D., & Decker, P. (2006). Alternative routes to teaching: The impacts of Teach for America on student achievement and other outcomes. *Journal of Policy Analysis and Management*, *25*(1), 75–96.
3. Xu, Z., Hannaway, J., & Taylor, C. (2011). Making a difference? The effects of Teach for America in high school. *Journal of Policy Analysis and Management*, *30*(3), 447–469.

ProQuest Dissertations and Theses, UK and Ireland

- *Search 1:* (TI ["Teach for America"] OR AB ["Teach for America" OR "TFA Corps"])
  - Number of Articles: 1
  - Source Type: Dissertation and Theses
- *Search 2:* (TI ["Teach for America"] OR AB ["Teach for America" OR "TFA Corps"]) **AND ("academic achievement" OR "success*" OR "grade level" OR "grading" OR "academic ability" OR "attainment" OR "failure" OR "educational indicator*")**
  - Number of Articles: 1
  - Source Type: Dissertation and Theses
- *Search 3:* (TI ["Teach for America"] OR AB ["Teach for America" OR "TFA Corps"]) AND ("academic achievement" OR "success*" OR "grade level" OR "grading" OR "academic ability"

OR "attainment" OR "failure" OR "educational indicator*") **AND ("kindergarten" OR "elementary school*" OR "primary school*" OR "high school*" OR "public school")**

  - Number of Articles: 1
  - Source Type: Dissertation and Theses

- *Search 4:* (TI ["Teach for America"] OR AB ["Teach for America" OR "TFA Corps"]) AND ("academic achievement" OR "success*" OR "grade level" OR "grading" OR "academic ability" OR "attainment" OR "failure" OR "educational indicator*") AND ("kindergarten" OR "elementary school*" OR "primary school*" OR "high school*" OR "public school") **AND ("random assignment" OR "randomized experiment" OR "experiment*" OR "experimental design" OR "control group" OR "non-experiment" OR "non-experimental" OR "quasi-experiment" OR "quasi-experimental" OR "comparison group" OR "matched comparison group" OR "matched comparison" OR "matched groups" OR "statistical matching" OR "propensity score matching" OR "systematic review" OR "review" OR "meta-analysis" OR "research synthesis" OR "research review")**
  - Number of Articles: 1
  - Source Type: Dissertation and Theses

No references in ProQuest Dissertations and Theses, UK and Ireland, passed initial screening.

ProQuest Dissertations and Theses Global

- *Search 1:* (TI ["Teach for America"] OR AB ["Teach for America" OR "TFA Corps"])
  - Number of Articles: 54; doctoral dissertation only (48)
  - Source Type: Dissertation and Theses
- *Search 2:* (TI ["Teach for America"] OR AB ["Teach for America" OR "TFA Corps"]) **AND ("academic achievement" OR "success*" OR "grade level" OR "grading" OR "academic ability" OR "attainment" OR "failure" OR "educational indicator*")**
  - Number of Articles: 52; doctoral dissertation only (46)
  - Source Type: Dissertation and Theses
- *Search 3:* (TI ["Teach for America"] OR AB ["Teach for America" OR "TFA Corps"]) AND ("academic achievement" OR "success*" OR "grade level" OR "grading" OR "academic ability" OR "attainment" OR "failure" OR "educational indicator*") **AND ("kindergarten" OR "elementary school*" OR "primary school*" OR "high school*" OR "public school")**
  - Number of Articles: 51; doctoral dissertation only (45)
  - Source Type: Dissertation and Theses
- *Search 4:* (TI ["Teach for America"] OR AB ["Teach for America" OR "TFA Corps"]) AND ("academic achievement" OR "success*" OR "grade level" OR "grading" OR "academic ability" OR "attainment" OR "failure" OR "educational indicator*") AND ("kindergarten" OR "elementary school*" OR "primary school*" OR "high school*" OR "public school") **AND ("random assignment" OR "randomized experiment" OR "experiment*" OR "experimental design" OR "control group" OR "non-experiment" OR "non-experimental" OR "quasi-experiment" OR "quasi-experimental" OR "comparison group" OR "matched comparison group" OR "matched comparison" OR "matched groups" OR "statistical matching" OR "propensity score matching" OR "systematic review" OR "review" OR "meta-analysis" OR "research synthesis" OR "research review")**
  - Number of Articles: 51; doctoral dissertation only (45)
  - Source Type: Dissertation and Theses

Five ProQuest Dissertations and Theses Global References passed initial screening:

1. Carroll, C. A. (2013). *The influence of Teach for America on Algebra I student achievement.* Unpublished doctoral dissertation, University of North Carolina, Charlotte.
2. Laczko-Kerr, I. I. (2002). *Teacher certification does matter: The effects of certification status on student achievement.* Unpublished doctoral dissertation, Arizona State University, Tempe.
3. Pearson, J. L. (2014). *Effective instructional methods utilized in successful and high performing secondary schools in the Southern Region of Mississippi.* Unpublished doctoral dissertation, The University of Southern Mississippi, Hattiesburg.
4. Penner, E. K. (2014). *Teaching for all? Variation in the effects of Teach for America.* Unpublished doctoral dissertation, University of California, Irvine.
5. Prescott, S. H. (2010). The effects of affirmative quality feedback on low socio-economic students' zone of proximal development reading gains (ZPDRL): A causal-comparative study. Unpublished doctoral dissertation, University of Mississippi, Oxford.

Database: Worldwide Political Science Abstracts

- *Search 1:* (TI ["Teach for America"] OR AB ["Teach for America" OR "TFA Corps"])
  - Number of Articles: 2
  - Source Type: Scholarly Journal (1) Dissertation and Theses (1)
- *Search 2:* (TI ["Teach for America"] OR AB ["Teach for America" OR "TFA Corps"]) **AND ("academic achievement" OR "success*" OR "grade level" OR "grading" OR "academic ability" OR "attainment" OR "failure" OR "educational indicator*")**
  - Number of Articles: 1
  - Source Type: Scholarly Journal (1)
- *Search 3:* (TI ["Teach for America"] OR AB ["Teach for America" OR "TFA Corps"]) AND ("academic achievement" OR "success*" OR "grade level" OR "grading" OR "academic ability" OR "attainment" OR "failure" OR "educational indicator*") **AND ("kindergarten" OR "elementary school*" OR "primary school*" OR "high school*" OR "public school")**
  - Number of Articles: 1
  - Source Type: Scholarly Journal (1)
- *Search 4:* (TI ["Teach for America"] OR AB ["Teach for America" OR "TFA Corps"]) AND ("academic achievement" OR "success*" OR "grade level" OR "grading" OR "academic ability" OR "attainment" OR "failure" OR "educational indicator*") AND ("kindergarten" OR "elementary school*" OR "primary school*" OR "high school*" OR "public school") **AND ("random assignment" OR "randomized experiment" OR "experiment*" OR "experimental design" OR "control group" OR "non-experiment" OR "non-experimental" OR "quasi-experiment" OR "quasi-experimental" OR "comparison group" OR "matched comparison group" OR "matched comparison" OR "matched groups" OR "statistical matching" OR "propensity score matching" OR "systematic review" OR "review" OR "meta-analysis" OR "research synthesis" OR "research review")**
  - Number of Articles: 1
  - Source Type: Scholarly Journal (1)

No references in Worldwide Political Science Abstracts passed initial screening.

Database: JSTOR

- *Search 1:* (TI ["Teach for America"] OR AB ["Teach for America" OR "TFA Corps"])
  - Number of Articles: 18

- Source Type: Scholarly Journals

- *Search 2:* (TI ["Teach for America"] OR AB ["Teach for America" OR "TFA Corps"]) **AND ("academic achievement" OR "success*" OR "grade level" OR "grading" OR "academic ability" OR "attainment" OR "failure" OR "educational indicator*")**
  - Number of Articles: 8
  - Source Type: Scholarly Journals

  Search Note: Adding the search terms in bold made the search strategy too long and ineffective. Suggestion: Use **AND ("academic achievement" OR "failure")**.

- *Search 3:* (TI ["Teach for America"] OR AB ["Teach for America" OR "TFA Corps"]) AND ("academic achievement" OR "success*" OR "grade level" OR "grading" OR "academic ability" OR "attainment" OR "failure" OR "educational indicator*") **AND ("kindergarten" OR "elementary school*" OR "primary school*" OR "high school*" OR "public school")**

  Search Note: Adding the search terms in bold made the search strategy too long and ineffective. As a result, these terms were not added, and this search strategy was not used with this database.

- *Search 4:* (TI ["Teach for America"] OR AB ["Teach for America" OR "TFA Corps"]) AND ("academic achievement" OR "success*" OR "grade level" OR "grading" OR "academic ability" OR "attainment" OR "failure" OR "educational indicator*") AND ("kindergarten" OR "elementary school*" OR "primary school*" OR "high school*" OR "public school") **AND ("random assignment" OR "randomized experiment" OR "experiment*" OR "experimental design" OR "control group" OR "non-experiment" OR "non-experimental" OR "quasi-experiment" OR "quasi-experimental" OR "comparison group" OR "matched comparison group" OR "matched comparison" OR "matched groups" OR "statistical matching" OR "propensity score matching" OR "systematic review" OR "review" OR "meta-analysis" OR "research synthesis" OR "research review")**
  - Number of Articles: 4
  - Source Type: Scholarly Journals

  Search Note: Adding the search terms in bold made the search strategy too long and ineffective. Therefore, we only used **"AND (experiment*)"**.

One reference in JSTOR passed initial screening:

1. Glazerman, S., Mayer, D., & Decker, P. (2006). Alternative routes to teaching: The impacts of Teach for America on student achievement and other outcomes. *Journal of Policy Analysis and Management*, *25*(1), 75–96.

Database: Academic Search Premier

- *Search 1:* (TI ["Teach for America"] OR AB ["Teach for America" OR "TFA Corps"])
  - Number of Articles: 432
  - Source Type: Magazines (283), Academic Journals (42), Newspapers (86), Reviews (17), Trade Publications (3)
- *Search 2:* (TI ["Teach for America"] OR AB ["Teach for America" OR "TFA Corps"]) **AND ("academic achievement" OR "success*" OR "grade level" OR "grading" OR "academic ability" OR "attainment" OR "failure" OR "educational indicator*")**
  - Number of Articles: 138
  - Source Type: Magazines (85), Academic Journals (25), Newspapers (19), Reviews (9)
- *Search 3:* (TI ["Teach for America"] OR AB ["Teach for America" OR "TFA Corps"]) AND ("academic achievement" OR "success*" OR "grade level" OR "grading" OR "academic ability"

OR "attainment" OR "failure" OR "educational indicator*") **AND ("kindergarten" OR "elementary school*" OR "primary school*" OR "high school*" OR "public school")**

  - Number of Articles: 98
  - Source Type: Magazines (55), Academic Journals (20), Newspapers (14), Reviews (9)

- *Search 4:* (TI ["Teach for America"] OR AB ["Teach for America" OR "TFA Corps"]) AND ("academic achievement" OR "success*" OR "grade level" OR "grading" OR "academic ability" OR "attainment" OR "failure" OR "educational indicator*") AND ("kindergarten" OR "elementary school*" OR "primary school*" OR "high school*" OR "public school") **AND ("random assignment" OR "randomized experiment" OR "experiment*" OR "experimental design" OR "control group" OR "non-experiment" OR "non-experimental" OR "quasi-experiment" OR "quasi-experimental" OR "comparison group" OR "matched comparison group" OR "matched comparison" OR "matched groups" OR "statistical matching" OR "propensity score matching" OR "systematic review" OR "review" OR "meta-analysis" OR "research synthesis" OR "research review")**
  - Number of Articles: 92
  - Source Type: Magazines (52), Academic Journals (19), Newspapers (13), Reviews (8)

One reference in Academic Search Premier passed initial screening

1. Antecol, H., Eren, O., & Ozbeklik, S. (2013). The effect of Teach for America on the distribution of student achievement in primary school: Evidence from a randomized experiment. *Economics of Education Review*, *37*, 113–125.

Database: Education Full Text

- *Search 1:* (TI ["Teach for America"] OR AB ["Teach for America" OR "TFA Corps"])
  - Number of Articles: 408
  - Source Type: Magazines (253), Academic Journals (105), Book Reviews (23), Books (15), Newspapers (4), Conference Papers (4), Biographies (1)
- *Search 2:* (TI ["Teach for America"] OR AB ["Teach for America" OR "TFA Corps"]) **AND ("academic achievement" OR "success*" OR "grade level" OR "grading" OR "academic ability" OR "attainment" OR "failure" OR "educational indicator*")**
  - Number of Articles: 172
  - Source Type: Magazines (89), Academic Journals (65), Book Reviews (12), Conference Papers (3), Newspapers (2), Books (2)
- *Search 3:* (TI ["Teach for America"] OR AB ["Teach for America" OR "TFA Corps"]) AND ("academic achievement" OR "success*" OR "grade level" OR "grading" OR "academic ability" OR "attainment" OR "failure" OR "educational indicator*") **AND ("kindergarten" OR "elementary school*" OR "primary school*" OR "high school*" OR "public school")**
  - Number of Articles: 139
  - Source Type: Magazines (70), Academic Journals (50), Book Reviews (12), Conference Papers (2), Newspapers (1), Books (1)
- *Search 4:* (TI ["Teach for America"] OR AB ["Teach for America" OR "TFA Corps"]) AND ("academic achievement" OR "success*" OR "grade level" OR "grading" OR "academic ability" OR "attainment" OR "failure" OR "educational indicator*") AND ("kindergarten" OR "elementary school*" OR "primary school*" OR "high school*" OR "public school") **AND ("random assignment" OR "randomized experiment" OR "experiment*" OR "experimental design" OR "control group" OR "non-experiment" OR "non-experimental" OR "quasi-experiment" OR "quasi-experimental" OR "comparison**

group" OR "matched comparison group" OR "matched comparison" OR "matched groups" OR "statistical matching" OR "propensity score matching" OR "systematic review" OR "review" OR "meta-analysis" OR "research synthesis" OR "research review")

- Number of Articles: 128
- Source Type: Magazines (63), Academic Journals (50), Book Reviews (11), Conference Papers (2), Newspapers (1), Books (1)

Four references in Education Full Text passed the initial screening

1. Antecol, H., Eren, O., & Ozbeklik, S. (2013). The effect of Teach for America on the distribution of student achievement in primary school: Evidence from a randomized experiment. *Economics of Education Review, 37*, 113–125.
2. Darling-Hammond, L., Holtzman, D. J., & Gatlin, S. J. (2005). Does teacher preparation matter? Evidence about teacher certification, Teach for America, and teacher effectiveness. *Education Policy Analysis Archives, 13*(42), 1–47.
3. Glazerman, S., Mayer, D., & Decker, P. (2006). Alternative routes to teaching: The impacts of Teach for America on student achievement and other outcomes. *Journal of Policy Analysis and Management*, *25*(1), 75–96.
4. Xu, Z., Hannaway, J., & Taylor, C. (2011). Making a difference? The effects of Teach for America in high school. *Journal of Policy Analysis and Management, 30*(3), 447–469.

Appendix A.2: Results from grey literature search

Table A.2.1. *Results from Searches of General and Targeted Websites*

Searching						Screening
	Website	Term(s)	Citations	Eligible	Duplicate	Result
1	Abt Associates	Teach for America, TFA, or Corps	0	0	0	Upcoming project: "Study on Promising Teacher Prep Programs"
2	Alliance for Excellent Education	TFA	3	0	0	All reports reference TFA as a source but not as a study
AERA Online Journals:						
3	AERA Open	TFA	1	0	0	TFA referenced as an example, not a study
4	AERJ	TFA	4	0	0	No studies used a comparison group
5	EEPA	TFA	6	0	0	TFA referenced as a source but not a study
6	ER	TFA	0	0	0	–
7	JEBS	TFA	3	0	0	Reviews of TFA; potential source for other studies
8	RER	TFA	1	0	0	Reviews of TFA; potential source for other studies
9	RRE	TFA	1	0	0	Reviews of TFA; potential source for other studies
10	American Enterprise Institute	TFA	27	0	0	Opinion articles about TFA
11	American Institutes for Research	TFA	5	0	0	Mostly news reports
12	Best Evidence Encyclopedia	TFA	0	0	0	–
13	Brookings Institute	TFA	10	0	0	Blogs, memos, etc., but no studies
14	Carnegie Corporation of New York	Teach for America	4	0	0	Reports reference TFA but were not studies
15	Center for Research and Reform in Education	–	0	0	0	Website platform not conducive to a systematic search
16	Congressional Research Service	–	0	0	0	Website platform not conducive to a systematic search

Table A.2.1 (continued)

	**Searching**			**Screening**		
	Website	Term(s)	Citations	Eligible	Duplicate	Result
17	Government Accountability Office	–	0	0	0	Website platform not conducive to a systematic search
18	Grants and contracts awarded by IES	TFA	4	0	0	None were publications
19	Heritage Foundation	–	0	0	0	Website platform not conducive to a systematic search
20	Hoover Institute	TFA	7	0	0	Mostly news reports and articles describing TFA research
21	Mathematica Policy Research	TFA	101	4	3	Identified a literature review
22	MDRC	TFA	1	0	0	Conference report
23	National Association of State Boards of Education	TFA	5	0	0	Narrative reviews and report but no studies
24	National Governors' Association	TFA	1	0	0	News reports
25	Policy Archive	–	0	0	0	Website platform not conducive to a systematic search
26	Policy Study Associates	TFA	10	0	0	Studies were not impact evaluations with comparison group
27	RAND	TFA	4	0	0	Studies did not use a comparison group
28	SRI	TFA	4	0	0	Reports did not use a comparison group
29	Thomas B. Fordham Institute	–	0	0	0	Website platform not conducive to a systematic search
30	Urban Institute	TFA	7	0	0	Studies did not use a comparison group
31	After-School Alliance	TFA	15	0	0	News reports, not studies
32	The Campbell Collaboration	TFA	3	0	0	No new research beyond the current systematic review

Table A.2.1 (continued)

	Searching		Screening			
	Website	Term(s)	Citations	Eligible	Duplicate	Result
33	Carnegie Corporation for the Advancement of Teaching	–	0	0	0	Website platform not conducive to a systematic search
34	Center for Social Organization of Schools—BERC	TFA	0	0	0	–
35	Chapin Hall Center for Children	TFA	0	0	0	–
36	CINAHL	TFA	2	0	0	Reports do not use a comparison group
37	Cochrane Central Register of Controlled Trial	TFA	0	0	0	–
		Teach for America	0	0	0	–
38	Cochrane Database of Systematic Reviews	TFA	0	0	0	–
		Teach for America	0	0	0	–
39	Database of Abstracts of Reviews of Effects	TFA	0	0	0	–
40	Florida Center for Reading Research	–	0	0	0	Website platform not conducive to a systematic search
		Teach for America	0	0	0	–
41	Harvard Family Research Project	TFA	0	0	0	Opinion articles
42	Institute for Higher Education Policy	TFA	0	0	0	–
43	Institute for Public Policy and Social Research	TFA	0	0	0	–
44	Natl. Association of State Directors of Career Tech. Ed.	–	0	0	0	Website platform not conducive to a systematic search
44	NBER Working Papers	TFA	6	0	0	Valued-added or RDD studies
	**Total**		**235**	**4**	**3**	

One website reference passed initial screening

1. Clark, M. A., Isenberg, E., Liu, A. Y., Makowsky, L., & Zukiewicz, M. (2016, March 4). *Impacts of the Teach for America Investing in Innovation scale-up*. Retrieved from http://www.mathematica-mpr.com/our-publications-and-findings/publications/impacts-of-the-teach-for-america-investing-in-innovation-scaleup

Table A.2.2. *Results from Searches of Grey Literature Databases (Including Conference Proceedings and Google)*

	**Searching**			**Screening**		
	Website	Term(s)	Citations	Relevant	Duplicate	Result
1	PolicyFile	Teach for America	12	4	4	Four studies retrieved from previous database searches
		TFA	0	0	0	
2	PsycEXTRA	–	–	–	–	Website not conducive to a systematic search
3	OpenGrey.eu	TFA	1	0	0	
4	EditLib	Teach for America	14	0	0	
		TFA	10	0	0	
5	Index of Conference Proceedings	–	–	–	–	Website not conducive to a systematic search
6	Google	(“Teach for America” or “TFA”) AND (“Math” or “Science” or “Language Arts”) AND (“impact” or “effect”)	359	23	14	Identified six studies that were not identified in previous searches.
	**Total**		**396**	**27**	**18**	

Eight references from grey literature database searches passed the screening and were not duplicates from previous database searches

1. Boyd, D., Grossman, P., Lankford, H., Loeb, S., & Wyckoff, J. (2006). *How changes in entry requirements alter the teacher workforce and affect student achievement.* Columbia, MO: American Education Finance Association. Retrieved from https://cepa.stanford.edu/sites/default/files/Reducing Entry Requirements EPF 2006.pdf
2. Noell, G. H., & Gansle, K. A. (2009). *Teach For America teachers' contribution to student achievement in Louisiana in grades 4–9: 2004–2005 to 2006–2007.* Baton Rouge, LA: Louisiana Board of Regents.
3. Henry, G. T., Thompson, C. L., Bastian, K. C., Fortner, K. C., Kershaw, D. C., Purrell, K. M., & Zulli, R. A. (2010, June). *Portal report: Teacher preparation and student test scores in North Carolina.* Retrieved from http://www.worldcat.org/title/portal-report-teacher-preparation-and-student-test-scores-in-north-carolina/oclc/789248583
4. Ware, A., LaTurner, J. R., Parsons, J., Okulicz-Kozaryn, A., Garland, M., & Klopfenstein, K. (2011). *Teacher preparation programs and Teach for America research study* (Rep.). Retrieved from https://www.researchgate.net/publication/236333015_Evaluation_of_Teach_For_America_in_Texas_Schools
5. Turner, H. M., Goodman, D., Adachi, E., Brite, J., & Decker, L. E. (2012, December). *Evaluation of Teach for America in Texas schools.* Retrieved from http://http://edvanceresearch.com/wp-content/uploads/2015/06/Evaluation-of-Teach-For-America-in-Texas-Schools.pdf
6. Ready, D. D. (2014). *Teach for America teachers in Duval County public schools: An analysis of retention and performance.* Retrieved from https://www.tc.columbia.edu/faculty/ddr2111/faculty-profile/files/FINAL_TFA_DUVAL.pdf
7. Boyd, D., Grossman, P., Hammerness, K., Lankford, H., Loed, S., Ronfeldt, M., & Wyckoff, J. (2012). Recruiting effective math teachers: Evidence from New York City. *American Educational Research Journal, 49*(6), 1008–1047.
8. Clark, M. A., Isenberg, E., Liu, A. Y., Makowsky, L., & Zukiewicz, M. (2016, March 4). *Impacts of the Teach for America Investing in Innovation scale-up.* Retrieved from http://www.mathematica-mpr.com/our-publications-and-findings/publications/impacts-of-the-teach-for-america-investing-in-innovation-scaleup

Appendix A.3: Study referrals

Table A.3.1. *Study Referrals from a Random Sample of Researchers*

	Researcher	Referral	Duplicate Study
1	Dan Goldhaber	None.	–
2	Gene Glass	Laczko-Kerr, I., & Berliner, D. C. (2002, September 6). The effectiveness of "Teach for America" and other under-certified teachers on student academic achievement: A case of harmful public policy. *Education Policy Analysis Archives*, *10*(37). Retrieved from http://epaa.asu.edu/epaa/v10n37	Yes
3	Lawrence Baines	Anecdote on TFA corps members.	
4	Linda Darling-Hammond	Assistant responded that they did not have time to provide data.	
5	Melissa Clark	Clark, M. A., Chiang, H. S., Silva, T., McConnell, S., Sonnenfeld, K., Erbe, A., & Puma, M. (2013). *The effectiveness of secondary math teachers from Teach for America and the teaching fellows programs* (NCEE 2013-4015). Washington, DC: National Center for Education Evaluation and Regional Assistance, Institute of Education Sciences, U.S. Department of Education.	Yes
		Decker, P., Mayer, D., & Glazerman, S. (2004). *The effects of Teach for America on students: Findings from a national evaluation*. Princeton, NJ: Mathematica Policy Research, Inc.	Yes
		Clark, M. A., Isenberg, E., Liu, A. Y., Makowsky, L., & Zukiewicz, M. (2016, March 4). *Impacts of the Teach for America Investing in Innovation scale-up*. Retrieved from http://www.mathematica-mpr.com/our-publications-and-findings/publications/impacts-of-the-teach-for-america-investing-in-innovation-scaleup	Yes

Note: These 5 researchers were randomly selected from a list of 25 researchers identified via the reference list from cursory searches used during protocol development.

Table A.3.2. *Random Sampling of Researchers*

**Author Sampling Frame**		**Author Sample**		
**Author Last Name**	**Random Number**	**Author Last Name**	**Random Number (Sorted)**	**Selected**
Xu	0.435	Clark	0.056	X
Fetler	0.284	Goldhaber	0.112	X
Clofelter	0.428	Darling-Hammond	0.180	X
Constantine	0.618	Baines	0.240	X
Noell	0.776	Glass	0.251	X
Hess	0.696	Decker	0.276	
Goldhaber	0.112	Fetler	0.284	
Veltri	0.388	Laczko-Kerr	0.290	
Helig	0.464	Wilson	0.346	
Jones	0.899	Kane	0.387	
Darling-Hammond	0.180	Veltri	0.388	
Boyd	0.513	Baines	0.393	
Ware	0.595	Clofelter	0.428	
Parsons	0.833	Xu	0.435	
Decker	0.276	Helig	0.464	
Antecol	0.951	Boyd	0.513	
Wilson	0.346	Ware	0.595	
Baines	0.393	Constantine	0.618	
Clark	0.056	Hess	0.696	
Glass	0.251	Raymond	0.697	
Laczko-Kerr	0.290	Noell	0.776	
Kane	0.387	Parsons	0.833	
Raymond	0.697	Goe	0.890	
Baines	0.240	Jones	0.899	
Goe	0.890	Antecol	0.951	

Note: The author sample consisted of the five authors with the lowest random numbers.

From: Michèle Muñoz-Miller [mailto:michele@analytica-inc.com]
Sent: Tuesday, May 12, 2015 9:34 PM
To: Melissa Clark <MClark@mathematica-mpr.com>
Subject: Request for TFA citations

Hello Dr. Clark,

A research team at ANALYTICA is conducting a Campbell Collaboration systematic review on the impact of Teach For America on student academic achievement.

We are reaching out to researchers for references to randomized controlled trials or quasi-experiments with a comparison group that may be relevant to the review.

We would appreciate referrals to relevant citations from you.

Thanks in advance for your help.

Michèle

ANALYTICA, Inc.
35 Goldfinch Circle | Phoenixville | PA | 19460
T: 215.808.8880 | F: 610.933.1005 |

Appendix A.4: Results from hand searches of journals

Table A.4. *Results of Hand Searches of Selected Journals for 2014–2015*

Searching				Screening		
**No.**	**Journal**	**Terms**	**Articles**	**Relevant**	**Duplicate**	**Volume and Issues Reviewed**
1	*American Educational Research*	Teach for America or TFA	5	0	0	Reviewed 2014 Volume 51 (6 issues)<br>Reviewed 2015 Volume 52 (6 issues)
2	*American Economic Association*	–	0	0	0	Website platform not conducive to a systematic hand search of journals
3	*Journal of Policy Analysis and Management*	Teach for America or TFA	1	1	0	Reviewed 2014 Volume 33 (4 issues)<br>Reviewed 2015 Volume 34 (4 issues)<br>Reviewed 2016 Volume 35 (1 issue)
4	*Economics of Education Review*	Teach for America or TFA	0	0	0	Reviewed 2014-2016 Volumes 41–50
5	*Education Policy Analysis Archives*	Teach for America or TFA	12	0	0	Website platform not conducive to a systematic hand search of journals
6	*Journal of Human Resources*	Teach for America or TFA	0	0	0	Reviewed 2014 Volume 49 (4 journals)<br>Reviewed 2015 Volume 50 (4 journals)<br>Reviewed 2016 Volume 51 (1 journal)
	**Total**		**17**	**1**	**0**	

One reference from hand searches passed initial screening

1. Dee, T. S., & Wyckoff, J. (2015). Incentives, selection, and teacher performance: Evidence from IMPACT. *Journal of Policy Analysis and Management*, *34*(2), 267–297.

Appendix B: Results of author queries

Table B.1. *Status of Author Queries for Studies Eligible for Inclusion in the Systematic Review*

Study Reference	Primary Contacts	Status	Explanation
Antecol, H., Eren, O., & Ozbeklik, S. (2013). The effect of Teach for America on the distribution of student achievement in primary school: Evidence from a random experiment. *Economics of Education Review*, *37*, 113–125.	Heather Antecol	**No response**	Sent author query twice.
Boyd, D., Grossman, P., Lankford, H., Loeb, S., & Wyckoff, J. (2006). *How changes in entry requirements alter the teacher workforce and affect student achievement.* Columbia, MO: American Education Finance Association. Retrieved from https://cepa.stanford.edu/sites/default/files/Reducing Entry Requirements EPF 2006.pdf	Don Boyd	**Responded**	Boyd said data are not available due to confidentiality agreements with Texas Education Agency (TEA). Data have been discarded.
Boyd, D., Grossman, P., Hammerness, K., Lankford, H., Loed, S., Ronfeldt, M., & Wyckoff, J. (2012). Recruiting effective math teachers: Evidence from New York City. *American Educational Research Journal*, *49*(6), 1008–1047.	Don Boyd	**Responded**	Boyd said data are not available due to confidentiality agreements with TEA. Data have been discarded.
Carroll, C. A. (2013). *The influence of Teach for America on Algebra I student achievement*. Unpublished doctoral dissertation, University of North Carolina, Charlotte.	Curtis Carroll	**No response**	Sent author query twice.
Clark, M., Chiang, H., Silva, T., McConnell, S., Sonnenfeld, K., & Erbe, A. (2013, September). *The effectiveness of secondary math teachers from Teach for America and the teaching fellow programs*. NCEE 2013-4016. Washington, DC: U.S. Department of Education.	Melissa Clark	**Responded**	Clark sent data.
Clark, M. A., Isenberg, E., Liu, A. Y., Makowsky, L., & Zukiewicz, M. (2016, March 4). *Impacts of the Teach for America Investing in Innovation scale-up*. Retrieved from http://www.mathematica-mpr.com/our-publications-and-findings/publications/impacts-of-the-teach-for-america-investing-in-innovation-scaleup	Melissa Clark	**Responded**	Clark sent data.

Table B.1 (continued)

**Study Reference**	**Primary Contacts**	**Status**	**Explanation**
Darling-Hammond, L., Holtzman, D. J., Gatlin, S. J., & Heilig, J. V. (2005). Does teacher preparation matter? Evidence about teacher certification, Teach for America, and teacher effectiveness. *Education Policy Analysis Archives*, *13*(42). Retrieved from http://epaa.asu.edu/epaa/v13n42/	Linda Darling-Hammond	**Responded**	Student of Dr. Darling-Hammond said data are not available.
Decker, P., Mayer, D., & Glazerman, S. (2004). *The effects of Teach for America on students: Findings from a national evaluation*. Princeton, NJ: Mathematica Policy Research, Inc.	Steve Glazerman	**Responded**	Glazerman sent public use file and responded to questions.
Noell, G. H., & Gansle, K. A. (2009). *Teach For America teachers' contribution to student achievement in Louisiana in grades 4–9: 2004–2005 to 2006–2007*. Baton Rouge, LA: Louisiana Board of Regents.	George Noell	**Responded**	Sent data as an Excel spreadsheet
Ware, A., LaTurner, R., Parsons, J., Okulicz-Kozaryn, A., Garland, M., & Klopfenstein, K. (2011). *Teacher preparation programs and Teach for America research Study*. Dallas, TX: The University of Texas at Dallas Education Research Center.	Anne Ware	**Responded**	Ware requested a copy of study. A copy was sent to her, and she reviewed it, but she did not send any data in response.

Figure B.1. *Example of author query email.*

Dear [INSERT AUTHOR NAME],

I hope all is well with you.

ANALYTICA was awarded a Campbell Collaboration grant to conduct the first-ever high-quality systematic review on the impact of Teach for America on student academic outcomes. Your study, cited below, has been selected for inclusion in this review:

[Insert Study Citation]

We plan to present the information from your study as accurately as possible, especially since this review will likely garner high visibility both domestically and internationally.

The attached document requests data needed to accurately evaluate the quality of the evidence presented in your study. **We would like to receive this information by [INSERT DATE].**

However, we understand that we are in the holiday season and you may need more time. If so, please contact me about a feasible date. **If I do not hear from you by [INSERT DATE], we will proceed based on the information published in the study.**

Thanks,

Herb

Herbert M. Turner, III | President & Principal Scientist | ANALYTICA, Inc.
Adjunct Associate Professor | PENN GSE
35 Goldfinch Circle | Phoenixville | PA | 19460
T: 215.808.8880 | F: 610.933.1005 |

Appendix C: Coding results for review-eligible studies (*n* = 24)

Table C.1. *Coding Results for Studies that Were Eligible for Review and Coding*

No.	Studies Eligible for Coding	Coding Result				
		Design	Passed Stage1	Passed Stage 2	Author Query	In Meta-Analysis
1	Antecol, H., Eren, O., & Ozbeklik, S. (2013)	RCT	Yes	No	Yes	No
2	Bastian, K. C. (2014)	TVA	Yes	No	Yes	No
3	Boyd, D., Grossman, P., Lankford, H., Loeb, S., & Wyckoff, J. (2006)	TVA	Yes	No	Yes	No
4	Boyd, D., Grossman, P., Hammerness, K., Lankford, H., Loed, S., Ronfeldt, M., & Wyckoff, J. (2012)	TVA	Yes	No	Yes	No
5	Carroll, C. A. (2013)	QED	Yes	No	Yes	No
6	Clark, M. A., Chiang, H., Silva, T., McConnell, S., Sonnenfeld, K., & Erbe, A. (2013)	RCT	Yes	Yes	Yes	Yes
7	Clark, M. A., Isenberg, E., Liu, A. Y., Makowsky, L., & Zukiewicz, M. (2016)	RCT	Yes	Yes	Yes	Yes
8	Darling-Hammond, L., Holtzman, D. J., & Gatlin, S. J. (2005)	QED	Yes	No	Yes	No
9	Dee, T. S., & Wyckoff, J. (2015)	RDD	No	-	-	No
10	Evans, B. R. (2009)	COR	N0	-	-	No
11	Evans, B. R. (2010)	COR	No	-	-	No
12	Decker, P., Mayer, D., & Glazerman, S. (2004); Glazerman, S., Mayer, D., & Decker, P. (2006)	RCT	Yes	Yes	Yes	Yes
13	Henry, G. T., Thompson, C. L., Bastian, K. C., Fortner, K. C., Kershaw, D. C., Purrell, K. M., & Zulli, R. A. (2010)	TVA	Yes	No	Yes	No
14	Laczko-Kerr, I. I. (2002)	QED	Yes	No	Yes	No
15	Noell, G., & Gansle, K. A. (2009)	TVA	Yes	No	Yes	No
16	Pearson, J. L. (2014)	TVA	Yes	No	Yes	No
17	Penner, E. K. (2014)	QED	Yes	No	No	No
18	Prescott, S. H. (2010)	QED	No	-	-	No
19	Raymond, M., Fletcher, S., & Luque, J. (2001); Raymond, M., & Fletcher, S. (2002)	QED	Yes	No	No	No
20	Ready (2014)	QED	Yes	No	No	No
21	Tennessee State Board and Higher Education Commission (2010)	QED	Yes	No	No	No
22	Turner, H. M., Goodman, D., Adachi, E., Brite, J., & Decker, L. E. (2012)	QED	Yes	Yes	No	Yes
23	Ware, A., LaTurner, R., Parsons, J., Okulicz-Kozaryn, A., Garland, M., & Klopfenstein, K. (2011)	QED	Yes	Yes	Yes	No
24	Xu, Z., Hannaway, J., & Taylor, C. (2009); Xu, Z., Hannaway, J., & Taylor, C. (2011)	QED	Yes	No	Yes	No
**Total**		**24**	**21**	**4**	**15**	**4**

Note: TVA = "Teacher Value Added," RDD = "Regression Discontinuity Design," and LR = "Literature Review." Study 1 used the same data as study 10, for different reasons.

Table C.2. *Request for Study Information from Authors of TFA Studies that Passed Stage 1 or Stage 2 Screening*

No.	Study Reference	Primary Contact	Response	Explanation
1	Antecol, H., Eren, O. & Ozbeklik, S. (2013). The effect of Teach for America on the distribution of student achievement in primary school: Evidence from a random experiment. *Economics of Education Review*, *37*, 113–125.	Heather Antecol	**No**	Sent author query twice.
3	Boyd, D., Grossman, P., Lankford, H., Loeb, S., & Wyckoff, J. (2006). *How changes in entry requirements alter the teacher workforce and affect student achievement. Columbia, MO: American Education Finance Association*. Retrieved from https://cepa.stanford.edu/sites/default/files/Reducing Entry Requirements EPF 2006.pdf	Dan Boyd	**Yes**	Data were destroyed.
4	Boyd, D., Grossman, P., Hammerness, K., Lankford, H., Loed, S., Ronfeldt, M., & Wyckoff, J. (2012). Recruiting effective math teachers: Evidence from New York City. American Educational Research Journal, 49(6), 1008–1047.	Dan Boyd	**Yes**	Data were destroyed.
5	Carroll, C. A. (2013). *The influence of Teach for America on Algebra I student achievement*. Unpublished doctoral dissertation, University of North Carolina, Charlotte.	Curtis Carroll	**No**	Sent author query twice.
6	Clark, M., Chiang, H., Silva, T., McConnell, S., Sonnenfeld, K., & Erbe, A. (2013, September). *The effectiveness of secondary math teachers from Teach for America and the Teaching Fellow Programs*. NCEE 2013-4016. Washington, DC: U.S. Department of Education.	Melissa Clark	**Yes**	Clark sent data.
7	Clark, M. A., Isenberg, E., Liu, A. Y., Makowsky, L., & Zukiewicz, M. (2016, March 4). *Impacts of the Teach For America Investing in Innovation Scale-Up*. Retrieved from http://mathematica-mpr.com	Melissa Clark	**Yes**	Clark sent data.
8	Darling-Hammond, L., Holtzman, D. J., Gatlin, S. J., & Heilig, J. V. (2005). Does teacher preparation matter? Evidence about teacher certification, Teach for America, and teacher effectiveness. *Education Policy Analysis Archives*, *13*(42). Retrieved from http://epaa.asu.edu/epaa/v13n42/	Linda Darling-Hammond	**No**	The Office of Linda-Darling Hammond sent a message saying that she is out of town.

Table C.2 (continued)

No.	Study Reference	Primary Contact	Response	Explanation
12	Decker, P., Mayer, D., & Glazerman, S. (2004). *The Effects of Teach For America on Students: Findings from a National Evaluation.* Princeton, NJ: Mathematica Policy Research, Inc.	Steve Glazerman	Yes	-
13	Henry, G. T., Thompson, C. L., Bastian, K. C., Fortner, K. C., Kershaw, D. C., Purrell, K. M., & Zulli, R. A. (2010, June). *Portal report: Teacher preparation and student test scores in North Carolina*. Retrieved from http://www.worldcat.org/title/portal-report-teacher-preparation-and-student-test-scores-in-north-carolina/oclc/789248583	Gary Henry	**No**	–
14	Laczko-Kerr, I. I. (2002). *Teacher certification does matter: The effects of certification status on student achievement.* Unpublished doctoral dissertation, Arizona State University, Tempe.	Ildiko Laczko-Kerr	**Yes**	Confirmed that analysis was done using classroom-level data.
15	Noell, G., & Gansle K. (2009). *Teach for America teacher's contribution to student achievement in Louisiana in grades 4–9: 2004–2005 to 2006–2007.* Baton Rouge, LA: Louisiana Board of Regents.	George Noell	**Yes**	Sent data as an Excel spreadsheet.
16	Pearson, J. L. (2014). *Effective instructional methods utilized in successful and high performing secondary schools in the Southern Region of Mississippi.* Unpublished doctoral dissertation, The University of Southern Mississippi, Hattiesburg.	Jane Pearson	**No**	–
23	Ware, A., LaTurner, R., Parsons, J., Okulicz-Kozaryn, A., Garland, M., & Klopfenstein, K. (2011). *Teacher Preparation Programs and Teach for America Research Study*. Dallas, TX: The University of Texas at Dallas Education Research Center.	Anne Ware	**Yes**	Ware requested copy of the report. Copy was sent. Ware reviewed but did not submit data.
24	Xu, Z., Hannaway, J., & Taylor, C. (2011). Making a difference? The effects of Teach for America in high school. *Journal of Policy Analysis and Management, 30*(3), 447–469.	Zeyu Xu	**No**	Author query sent twice.

Appendix D: Excluded studies and reasons

1. Antecol, H., Eren, O., & Ozbeklik, S. (2013). The effect of Teach for America on the distribution of student achievement in primary school: Evidence from a randomized experiment. *Economics of Education Review 37*, 113–125.

   Reason: Failed Stage 2 review. Used same data as Decker, Mayer, and Glazerman (2004) but estimated effects of TFA at different quartiles of the outcome distribution. For estimating ATE, this study was excluded from the meta-analysis to avoid double-counting ATE.

2. Bastian, K. C. (2014). Selecting and preparing teachers and school leaders to improve educational outcomes. *Dissertation Abstracts International: The Humanities and Social Sciences.*

   Reason: Failed Stage 2 review. Predictive study that uses value-added design. Author responded to query but could not provide the data needed to evaluate study as a QED, given the time frame.

3. Boyd, D., Grossman, P., Lankford, H., Loeb, S., & Wyckoff, J. (2006). *How changes in entry requirements alter the teacher workforce and affect student achievement.* Columbia, MO: American Education Finance Association. Retrieved from https://cepa.stanford.edu/sites/default/files/Reducing Entry Requirements EPF 2006.pdf

   Reason: Failed Stage 2 review. Used value-added design. Author responded to query but could not provide the data needed to evaluate study as a QED, given the time frame.

4. Boyd, D., Grossman, P., Hammerness, K., Lankford, H., Loed, S., Ronfeldt, M., & Wyckoff, J. (2012). Recruiting effective math teachers: Evidence from New York City. *American Educational Research Journal, 49*(6), 1008–1047.

   Reason: Failed Stage 2 review. Used value-added design. Author responded to query but could not provide the data needed to evaluate study as a QED, given the time frame. Insufficient data to evaluate study as a QED.

5. Carroll, C. A. (2013). *The influence of Teach for America on Algebra I student achievement.* Unpublished doctoral dissertation, University of North Carolina, Charlotte.

   Reason: Failed Stage 2 review. Insufficient data to establish baseline equivalence between groups in analysis sample.

6. Darling-Hammond, L., Holtzman, D. J., & Gatlin, S. J. (2005). Does teacher preparation matter? Evidence about teacher certification, Teach for America, and teacher effectiveness. *Education Policy Analysis Archives, 13*(42), 1–47.

   Reason: Failed Stage 2 review. Insufficient data to establish baseline equivalence between groups in the analysis sample.

7. Dee, T. S., & Wyckoff, J. (2015). Incentives, selection, and teacher performance: Evidence from IMPACT. *Journal of Policy Analysis and Management, 34*(2), 267–297.

   Reason: Failed Stage 1 review. Study did not examine the effects of TFA corps members or alumni.

8. Evans, B. R. (2009). First year middle and high school teachers' mathematical content proficiency and attitudes: Alternative certification in the Teach for America (TFA) program. *Journal of the National Association for Alternative Certification, 4*(1), 3–17.

   Reason: Failed Stage 1 review. Correlational study design that is not an eligible design.

9. Evans, B. R. (2010). Determining quality teachers: Mathematical content knowledge, perceptions of teaching self-efficacy, and attitudes toward mathematics among a Teach for America cohort. *Journal of the National Association for Alternative Certification, 5*(2), 23–35.

   Reason: Failed Stage 1 review. Correlational study design that is not an eligible design.

10. Henry, G. T., Thompson, C. L., Bastian, K. C., Fortner, K. C., Kershaw, D. C., Purrell, K. M., & Zulli, R. A. (2010, June). *Portal report: Teacher preparation and student test scores in North Carolina.* Retrieved from http://www.worldcat.org/title/portal-report-teacher-preparation-and-student-test-scores-in-north-carolina/oclc/789248583

    Reason: Failed Stage 2 review. Used teacher value-added design. Author did not respond to query. Data were not available to evaluate study as a QED. Insufficient data to evaluate study as a QED.

11. Laczko-Kerr, I. I. (2002). *Teacher certification does matter: The effects of certification status on student achievement.* Unpublished doctoral dissertation, Arizona State University, Tempe.

    Reason: Failed Stage 2 review. Study makes causal inferences for student outcomes using classroom-level data. Student data were not available to establish baseline equivalence between groups, at the student level, in the analysis sample.

12. Noell, G. H., & Gansle, K. A. (2009). *Teach For America teachers' contribution to student achievement in Louisiana in grades 4–9: 2004–2005 to 2006–2007.* Baton Rouge, LA: Louisiana Board of Regents.

    Reason: Failed Stage 2 review. Pretest data in the analysis sample were imputed. Therefore, we could not establish baseline equivalence between groups in analysis sample.

13. Pearson, J. L. (2014). *Effective instructional methods utilized in successful and high performing secondary schools in the Southern Region of Mississippi.* Unpublished doctoral dissertation, The University of Southern Mississippi, Hattiesburg.

    Reason: Failed Stage 2 review. Used value-added design. Author responded to query but could not provide data needed to evaluate study as a QED, given the time frame.

14. Penner, E. K. (2014). *Teaching for all? Variation in the effects of Teach for America.* Unpublished doctoral dissertation, University of California, Irvine.

    Reason: Failed Stage 2 review. Could not establish equivalence between groups because researchers imputed data in the analysis sample. For this review, baseline equivalence can be established using only non-imputed data.

15. Prescott, S. H. (2010). *The effects of affirmative quality feedback on low socio-economic students' zone of proximal development reading gains (ZPDRL): A causal-comparative study.* Unpublished doctoral dissertation, The University of Mississippi, Oxford.

    Reason: Failed Stage 1 review. TFA was delivered for only one semester and not the full school year (as required in the protocol).

16. Raymond, M., & Fletcher, S. (2002, Spring). The Teach for America Evaluation: Herewith, the first evidence on its recruits' actual performance in the classroom. *Education Next,* 62–68.

    Raymond, M., Fletcher, S., & Luque, J. (2001). *Teach for America: An evaluation of teacher differences and student outcomes in Houston, Texas.* Stanford, CA: The Center for Research on Education Outcomes.

    Reason: Failed Stage 2 review. Insufficient data to establish baseline equivalence between groups in analysis sample.

17. Ready, D. D. (2014). *Teach for America Teachers in Duval County Public Schools: An Analysis of Retention and Performance.* Retrieved from https://www.tc.columbia.edu/faculty/ddr2111/facultyprofile/files/FINAL_TFA_DUVAL.pdf

Reason: Failed Stage 2 review. Insufficient data to establish baseline equivalence between groups in analysis sample.

18. Tennessee Higher Education Commission. (2010). *Report Card on the Effectiveness of Teacher Training Programs.* Nashville, TN: State Board of Education and Tennessee Higher Education Commission.

Reason: Failed Stage 2 review. Used teacher value-added design. Did not respond to author query. Insufficient data to evaluate study as a QED.

19. Ware, A., LaTurner, J. R., Parsons, J., Okulicz-Kozaryn, A., Garland, M., & Klopfenstein, K. (2011). *Teacher Preparation Programs and Teach for America Research Study* (Rep.). Retrieved from https://www.researchgate.net/publication/236333015_Evaluation_of_Teach_For_America_in_Texas_Schools

Reason: Lacked contrasts comparable to other studies. Study reported contrasts that "Meet Evidence Criteria" *with reservations*, but none of the other included studies reported similar contrasts that pooled either elementary or middle school grade levels. Moreover, none of the other included studies used the Non-TFA novice comparison at the high school level.

20. Xu, Z., Hannaway, J., Taylor, C., & Urban Institute, National Center for Analysis of Longitudinal Data in Education Research. (2009). *Making a difference? The effects of Teach for America in high school. Working paper 17. Revised.* Washington, DC: National Center for Analysis of Longitudinal Data in Education Research.

Xu, Z., Hannaway, J., & Taylor, C. (2011). Making a difference? The effects of Teach for America in high school. *Journal of Policy Analysis and Management*, *30*(3), 447–469.

Reason: Failed Stage 1 review. Insufficient data to establish baseline equivalence between groups in the analysis sample.

Appendix E: Contrasts reported in included studies

Table E.1. *Contrasts Reported for TFA Studies that Passed Stage 1 or Stage 2 Screening*

Study	Contrasts	Grade	Outcome	Met Evidence Criteria
Decker, Mayer, & Glazerman, 2004	First Year TFA vs. Non-TFA	Elementary	Math	CNBE[1]
	First Year TFA vs. Non-TFA	Elementary	Reading	CNBE
	Second Year and Veteran TFA vs. Non-TFA	Elementary	Math	CNBE
	Second Year and Veteran TFA vs. Non-TFA	Elementary	Reading	CNBE
	TFA vs. Non-TFA	Elementary	Math	Yes
	TFA vs. Non-TFA	Elementary	Reading	Yes
	TFA vs. Non-TFA Certified	Elementary	Math	CNBE
	TFA vs. Non-TFA Certified	Elementary	Reading	CNBE
	TFA vs. Non-TFA Uncertified	Elementary	Math	CNBE
	TFA vs. Non-TFA Uncertified	Elementary	Reading	CNBE
	TFA vs. Non-TFA Veteran	Elementary	Math	CNBE
	TFA vs. Non-TFA Veteran	Elementary	Reading	CNBE
Clark et al., 2015	TFA Corps vs. Non-TFA: Grades 3 to 5	Elementary	Math	Yes
	TFA Corps vs. Non-TFA: Grades 3 to 5	Elementary	Reading	Yes
	TFA Corps vs. Non-TFA: Grades PreK to 2	Elementary	Math	Yes
	TFA Corps vs. Non-TFA: Grades PreK to 2	Elementary	Reading	Yes
	TFA Corps vs. Non-TFA: Grades PreK to 5	Elementary	Math	Yes
	TFA Corps vs. Non-TFA: Grades PreK to 5	Elementary	Reading	Yes
	TFA Corps vs. Non-TFA: Grades PreK to K	Elementary	Math	Yes
	TFA Corps vs. Non-TFA: Grades PreK to K	Elementary	Reading	Yes
	TFA Corps vs. Non-TFA, Trad.[2] Certified	Elementary	Math	Yes
	TFA Corps vs. Non-TFA, Trad. Certified	Elementary	Reading	Yes
	TFA Corps Novice vs. Non-TFA: Grades PreK to 5	Elementary	Math	Yes

Table E.1 (continued)

Study	Comparison	Grade	Outcome	Meet Evidence Criteria
Clark et al., 2013	TFA Corps Novice vs. Non-TFA: Grades PreK to 5)	Elementary	Reading	Yes
	First Year TFA vs. Non-TFA, Experienced	Middle and High	Math	Yes
	Second Year TFA vs. Non-TFA, Experienced	Middle and High	Math	Yes
	TFA Corps vs. Non-TFA Experienced	Middle and High	Math	Yes
	TFA Corps vs. Non-TFA Novice Teachers	Middle and High	Math	Yes
	TFA vs. Non-TFA	Middle and High	Math	Yes
	TFA vs. Non-TFA: High School	High	Math	Yes
	TFA vs. Non-TFA: Middle School	Middle	Math	Yes
	TFA vs. Non-TFA Alternative Route	Middle and High	Math	Yes
	TFA vs. Non-TFA Trad. Route	Middle and High	Math	Yes
Turner et al., 2012	TFA Corps vs. Non-TFA, Novice	Middle	Math	Yes, WRS[3]
	TFA Corps vs. Non-TFA, Novice	Middle	Reading	Yes, WRS
	TFA Alumni vs. Non-TFA, Veteran	Middle	Math	Yes, WRS
	TFA Alumni vs. Non-TFA, Veteran	Middle	Reading	Yes, WRS

[1] CNBE = Could Not Be Evaluated (because the information for the comparison was not reported).

[2] Trad. = traditional.

[3] WRS = With Reservations.

Appendix F: Risk of bias

Table F.1. *Assessing Risk of Bias in Primary Studies for TFA Systematic Review*

Criteria	Description
Screening	*To be included in the design quality review, a study must have all the characteristics listed below.*
Focus	The study must focus on the effectiveness of the TFA intervention.
Time	The study must be published or reported between 1995 and the present.
Age	The study must focus on students in grades K–12.
Location	TFA must be implemented in the United States.
Outcome	The study must report at least one student academic outcome in math, English language arts, or science.
Exposure	Students in the TFA group must have at least one school year of exposure to the TFA corps member or alumni before outcome measurement.
Setting	The study of TFA must take place in a U.S. public or charter school.
Design	The TFA and counterfactual groups must be formed with random assignment or quasi-experimental methods.
Design Quality	*To be included in the statistical synthesis, a contrast must satisfy all the criteria listed below.*
Bundled Treatment Group Confound	To qualify as a treatment group, all treatment teachers must be TFA corps members or TFA alumni.
Bundled Intervention Confound	The TFA intervention must *not* be completely aligned with another intervention, such as another alternative route teacher preparation program.
$N = 1$ Confound	Each condition required more than one unit at each level (student, teacher, school, district, state, and so on). If there was only one unit at a level, the unit was controlled for across groups.
Outcome Face Validity	A description of the outcome must provide evidence that the measure is well-defined, is interpretable, and measures what it is purported to measure.
Outcome Reliability	Measures must demonstrate an internal consistency reliability of 0.50, inter-rater reliability of 0.50, or temporal stability reliability of 0.40.
Outcome Alignment	Outcomes must not be over-aligned with the intervention.
Outcome Measure Confound	Outcomes must be measured in the same way for both conditions.
Standardized Outcomes	Standardized outcomes are assumed to satisfy the face validity, reliability, alignment, and measurement confound criteria.
Attrition	RCTs must demonstrate low attrition; otherwise, a baseline equivalence test is required. Attrition is considered high if the combination between overall and differential attrition exceeds the thresholds defined by WWC's liberal attrition standard.
Cluster Attrition	Cluster RCTs must test for high attrition at the cluster level. In addition, they must test for high attrition at the sub-cluster level using the clusters with outcome data.

Table F.1 (continued)

Criteria	Description
***Design Quality* (cont.)**	*To be included in the statistical synthesis, a contrast must satisfy all the criteria listed below.*
Baseline Equivalence	All QEDs and RCTs with high attrition must use a pre-intervention measure of the outcome to show evidence of baseline equivalence on the analysis sample.<br>• If the absolute value of the effect size is less than or equal to 0.05, the groups are considered equivalent.<br>• If the absolute value of the effect size is greater than 0.05 and less than or equal to 0.25, the analysis model must statistically adjust for the pre-intervention measure.<br>• If the absolute value of the effect size is greater than 0.25, the outcome is not eligible for the statistical synthesis.

All studies that satisfied the above criteria and had sufficient outcome date were eligible for inclusion in the statistical synthesis and assigned a study rating as follows:

- RCTs with no confounds, reliable outcomes, and low attrition received a rating of meets design quality standards without reservations.
- RCTs with high attrition and QEDs with no confounds, reliable outcomes, and baseline group equivalence in analysis samples received a rating of meets design quality standards with reservations.

Criteria	Description
Risk of Bias Conduct	*The following describes how this review complied with the* Adaptations on MECIR [Methodological Expectations of Campbell Collaboration Intervention Reviews] Version 2.2 Reporting Standards *on issues related to risk of bias.*
Assessing Risk of Bias/Study Quality	The risk of bias was assessed for all RCTs and QEDs that passed screening, using the design criteria outlined in the *WWC Procedures and Standards Handbook, Version 3.0*. The specific criteria are outlined above, under Design Quality.
Assessing Risk of Bias/Study Quality in Duplicate	All studies that passed the initial title and abstract screen were double-coded by trained coders.
Supporting Judgments of Risk of Bias/Study Quality	Coders used information directly from the study to justify all decisions.
Providing Sources of Information for Risk of Bias/Study Quality Assessments	Coders collected and documented the source of information for each study quality assessment. They clearly documented what information comes directly from the report, what information was obtained from the author query, and what assumptions were made.
Differentiating Between Performance Bias and Detection Bias	Selection bias and attrition bias were assessed through our use of WWC Evidence Standards for Group Design, Version 3.0, with the former assessed through how groups are formed and the latter assessed for RCTs based on whether there is high attrition. Detection bias and reporting bias were assessed based on whether the authors reported all outcomes for which data were collected or only reported a subset (without justification).

Table F.1 (continued)

Criteria	Description
Risk of Bias Conduct (cont.)	*The following describes how this review complied with the* Adaptations on MECIR Version 2.2 Reporting Standards *on issues related to risk of bias.*
If Applicable, Assessing Risk of Bias Due to Lack of Blinding for Different Outcomes	This assessment is rare in TFA studies in particular and in education research studies in general because "concealment" of the intervention condition from the investigator or study participants or both, is rarely feasible or practical.
If Applicable, Assessing Completeness of Data for Different Outcomes	Within a study, completeness of data may be handled differently for different contrasts. RCTs with low attrition may use the missing data techniques outlined in the WWC *Procedures and Standards Handbook, Version 3.0*.
If Applicable, Summarizing Risk of Bias Assessments When Using the Cochrane Risk of Bias Tool	Not applicable because the Cochrane Risk of Bias Tool was not used.
Addressing Risk of Bias/Study Quality in the Synthesis	This review originally planned to use sensitivity analysis to check if the results were sensitive to the inclusion or exclusion of particular studies using a "one study removed" analysis. There were not enough studies to carry out this analysis.
Incorporating Assessments of Risk of Bias	Within each study, each contrast (intervention vs. comparison group on each outcome) was assigned a quality rating (see design quality row above) that incorporates the risk of bias.
Risk of Bias Reporting	*The following describes how this review complied with the* Adaptations on MECIR Version 2.2 Reporting Standards *on issues related to risk of bias.*
Risk of Bias and/or Study Quality Table	After assessing all design quality issues, the SCG required coders to select a study rating and a study disposition code (with an explanation) as follows:<br>• RCTs with no confounds, reliable outcomes, and low attrition received a rating of *meets design quality standards without reservations*.<br>• RCTs with high attrition and QEDs with no confounds, reliable outcomes, and baseline groups equivalence in analysis samples "received a rating of *meets design quality standards with reservations*.
Summary Assessments of Risk of Bias/Study Quality	
Study Quality/Risk of Bias in Included Studies	
